# In vitro inflammation and toxicity assessment of pre- and post-incinerated organomodified nanoclays to macrophages using high-throughput screening approaches

**DOI:** 10.1186/s12989-024-00577-7

**Published:** 2024-03-21

**Authors:** Todd A. Stueckle, Jake Jensen, Jayme P. Coyle, Raymond Derk, Alixandra Wagner, Cerasela Zoica Dinu, Tiffany G. Kornberg, Sherri A. Friend, Alan Dozier, Sushant Agarwal, Rakesh K. Gupta, Liying W. Rojanasakul

**Affiliations:** 1 Health Effects Laboratory Division, National Institute for Occupational Safety and Health, 1095 Willowdale Road, Morgantown, WV 26505 USA; 2 Chemical and Biomedical Engineering, West Virginia University, Morgantown, WV 26506 USA

**Keywords:** Nanoclay, Organic coating, Incineration, Silicates, In vitro models, High-throughput screening, Human lung cells, Mouse

## Abstract

**Background:**

Organomodified nanoclays (ONC), two-dimensional montmorillonite with organic coatings, are increasingly used to improve nanocomposite properties. However, little is known about pulmonary health risks along the nanoclay life cycle even with increased evidence of airborne particulate exposures in occupational environments. Recently, oropharyngeal aspiration exposure to pre- and post-incinerated ONC in mice caused low grade, persistent lung inflammation with a pro-fibrotic signaling response with unknown mode(s) of action. We hypothesized that the organic coating presence and incineration status of nanoclays determine the inflammatory cytokine secretary profile and cytotoxic response of macrophages. To test this hypothesis differentiated human macrophages (THP-1) were acutely exposed (0–20 µg/cm^2^) to pristine, uncoated nanoclay (CloisNa), an ONC (Clois30B), their incinerated byproducts (I-CloisNa and I-Clois30B), and crystalline silica (CS) followed by cytotoxicity and inflammatory endpoints. Macrophages were co-exposed to lipopolysaccharide (LPS) or LPS-free medium to assess the role of priming the NF-κB pathway in macrophage response to nanoclay treatment. Data were compared to inflammatory responses in male C57Bl/6J mice following 30 and 300 µg/mouse aspiration exposure to the same particles.

**Results:**

In LPS-free media, CloisNa exposure caused mitochondrial depolarization while Clois30B exposure caused reduced macrophage viability, greater cytotoxicity, and significant damage-associated molecular patterns (IL-1α and ATP) release compared to CloisNa and unexposed controls. LPS priming with low CloisNa doses caused elevated cathepsin B/Caspage-1/IL-1β release while higher doses resulted in apoptosis. Clois30B exposure caused dose-dependent THP-1 cell pyroptosis evidenced by Cathepsin B and IL-1β release and Gasdermin D cleavage. Incineration ablated the cytotoxic and inflammatory effects of Clois30B while I-CloisNa still retained some mild inflammatory potential. Comparative analyses suggested that in vitro macrophage cell viability, inflammasome endpoints, and pro-inflammatory cytokine profiles significantly correlated to mouse bronchioalveolar lavage inflammation metrics including inflammatory cell recruitment.

**Conclusions:**

Presence of organic coating and incineration status influenced inflammatory and cytotoxic responses following exposure to human macrophages. Clois30B, with a quaternary ammonium tallow coating, induced a robust cell membrane damage and pyroptosis effect which was eliminated after incineration. Conversely, incinerated nanoclay exposure primarily caused elevated inflammatory cytokine release from THP-1 cells. Collectively, pre-incinerated nanoclay displayed interaction with macrophage membrane components (molecular initiating event), increased pro-inflammatory mediators, and increased inflammatory cell recruitment (two key events) in the lung fibrosis adverse outcome pathway.

**Supplementary Information:**

The online version contains supplementary material available at 10.1186/s12989-024-00577-7.

## Background

Research and development efforts of nanoclay-enabled technologies have steadily increased due to their use in advanced manufacturing of plastic nanocomposites [1–3]. Little is known, however, whether nanoclay exposure poses an occupational health risk across its lifecycle with known high exposure levels (up to 39.2 mg/m^3^) [4–10]. Furthermore, in vitro nanoclay toxicity studies have been mostly restricted to epithelial cell responses with little effort to evaluate immune cell responses and immune system key events that lead to potential disease. There is a clear emerging need to further understand toxicological implications of nanoclay-enabled technologies across their life cycle to thus responsibly develop and implement safe nanoclay-based applications [6, 9].

One of the most popular nanoclays in advanced manufacturing, two dimensional (2D) smectite nanoclays (e.g. montmorillonite), possess a 2:1 phyllosilicate structure of an octahedral aluminum oxide sheet bounded by two tetrahedral silica oxide sheets on each side resulting in dimensions of 1 nm thick and hundreds of nm in length and width. Natural montmorillonite exists in a stacked mineral platelet morphology with hydrophilic properties. Addition of organic modifier coatings, such as quaternary ammonium tallow compounds (QACs), are used to improve nanoclay dispersion, rheology, barrier function, and thermal properties in composite materials [3, 11, 12].

Increased concern associated with ONC inhalation exposure in occupational settings along its lifecycle has driven pulmonary toxicity investigations. Inhalation of montmorillonite in miners resulted in a ‘silicosis-like’ lung pathology, most likely due to surface silica group reaction with lung tissue commonly associated with crystalline silica exposure [13, 14]. Addition of QAC coatings carries cell membrane and cytotoxicity hazards [15]. To address this, our group’s previous work conducted thorough physicochemical characterization on a library of pre- and post-incinerated nanoclays. Cloisite Na, a pristine, uncoated montmorillonite possesses single and stacked platelet morphology with a silicate (Si–O–Si) and aluminate (Al–OH–Al) surface chemistry. Its organic coating derivative, Cloisite 30B, possesses similar characteristics but with an additional methyl, tallow, bis-2-hydroxyethyl, quaternary ammonium coating. Incineration of these two nanoclays resulted in I-CloisNa and I-Clois30B that possessed deformations of the OH linked to Al^3−^ and Mg^2−^ and increased presence of Al–O group. In addition, I-CloisNa still possessed Si–O–Si surface chemistry and a smooth surface consistent with amorphous silica. I-Clois30B surface chemistry analysis showed complete disappearance of the quaternary ammonium tallow coating with a heterogenous surface containing smooth amorphous, porous, or fused stacked platelet morphology suggesting that the presence of the organic coating caused partial retention of platelet structure [16, 17].

Comparative in vitro studies of pre-versus post-incinerated Cloisite^®^ and Nanomer^®^ ONCs largely reported that toxicity was influenced by the presence and type of the organic modifier, as well as incineration status in bronchial and small airway epithelial cell models [16, 18–20]. Uncoated nanoclays and ONCs caused monolayer integrity loss and apoptosis with different dose response curves, suggesting different potency or modes of action. ONC exposure caused enhanced particle uptake, inconsistent changes in reactive oxygen species (ROS), mitochondrial degeneration, and increased genotoxicity (i.e., micronuclei and DNA breakage) [15, 21–23]. Conversely, incinerated nanoclays produced minimal effects [16, 18–20]. Our group’s in vivo pulmonary toxicity assessment of pre- and post-incinerated Cloisite® nanoclays [17], reported that ONC (Cloisite® 30B) exposure resulted in a low grade, delayed inflammatory response, consisting of increased macrophage and neutrophil infiltrations, elevated damage markers, and elevated pro-fibrotic cytokines at day 28. Conversely, Cloisite® Na^+^ caused a robust inflammatory response with the same profibrotic signature as the ONC-exposed animals. Incinerated Cloisite® Na^+^ elicited a transient inflammatory response while incinerated Cloisite® 30B produced a chronic inflammatory response, similar to aged crystalline silica (CS) [24]. Recent studies by Di Ianni et al. [25] reported that presence of QAC coating on bentonite decreased toxicity in exposed mouse lung; however, the same ONC caused potent in vitro epithelial and macrophage toxicity [26] which aligns with our assessments. Collectively, it remains unclear what role pulmonary macrophages play in eliciting these observed in vivo effects. Very few studies on nanoclay exposure impacts to the immune system and chronic inflammation have been conducted but are critical since these responses to particle deposition typically drive lung pathology [27, 28]. At present, no thorough evaluation exists of how nanoclay exposure impacts lung macrophage cellular and inflammatory responses which can promote pulmonary disease. Defining lung macrophage and other major target cell’s response to ONC exposure would identify those key events in an adverse outcome pathway (AOP) framework to improve screening strategies and inform risk assessment [29]. Furthermore, correlating in vitro response to in vivo metrics across particle types and characteristics can assist in placing in vitro models’ screening role in an integrated testing framework. A recent review identified the need for systematic in vitro/in vivo extrapolation studies, identification of immunomodulatory biomarkers, and characterization of changes in toxic effect over the nanoclay lifecycle [15].

Past attempts at using in vitro models to predict in vivo effect following inhalation particle exposures has resulted in marginal success complicated by non-standardized test methods, particle kinetics, dosimetry, and differences in clearance mechanisms; however, recent improvements have occurred with use of the AOP paradigm [30–32]. Traditionally, in vitro studies with ultrafine particles to assess macrophage inflammatory and toxicity responses used LPS-primed macrophages [33]. Co-stimulation of macrophages with LPS allowed for priming of the inflammasome via NF-κB activation to evaluate the potential role of the NLRP3 inflammasome in driving inflammatory and toxicity response [28, 34]. Furthermore, it simulates a relevant occupational exposure (typically containing microbes and endotoxin) and mimics other priming signals (e.g. TNF) from other exposed cells [28]. However, a majority of lung particle exposures with animal models are typically conducted using sterile particle preparations under relatively sterile laboratory conditions [35, 36]. The absence of LPS allows for comparison of particle-induced inflammatory response across relatively sterile in vitro versus in vivo particle exposures and can assess whether a particle can activate the inflammasome in the absence of LPS. There is an inherent lack of in vitro studies that performed direct comparisons between LPS- and non-LPS stimulated macrophages and how that compares to the in vivo animal model response.

To address these issues, we conducted a study to investigate acute toxicity responses, several key events (KE) in the proposed ‘substance interaction with the pulmonary resident cell membrane components leading to pulmonary fibrosis’ AOP #173 [37], and potential underlying mechanisms for lung macrophages following occupationally expected exposure levels to pre- and post-incinerated organomodified nanoclay. The objectives were to (1) assess how differences in coating and incineration status determines differentiated macrophage response, and (2) correlate in vitro macrophage endpoints to in vivo responses. We hypothesized that the organic coating presence and incineration status of nanoclays determine the inflammatory cytokine secretary profile and cytotoxic response of macrophages. Herein, we report on particle characterization and macrophage toxicity and inflammatory responses to help further understand toxic effects observed in vivo.

## Methods

### Particle preparation and characterization

To screen and evaluate coating and incineration effects on nanoclays’ potential for lung cell in vitro adverse outcomes, pristine (Cloisite® Na + ; CloisNa) and an organomodified nanoclay (Cloisite® 30B; Clois30B) were purchased from Southern Clay Products (Gonzalez, TX). To model particles generated during end-of-life municipal incineration, both CloisNa and Clois30B were subjected to incineration during thermogravimetric analysis (900 °C for 100 min) and collected as incinerated nanoclays I-CloisNa and I-Clois30B as previously described [16]. Based on dry particle diameter and chemistry, heat inactivated crystalline silica (CS; Min-U-Sil 5; US Silica; Berkeley Springs, WV) was used as a benchmark particle for all studies. Non-freshly fractured (i.e. aged) Min-U-Sil 5 particle samples were dry heated at 180 °C for several hours to remove endotoxin and held at room temperature until used. Aged CS loses most of its silicon and siloxyl radicals compared to freshly fractured silica, but still retains silica structure and some inflammatory potential similar to pyrogenic silica [14, 24, 38]. All particle samples were UV sterilized and fully characterized for water and organic modifier content, surface chemistry, particle morphology, and elemental analysis by thermal gravimetric analysis, Fourier transform infrared spectroscopy, field emission scanning electron microscopy, and energy-dispersive x-ray spectroscopy, respectively, in previous publications [16, 17].

Stock particle suspensions (3 to 5 ml at 1 mg/mL) were made by adding sterile MilliQ water to known particle mass in sterile glass test tubes. Discrete sonication was used to disperse stock particle suspensions. Briefly, particle suspensions were lightly vortexed and sonicated three separate times (200.4 J/ml per run) with a cup horn (Sonics VibraCell VCX-750; Newton, CT) at 40% intensity immersed in cold water with a one-minute rest between sonications. This achieved a total critical sonication energy (DSE_cr_ = 601.2 J/ml) needed to reach relatively stable suspensions of dispersed particles with lowest agglomerate formation [39]. Particle suspensions were immediately serially diluted in Roswell Park Memorial Institute + 10% FBS (RPMI) THP-1 cell culture medium. Media density, viscosity, and refractive index (Additional file 3: Table S1) were determined using a volumetric flask with balance, viscometer, and a refractometer following previously described methods (n = 3 independent experiments) [40] and were used as input parameters to calculate hydrodynamic diameter via dynamic light scattering analysis (DLS). Effective density (ρ_EV_) of each particle (0.1 mg/ml) in medium was measured using particle density, packed pellet volume, medium density, and theoretical stacking factor (SF = 0.634) as previously described (n = 3) [40, 41]. Soluble endotoxin in sonicated stock suspensions (n = 3) were determined with the Pierce LAL chromogenic method as previously described (ThermoFisher Scientific, Waltham, MA) [17].

DLS, particle dispersity index (PdI), and zeta potential analyses using a Zetasizer Nano ZS (Malvern, Malvern, UK) were also performed for each particle in water and culture medium described below. DLS analysis calculates hydrodynamic radius for low aspect ratio three dimensional particles, hence, it is not a reliable technique for determining 2D particle size in suspension [42]. Here, DLS was performed to make relative size comparisons among particle types. Particle suspension pH of each particle in medium was determined (n = 3) using a standard pH meter (Accumet Model 50, Fisher Scientific). Immediately after sonication, particles were diluted to 100 µg/mL and analyzed for size and zeta potential using a Universal Dip Cell (Malvern). Three runs per experiment and three independent experiments were performed. Since Clois30B particles were observed to re-agglomerate and fall out of suspension following dispersion in water [17], only the first runs off of each experiment were used for hydrodynamic diameter and PdI analyses. Likewise, incinerated nanoclay particles showed low suspension stability, hence, a similar analysis approach was taken. Values from each run were averaged (n = 3).

### X-ray diffraction

X-ray diffraction (XRD) analysis was used to assess spacing between nanoclay platelets and confirm the absence of platelet spacing in incinerated particle samples. For Bruker XRD analyses, dry powder samples were placed on glass slides using a thin layer of vacuum grease. Specifically, the powders were placed on top of the vacuum grease with excess powder being shaken off; this process was repeated until a thin layer of the powdered samples was obtained. The glass slides containing the samples were then mounted onto the sample holder via double sided tape and the Bruker D8 Discovery (Madison, WI) was aligned. Diffraction was obtained (n = 3) in the 2°–9.5° 2θ range at increments of 0.02° at a scan speed of 10 s/step. A Cu-kα1 8047.2 eV radiation source at 40 kV and 40 mA was used. Basal spacing was determined by Bragg’s equation:

$${\text{n}}\uplambda = {\text{2dsin}}\uptheta ,$$

where n is an integer, λ is the wavelength of the X-ray radiation (0.1546 nm), d is the spacing between lattice planes, and θ is the measured diffraction angle. The peak locations were determined via Match (Crystal Impact, Bonn, Germany).

Next, to evaluate crystallinity of pre-incinerated nanoclay and compare incinerated nanoclay structure to that of CS, XRD via an X’Pert Pro (Malvern PANalytical) was conducted by placing samples on glass slides in a thin, even layer which were then mounted in the sample holders. Diffraction was obtained (n = 3) in the 5°–100° 2θ range at a scan speed of 10 s/step. A Cu-kα1 8047.2 eV radiation source at 45 kV and 40 mA was used. XRD patterns were identified using X’Pert HighScore (PANalytical) or the RRUFF Project database.

### Cell culture

THP-1 monocytes were acquired from ATCC (Manassas, VA) and were cultured in ATCC-recommended Roswell Park Memorial Institute (RPMI) 1640 formulated medium supplemented with 10% fetal bovine serum (FBS), L-glutamine, 0.05 mM β-mercaptoethanol, 1 mM sodium pyruvate, and 1% penicillin/streptomycin (Sigma-Aldrich, St. Louis, MO). THP-1 cells were chosen as a suitable model based on its standardized use as a human macrophage model for particle and fiber toxicology studies [43]. Monocyte stock cultures were maintained between 2 to 8 × 10^5^ cells per mL for a maximum of one month in CellStar flasks (Fisher Scientific). For all microplate assays, monocytes between 4 and 10 passages were seeded at 2 × 10^4^ cells per well in a black-walled clear bottom tissue culture-treated 96-well plate (Corning, Corning, NY) in the presence of 1α, 25-dihydroxy-Vitamin D_3_ (Sigma-Aldrich) at 150 nM to differentiate into adherent cells for 48 h. Next, attached cells were exposed to 10 nM of phorbol, 12-myristate, 13-acetate (Sigma-Aldrich) for 12 h to complete differentiation. This protocol has been shown to minimize cell clumping and maximize cell response to stimuli [43, 44]. Confirmation of differentiated THP-1 macrophages was conducted (see Additional file 2). For particle exposure, attached differentiated THP-1 cells were briefly washed with warm (37 °C) phosphate-buffered saline (PBS) and then co-exposed with or without 10 ng/mL lipopolysaccharide (LPS; Sigma-Aldrich) and test particles at the reported particle dose ranges (described below) in quadruplicate. All water-soluble tetrazolium salt-1 (WST-1) and lactate dehydrogenase (LDH) assays in this study were performed in Falcon tissue culture-treated 96-well plates.

### Field emission scanning electron microscopy (FESEM) and transmission electron microscopy (TEM) analyses

FESEM imaging analyses were conducted to ascertain particle size and morphology in water and cell culture medium preparations following previously described procedures [17]. Briefly, each sonicated particle stock suspension (n = 3) was diluted into vehicle at 25–100 µg/mL, filtered onto polycarbonate filter, gold/palladium sputter coated, and imaged with a S-4800 FESEM (Hitachi, Tokyo). Particles in complex medium were diluted to 25 µg/mL with sterile filtered MilliQ water to reduce medium interference with particle imaging. 20–30 representative images were taken to compare to dynamic light scattering analyses. Length and maximum width for each located particle in digital FESEM images were measured in ImageJ v1.52 (NIH) with n = 104–437 per particle.

To qualitatively ascertain particle uptake and intracellular location into exposed cells, THP-1 cell were seeded at 5 × 10^5^ in 6-well plates overnight following published method [45]. Next, cells were exposed to each particle type at 0.6 µg/cm^2^ for 24 h in duplicate, rinsed with saline wash, trypsinized, collected, and centrifuged to collect a pellet of exposed cells. The pellet was resuspended in fixative, processed, blocked, sectioned, mounted on copper TEM grids, and imaged with a TEM (JEOL 1400, Tokyo, Japan). 20–30 images were taken to evaluate cellular uptake potential for each particle. Three independent experimental replicates were performed.

To confirm particle uptake prepared TEM grids were stabilized by carbon evaporation by coating them with a 20 nm carbon film using a Leica ACE 600 carbon coater. This stabilization was required for unsupported thin sections to withstand an exposure to the 200 keV Schottky field emission electron beam utilized in the NIOSH JEOL 2100F analytical TEM-STEM (scanning transmission electron microscope). EDS (energy dispersion spectroscopy) analysis with a silicon drift x-ray detector (Oxford Aztec) was performed in the STEM operating mode with a 1 nm electron probe. This operating mode was used to enable the precise analysis of a nanoparticle utilizing drift correction. The STEM operating mode is equipped with bright and dark field detectors with the EDS system allowed to access the dark field detector for compositional analysis of materials. The EDS system is capable of both point and 2-dimensional atomic compositional analysis to produce mapping of the spatial distributions of different atomic species. Intra-cellular nanoparticles were initially located using a low voltage (100 keV) TEM optimized for biological imaging. These intra-cellular nanoparticles were then relocated in the NIOSH analytical TEM/STEM system using micrographs from the previous TEM observations by matching the morphology of identified cells and their nuclei.

### WST-1 and LDH assays

Differentiated THP-1 cells were seeded at 2 × 10^4^ cells per well as described above. Next, cells were exposed to each freshly sonicated particle in 200 µl at 0.02–20 µg/cm^2^ (0.032–32 µg/ml) in quadruplicate for 24 h. The highest in vitro dose represented the OSHA permissible exposure limit (5 mg/m^3^) for respirable particles not otherwise regulated assuming an exposure of 45 year working lifetime, 240 days per year, and other particle and lung parameters [46]. THP-1 cells were exposed to LPS or LPS-free medium to ascertain their cell activity in the presence and absence of a robust inflammatory response. 1% Triton-X (Sigma-Aldrich) applied to cells for 2 h served as a positive 100% cytotoxicity control. Following exposure, plates were centrifuged at 1000 rpm for 5 min and the top 100 µl of supernatant was collected for lactate dehydrogenase (LDH) assay to measure cytotoxicity (Roche, Indianapolis, IN) according to manufacturer’s instructions. Next, 10 µL of WST-1 reagent (2-(4-iodophenyl)-3-(4-nitrophenyl)-5-(2,4-disulfophenyl)-2H-tetrazolium; Roche) was added to the remaining medium with cells and incubated for 2 h to measure cell viability. Absorbance of each well was read on a microplate spectrophotometer (SpectraMax Plus 384, Molecular Devices) at 490 and 450 nm for LDH and WST-1 assays, respectively. LDH interference assay was conducted by incubating 2.5 mU/well of purified LDH-A (Millipore-Sigma) in medium in the presence of serially diluted particle for 24 h. Absorbance values were corrected in medium only values and % interference calculated (Additional file 3: Table S2). All values for each particle and medium type were corrected with interference values. Four independent experimental runs were performed.

### Quantitative high-throughput screening analyses

Cells were seeded in black 96- or 384-well plates with clear bottom (Corning) at 2 × 10^4^ cells/well and exposed to applied doses of 0–20 µg/cm^2^ for each particle. A two-way 5 × 8 complete design was used for each discrete time point with a minimum of quadruplicate replicates per treatment. A mass per unit alveolar surface area dose metric was used to directly compare in vitro responses to in vivo pulmonary exposure responses [17]. Our in vivo study found that aspirated nanoclay and CS primarily deposited in the terminal bronchioles, alveolar ducts, and surrounding alveoli. The low (30 µg/mouse) and high (300 µg/mouse) dose in vivo exposures in a 500 cm^2^ alveolar epithelial surface area in a mouse lung [47] would equal an applied in vitro dose of 0.06 and 0.6 µg/cm^2^, respectively. This approach has limitations since it assumes 100% deposition of the aspirated mass and that alveolar macrophages are homogeneously dispersed throughout the deep lung. Thus, careful interpretation of in vitro to in vivo correlations must be performed.

THP-1 cells were exposed for 0–24 h and then assayed using quantitative HTS screening approaches via multiplex fluorescent automated imaging at 4 × , 10 × or 20 × magnification on an ImageXpress Micro XLS (Molecular Devices, Sunnyvale, CA) possessing a CMOS 16-bit digital camera. Endpoints and the multiplex fluorescent staining used are described in Table 1 and below. Assays were conducted based on modifications to previously described standardized methods or strictly following fluorescent dye manufacturers’ protocols [48, 49]. If recommended by dye manufacturer or to reduce high background signal, stained cells were washed with 37 °C fresh culture medium then imaged in clear Fluorobrite DMEM (ThermoFisher). Automated HTS imaging using one, four, or nine image replicate sites per well were taken for 4 ×, 10 ×, and 20 × magnifications, respectively. HTS image analysis was conducted as previously described [18] using several application models in MetaXpress v6.2 which allows for quantification of cell-specific fluorescent intensities and can remove non-cell specific staining and background interference [18, 50]. Particle-only control wells with same concentrations and dyes were imaged and used to subtract positively labeled particles in each assay. Following image quality control criteria, each measured parameter from each cell was averaged within site, replicate sites averaged within well, and exported to Excel for statistical analyses.

**Table 1:** Key event screening approach in THP-1 macrophages following pre- and post-incinerated organomodified nanoclay exposure

Key event	Stains^a^	Fluorescent channels
Live/dead	Propidium Iodide	DAPI, Cy5
Mitochondrial membrane potential	JC-1	DAPI, FITC, Cy3
Reactive oxygen species	CellRox Green	DAPI, FITC
Cell membrane damage^b^	LDH	n/a
Cell metabolism^b^	WST-1	n/a
Lysosome damage	Magic Red Cathepsin B	DAPI, Cy5
Caspase 1	FAM-FLICA Caspase 1	DAPI, FITC
Caspase 3/7	Cell Event/Propidium Iodide	DAPI, FITC, Cy5
Inflammation response	Multiplex Luminex beads	n/a

^a^All HTS assays, except WST-1 and LDH contained Hoechst 33,342 as a nuclear stain

^b^Non-HTS traditional assays

Live cell scoring using propidium iodide (PI) nuclear exclusion as a marker for live cells was used as a corroborative measure of cytotoxicity and proliferation with the WST-1 assay following pre- and post-incinerated nanoclay particle exposure. Following particle exposure, a 10 × concentration of Hoescht 33,342 (ThermoFisher) and PI (ThermoFisher) was added to exposure medium for a final working concentration of 1 µM Hoescht and 5 µg/ml PI. Triton-X at 0.1% acted as a positive cell death control while retaining the overall cell plasma membrane intact. Cells were incubated for 30 min at standard culture conditions (37 °C and 5% CO_2_ humid air) followed by imaging at 10 × magnification with 4 sites per well. Nuclei and PI were imaged with DAPI and Cy5 filters, respectively. Number and percentage of live and dead cells per site were determined and averaged per well within Multi-wavelength Cell Scoring (MCS) application module in MetaXpress, and then corrected for both unexposed and positive cytotoxicity controls to calculate percent survival. Data represent four technical replicates across a minimum of three independent experiments.

JC-1 stain (ThermoFisher) was used to measure changes in mitochondrial membrane potential (MMP) following particle exposure by calculating the ratio of red (J-aggregates, healthy mitochondria) to green (monomers, depolarized mitochondria) integrated intensities of labeled mitochondria in each cell. Following exposure, live cells were stained with a 20 × dye cocktail to achieve a working concentration of 1 µg/ml JC-1 and 1 µM Hoescht. Cells were incubated for 15 min as described above followed by careful removal of treatment medium, one wash in warm serum-free medium, and addition of 100 µl of warm medium per well for imaging. Positive control for MMP depolarization consisted of cells exposed to 10 µM valinomycin 30 min prior to imaging. J-aggregates and monomers were imaged with TRITC and FITC standard filters while nuclei were imaged with DAPI filter at 20 × magnification with 9 sites per well. TRITC and FITC integrated intensities of each cell were determined per site and averaged per well in MCS. Data represent four technical replicates across a minimum of three independent experiments.

Screening for changes of intracellular reactive oxygen species (ROS) following particle exposure was performed with CellRox Green (ThermoFisher Scientific) staining. Prior to staining, 100 µM of menadione (MP Biomedicals) was applied to four replicate wells for 30 min to serve as positive ROS controls. Following particle exposure cells were treated with 10 × dye cocktail with target concentrations of 5 µM CellRox Green and 1 µM Hoescht 33,342. Cells were incubated for 30 min as described above, centrifuged to pellet unattached cells, and immediately imaged, or fixed in 4% formaldehyde for 15 min at room temperature, washed, and held in sterile PBS for later imaging. Nuclei and intracellular ROS were imaged with DAPI and FITC filters at 10 × or 20 × magnification with 4 or 9 sites per well, respectively. Integrated intensity of the FITC signal was averaged for each site and within well in MCS, then averaged for treatment groups. Attempts to quantify ROS in LPS-stimulated THP-1 cells were unsuccessful due to fluorescent signal above maximum threshold, therefore only non-LPS stimulated THP-1 cell data is reported. Data represent four technical replicates across a minimum of three independent experiments.

Magic Red Cathespin B staining (Immunochemistry) was employed to assess lysosome damage, an initiation step in NLRP3 inflammasome activation, following the manufacturer’s instructions. Following exposure cells were directly stained with a concentrated stock solution to achieve a working concentration of 5% Magic Red dye with 1 µM Hoescht 33,342 in cell culture medium. Cells were incubated under normal culture conditions for 1 h. Nigericin exposed cells (10 µM for 4–24 h) served as a positive control during assay development with maximum staining occurring between 16 and 24 h exposure. Cell plates were briefly centrifuged at 125×*g* for 5 min and immediately imaged at 20 × magnification with 9 sites per well. Integrated intensity of the Magic Red stain was determined for each cell and averaged within site in MCS. Data represent four technical replicates across a minimum of three independent experiments.

Caspase 1 FLICA (Immunochemistry), CellEvent Caspase 3/7 (Molecular Probes), and propidium iodide screening assays were used to distinguish between pyroptosis, apoptosis, and necrotic cell death in differentiated macrophages following nanoclay particle exposure following manufacturers’ protocols. For Caspase 1 assay after 4–12 h particle exposure, cell plates were centrifuged for 125×* g* for 5 min and then each well gently aspirated with a multi-channel pipette. A 1:150 dilution of FLICA dye in serum-free medium was applied to each well followed by 1 h incubation at standard culture conditions. Cells were then centrifuged, media with dye gently aspirated, and washed with warm PBS. Cells were incubated again in serum-free medium with 10 µg/ml PI and 1 µM Hoescht 33,342 for another hour to remove unbound FLICA. Plates were then centrifuged, washed, and immediately imaged in 100 µl of warm PBS at 37 °C in the IXM system. Nigericin exposed cells (10 µM for 4–24 h) served as a positive control during assay development with maximum staining occurring between 16 and 24 h exposure. Cells were imaged with DAPI, FITC, and Cy5 filters at 20 × magnification with 9 sites per well. First, cells were identified as possessing intact membrane (nucleus PI−) or leaky membranes (nucleus PI+) using MCS. FLICA integrated intensity was calculated for each cell, averaged within site, and averaged among wells. Next, cells with no or low (< 20% unexposed cells) FLICA integrated intensity and PI-, high FLICA (> 20% control) with PI-, high FLICA with PI+, and no FLICA and PI+ were scored as healthy, early pyroptosis, late pyroptosis, and necrosis, respectively.

For Caspase 3/7 assay, a 10 × concentrated staining cocktail containing CellEvent, propidium iodide, and Hoescht 33,342 was prepared in warm PBS containing 5% fetal bovine serum. The dye cocktail was delivered to each well to reach 3 µM, 10 µg/ml, and 1 µM working concentrations for each dye, respectively, after 4–6 h following exposure. Stained cells were incubated for 30 min in standard culture and centrifuged at 125×*g* for 5 min to pellet detached apoptotic and necrotic cells. Cells were immediately imaged with DAPI, FITC, and Cy5 filter sets. First, cells were identified as possessing intact membrane (nucleus PI-) or leaky membranes (nucleus PI+) in MCS. Procedures to determine CellEvent intensity as a function of particle × dose and percentage of cells within healthy, early apoptosis, late apoptosis, and necrosis followed those described for Caspase 1 assay. Four independent experimental runs were performed.

### ELISA and western blot analysis

To assess macrophage acute inflammatory signaling, THP-1 cells were seeded at 1.5 × 10^5^ per well and differentiated in 6-well plates as described above. Cells were then exposed to each particle (0, 0.06, 0.6 or 6 µg/cm^2^) in the absence or presence of LPS for 24 h (n = 3). Next, plates were centrifuged at 125×*g* for 5 min to remove cell debris and the conditioned medium was collected and temporarily stored at − 80 °C. To evaluate release of damage-associated molecular pattern molecules (DAMPs) as evidence of the molecular initiating event (MIE) in the lung fibrosis AOP, thawed and diluted aliquots of non-LPS exposed supernatants were assayed for IL-1α via ELISA (R and D Systems) and ATP via firefly luciferase bioluminescence assay (ThermoFisher) following manufacturers’ protocols. Tetramethylbenzidine optical density and luminescent intensities were quantified on a spectrophotometer (Spectramax) and H1 Synergy (Biotek, Winooski, VT), respectively (n = 3).

Next, to evaluate release of pro-inflammatory mediators as the first KE in the AOP, thawed samples were assayed in duplicate using a multiplex inflammation chemokine/cytokine assay using magnetic beads (R and D Systems, Minneapolis, MN) following the manufacturer’s protocol. In addition to acute inflammation response targets used for the in vivo study, we expanded the multiplex array to characterize differentiated THP-1 cell inflammation response following pre- and post-incinerated nanoclay exposure. Samples were assayed for each target’s concentration via a Luminex system (MilliporeSigma, Burlington, MA) using serial dilution of the manufacturer’s standards. Data were averaged between replicates and tested for differences among treatment groups. Lastly, data were transformed to log_2_ fold change values, globally Z score normalized within targets and treatments, feature scaled between + 3 and − 3, and plotted in a heat map using 2 factor hierarchical clustering analysis in SAS JMP v13 (SAS Institute, Cary, NC).

Gasdermin D was recently identified as the key effector protein for inflammasome-mediated pyroptosis since its N-terminus cleavage product (GSDMD-NT) by caspases forms membrane pores [51]. To assess macrophage pyroptosis induction by nanoclays, differentiated THP-1 cells in 6-well plates at 1.5 × 10^6^ cells, co-exposed with LPS, were exposed to 0–20 µg/mL of each particle for 24 h in duplicate. A positive control for GSDMD cleavage in THP-1 cells was performed by incubation of THP-1 cells with 100 ng/mL LPS for 4 h, followed by a 2-h incubation with 15 µM nigericin. Next, cells were placed on ice for 5 min, rinsed with cold PBS, and lysed with cell lysis buffer (Invitrogen, Carlsbad, CA) containing protease cocktail inhibitor and PMSF. Cell lysates were scraped with a rubber policeman, collected into Eppendorf tubes, briefly sonicated, and allowed to complete lysis for 20 min on ice prior to storage at − 80 °C. Total protein concentrations were measured using BCA assay. Three independent experiments were conducted.

GSDMD-NT expression analysis was conducted using a SimpleWes protein expression system (ProteinSimple, San Jose, CA) using capillary electrophoresis and antibody incubations. Briefly, 1 µg of each protein lysate from nanoclay-exposed differentiated THP-1 cell was placed onto loading plate along with gel matrix solutions, immobilization buffers, 1:50 dilution primary Gasdermin D antibody (Novus Biologicals, Littleton, CO), 1:200 dilution of secondary anti-rabbit streptavidin horseradish peroxidase (HRP) antibody (Cell Signaling Technology, Danvers, MA). Protein in the capillaries was separated by size via gel electrophoresis followed by incubation with primary and secondary HRP antibodies. Digital imaging of chemiluminescent bands was performed and bands at 55 and 38 kD were quantified via Compass software (Protein Simple, Wallingford, CT). Change in GSDMD-NT were calculated as percent band intensity of the cleaved product of total cleaved and full length GSDMD band intensities (n = 3).

### Animals

All animal data herein was collected as part of our previous in vivo study that reported on the pulmonary inflammatory and histopathological effects following oropharyngeal aspiration of a single bolus of 30 µg and 300 µg/mouse of each particle to male C57Bl/6J mice (Jackson Labs, Bar Harbor, ME) [17]. All procedures for this animal study were reviewed and approved by the CDC-Morgantown Institutional Animal Care and Use Committee and were conducted in an AAALAC-accredited facility.

### Cytospin analysis

To qualitatively evaluate in vivo uptake of each particle type by alveolar macrophages and approximate intracellular location, phase contrast imaging of fixed cells from bronchoalveolar lavage (BAL) was conducted (n = 8) as previously described [17]. Briefly, a cannula was inserted into the esophagus of euthanized mice, attached to a sterile syringe, then followed by the addition 0.8 ml of ice cold phosphate-buffered saline to the entire lung. Mild compressions of the chest were performed as fluid was collected into the syringe. Lavage was performed 4 times and centrifuged to pellet collected cells. BAL cells were resuspended in saline and counted. Collected BAL cells were immobilized via Cytospin, fixed and stained with a HEMA3 stain kit (Fisher Scientific), and imaged with a Leica DM2500 equipped with an Olympus DP73 digital camera. Images were evaluated for intracellular and extracellular particulate and compared to both in vitro TEM images and in vivo stained histological sections of lung.

### In vitro*–*in vivo* correlation analyses*

To ascertain whether in vitro THP-1 macrophage response to nanoclay particle exposure aligns with lung inflammation metrics from in vivo models, several sets of correlation analyses were conducted. Three different in vitro dose equivalents of the in vivo dose were used to assess the robustness of correlating the THP-1 macrophage effects to in vivo inflammatory metrics. THP-1 macrophage cell viability, in both the presence and absence of LPS, at 0.06 and 0.6 µg/cm^2^ (dose equivalent), 0.2 and 2 µg/cm^2^ (threefold dose equivalent), or 0.6 and 6 µg/cm^2^ (tenfold dose equivalent) for each particle was correlated to BAL cell differentials in 30 and 300 µg-exposed male C57Bl/6J mice (with 500 cm^2^ lung surface area) at Day 1 and Day 7 from our previous study [17]. Next, cathepsin B intensity, gasdermin D cleavage level, and IL-1β were correlated to BAL cell differentials. Next, each particle’s 16 inflammatory cytokine profile in THP-1 macrophages, in both the presence and absence of LPS, was correlated to the same corresponding cytokines for each corresponding dose level (0.06 µg/cm^2^ and 30 µg/lung; 0.6 µg/cm^2^ and 300 µg/lung; or 6 µg/cm^2^ and 300 µg/lung) at Day 1 and Day 7 post-exposure in vivo time point in C57Bl/6J-exposed mice [17]. Lastly, each cytokine level in THP-1-exposed cells was correlated to BAL corresponding levels across particle types. Correlation analyses are described below.

### Statistical analyses

Dose–response curves were modeled and fitted using the “drc” package [52] using the R v. 3.6.1 statistical program (R Foundation for Statistical Computing, Vienna, Austria). The effective dose to cause a 50% reduction in the measured response (ED_50_) compared to the control and associated estimate standard deviation were derived from fitted models using the “drc” package. ED_50_ values that exceeded the highest dose test (20 μg/cm^2^) were designated as > 20 μg/cm^2^. Statistical comparisons between modeled ED_50_ were performed via a two-sample *t* test. Where appropriate, defined ED_50_ value comparisons against particle effective doses designated as > 20 μg/cm^2^ were performed using a one-sample t-test, wherein the null value was set to 20. Differences in particle characteristics in media were determined by one-way analysis of variance (ANOVA). To determine differences in macrophage responses among treatment groups, 2-way ANOVAs were conducted. All data were evaluated for variance homoscedasticity and normal distribution of residuals (α = 0.05) prior to conducting an ANOVA. Data not meeting these ANOVA assumptions were tested with a Wilcoxon or Kruskal–Wallis test. If ANOVAs indicated a difference, Tukey–Kramer HSD post-hoc tests were run to identify those treatment groups different from others. For correlation analyses, all THP-1 cell and BAL mouse data were transformed to fold change compared to their respective unexposed controls and then log-transformed for data normalization. Pearson’s coefficient correlation analyses were run for data with normal distributions to assess potential in vivo predictive value of these in vitro screening approaches. Spearman’s coefficient correlation analysis was performed for data sets without a normal distribution. If a particle treatment elicited a low number of significantly different cytokines compared to unexposed cells, correlation analysis was not conducted. All other data were analyzed with JMP SAS (version 13) software.

## Results

### Physicochemical characterization of pre- and post-incinerated nanoclays

**Fig. 1:**
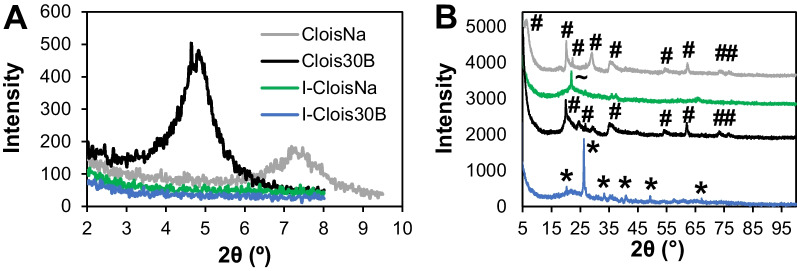
Structure and dispersed particle size analyses of nanoclays **A** X-ray diffraction analysis of pristine and organomodified montmorillonite nanoclays (ONC) and their incinerated byproducts. Basal spacing of nanoclays in the 2–9.5° 2θ range indicated that ONC (Clois30B) possessed greater spacing between clay platelet structure than pristine (CloisNa) nanoclay, confirming presence of the organomodifier coating. Both incinerated particles showed no evidence of platelet spacing structure. **B** Diffraction patterns determined that both pre-incinerated nanoclays exhibited montmorillonite structure (#). Incinerated ONC possessed quartz crystal structure (*) while I-CloisNa exhibited amorphous silica structure (~)

**Fig. 2:**
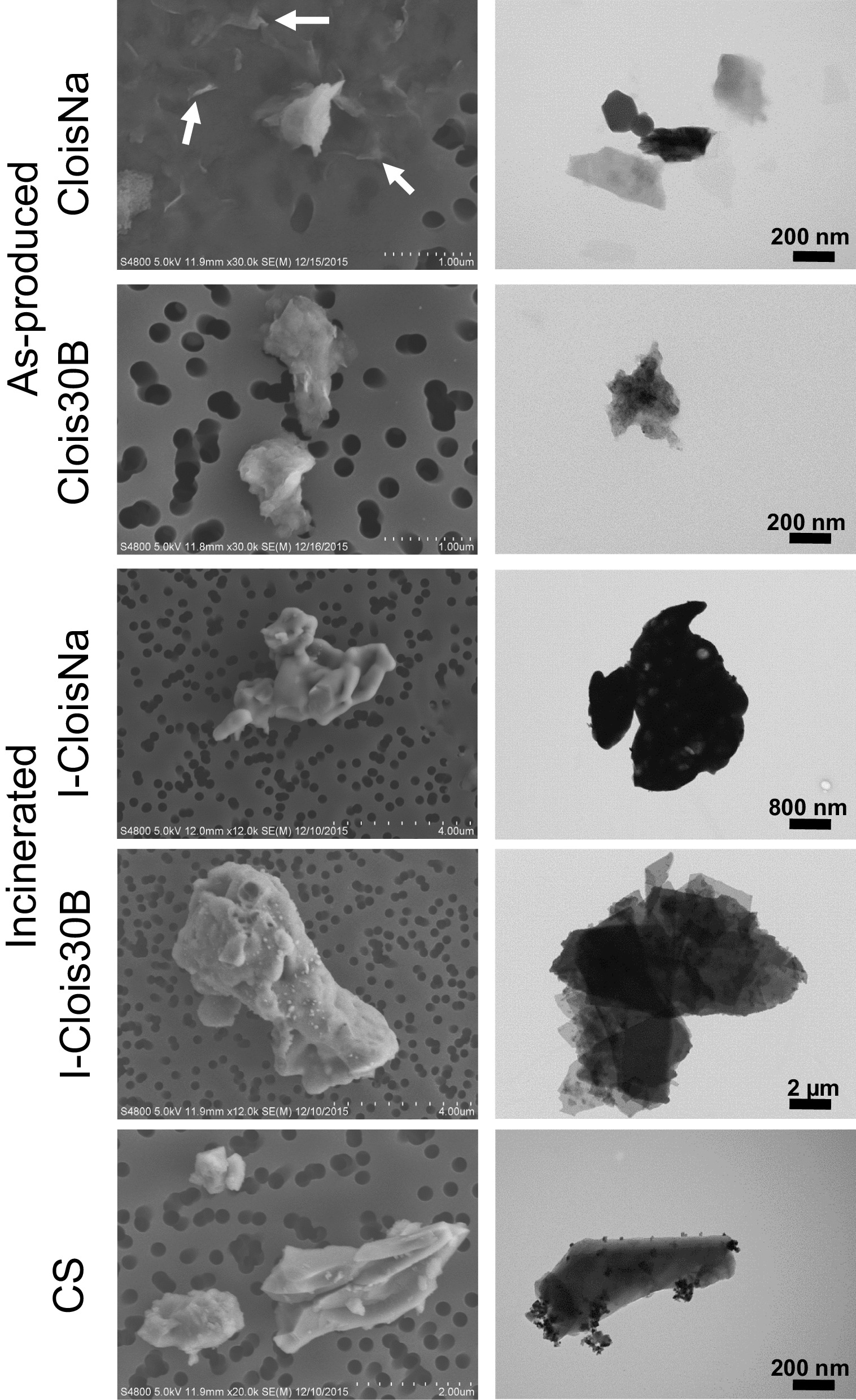
Pre- and post-incinerated uncoated (CloisNa) and organomodified (Clois30B) montmorillonite nanoclay particle morphology under FESEM and TEM imaging. As-produced nanoclays possessed exfoliated (white arrows) and stacked platelet morphology while I-CloisNa showed amorphous pyrogenic silica morphology. I-Clois30B possessed a mixture of amorphous and retention of stacked platelet morphology, similar to crystalline silica (CS)

All particle characteristics in dry and culture medium suspension are summarized in Table 2. XRD and FESEM analyses indicated that both CloisNa and Clois30B were montmorillonite with dioctahedral structure and stacked platelet morphology. Clois30B displayed increased basal spacing compared to CloisNa indicative of the organic QAC coating (Fig. 1A, B; Table 2). Dispersed CloisNa in RPMI medium exhibited single or stacked platelet morphology similar to that observed in water (Fig. 2; Additional file 1: Figure S1). CloisNa showed the significantly smallest mean length and width (medians 294 and 174 nm) compared to all other particles (Additional file 3: Table S3) with DLS size data matching FESEM data. CloisNa dispersed well (i.e. low PdI) and possessed the lowest ρEV of all particles tested (Table 2). Clois30B exhibited a range of size distributions, compared to CloisNa, and consisted of small sub-micron or large single micron stacked platelet morphology with size median of 304 nm. I-CloisNa possessed a smooth, amorphous quartz silicon oxide structure (Fig. 1B) with an electron-dense, compacted appearance (Fig. 2). This differed from I-Clois30B’s quartz silicon oxide structure which exhibited morphologies including smooth, pocketed, [16, 17] and platelet-like structures, although at significantly larger sizes than Clois30B. Incinerated particles showed comparable sizes (mean width ranging 1636 ± 98 to 1731 ± 108 nm) with DLS data comparing to FESEM data. Incinerated nanoclays showed comparable ρ_EV_ ranging 1.26 to 1.39. Dispersed CS had a fractured, crystal-like morphology and displayed single micron size with comparable ρENM and ρ_EV_ to incinerated nanoclays. DLS hydrodynamic diameter was 40% smaller than FESEM width measurements. Zeta potentials were slightly negative (− 6.8 to − 10.3) with minimal changes in pH across all tested particles. Lastly, all endotoxin levels in particle stock suspensions were below the kit’s lower detection limit (Additional file 3: Table S4). Additional details on physicochemical characterization are presented in Supplemental Material.

**Table 2:** Particle characteristics of pre- and post-incinerated nanoclay in THP-1 cell culture medium

	CloisNa	Clois30B	I-CloisNa	I-Clois30B	CS
XRD analysis					
Basal spacing (nm)	1.21	1.85	Not present	Not present	n/a
Crystal structure	Montmorillonite; dioctahedral	Montmorillonite; dioctahedral	Amorphous quartz SiO_2_	Quartz	Quartz
RPMI + 10% FBS					
SEM morphology	Single or small stacked platelets	Small or large stacked platelets	Smooth, amorphous	Amorphous; pocketed; platelet-like	Fractured, crystal-like
SEM mean length (nm)	379 ± 24	1270 ± 193	2872 ± 186	2392 ± 133	953 ± 36
SEM mean width (nm)	237 ± 15	857 ± 129	1731 ± 108	1636 ± 98	618 ± 24
Z average (nm)*	294.6 ± 3.1	205.7 ± 29.9	1795 ± 14.6	1495 ± 940	369.7 ± 7.51^a^
PdI*	0.388 ± .020	0.43 ± .035	0.417 ± .025	0.569 ± .077	0.535 ± .062
ζ*	− 10.3 ± .4	− 6.8 ± .3	− 9.8 ± .3	− 10.1 ± .4	− 9.0 ± .4
pH*	7.80 ± 0.02	7.82 ± 0.0	7.76 ± 0.07	7.83 ± 0.01	7.78 ± 0.01
Particle density ρENM (g/cm^3^)	2.86	1.98	2.196^†^	2.65^†^	2.65
Mean effective density ρEV (g/cm^3^)^b^	1.066 ± 0.001^A^	1.244 ± 0.018^B^	1.389 ± 0.020^DE^	1.264 ± 0.010^BC^	1.373 ± 0.018^DE^
THP-1 cellular uptake	Endosomes	Endosomes, some loss of intact endosome	Endosomes, acellular	Endosomes, acellular	Endosomes

SEM values represent means ± 1SE (n = 108–437)

*Values represent means ± 1SE of independent experiments (n = 3–6)

^a^Value did not agree with SEM measurements

^b^A theoretical stacking factor of 0.634 was used to calculate ρE

^A to E^Different capital letters indicate those ρEV significantly different from each other (*p* < 0.05)

^†^Not determined; estimated density based on XRD data and [105]

### Confirmation of THP-1 cell differentiation and T_H_1 polarization

Differentiation of THP-1 cells into attached macrophages was confirmed with increased CD11b positive staining and lysosomes compared to THP-1 monocytes (Additional file 1: S2A and B; see Supplementary Results). LPS stimulation of macrophages produced a T_H_1 macrophage inflammatory response compared to naïve macrophages (M_o_) evidenced by increased TNFα, CCL2, IL-1β, IL-6, and IL-8 (Additional file 1: Figure S2C).

### TEM and enhanced darkfield analysis of particle cellular uptake

**Fig. 3:**
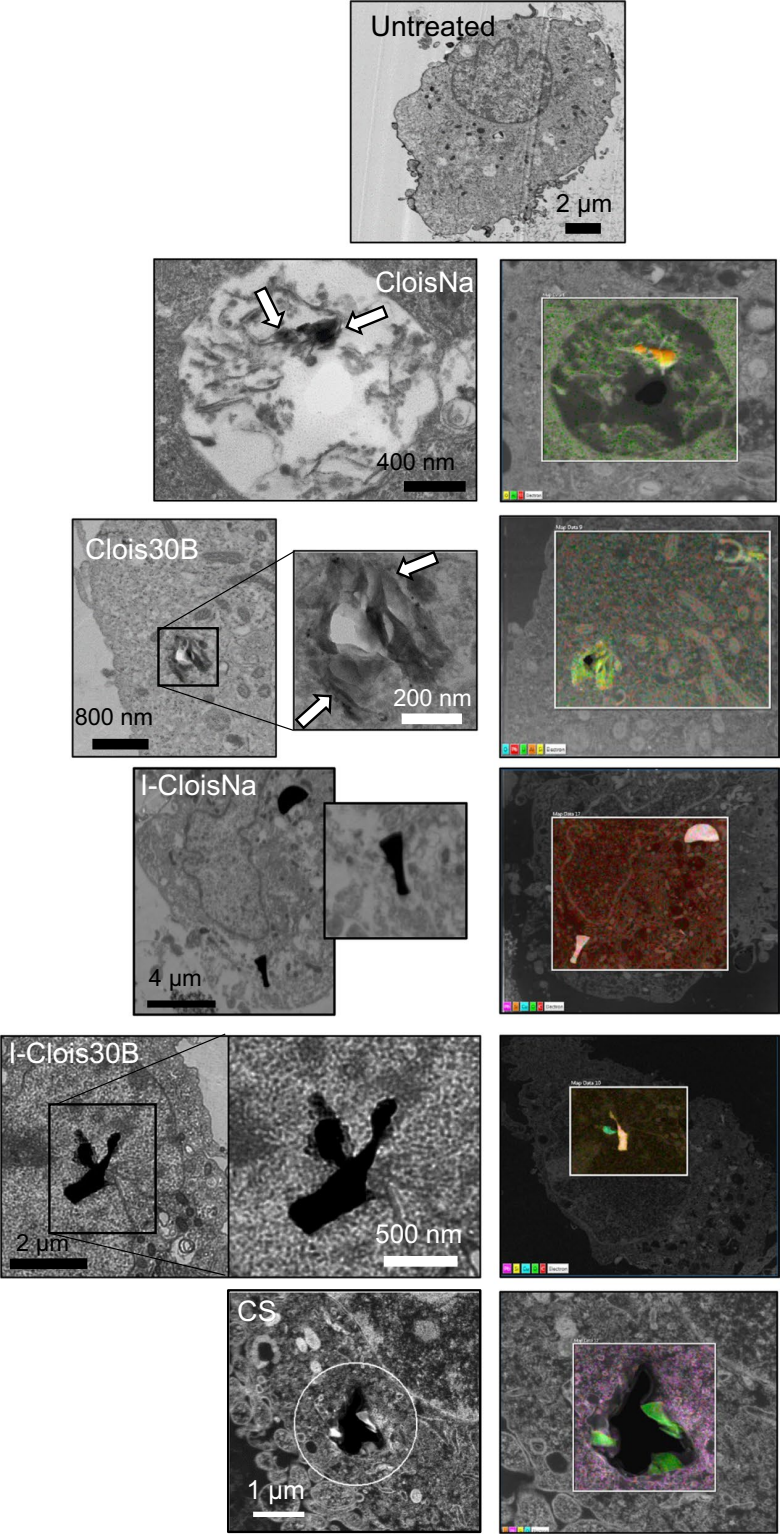
Uptake and localization of pre- and post-incinerated uncoated (CloisNa) and organomodified (Clois30B) montmorillonite nanoclay in differentiated THP-1 macrophages in the absence of LPS. Cells were exposed to a 0.6 µg/cm^2^ dose in 6-well plates for 24 h, followed by trypsinization, centrifugation, pellet fixation, sectioning, and imaging under TEM and TEM-STEM. Both bright and dark field detectors were used to collect images and EDS analyses. All nanoclay particles (white arrows) and crystalline silica were confirmed using EDS elemental analysis (n = 3; Additional file 1: Figures S3-7)

Following 24 h exposure, robust amounts of single and stacked platelets of CloisNa were found in endosomes of differentiated THP-1 cells (Fig. 3). CloisNa appeared as a dense platelet shaped particles or as thin, dense platelets. To a lesser extent, Clois30B particles were also found in endosomes with instances of disappearance of an intact endosome membrane. Elemental mapping with TEM-EDS confirmed particle uptake in THP-1 cells (Additional file 1: Figures S3-7). Only smaller particle fractions (< 2 µm) of I-CloisNa and I-Clois30B were observed in THP-1 cell endosomes while larger single micron fractions were found in acellular areas or alongside THP-1 cells. Notably, I-CloisNa exposed THP-1 cells possessed large endosomes. CS was found within THP-1 cell endosomes indicating adequate uptake ability. These results paralleled those observed in BALF Cytospin samples at Day 1 post-aspiration exposure in C57BL/6J mice (Additional file 1: Figure S8). CloisNa, Clois30B, and CS particles were located within collected alveolar macrophages. Both incinerated nanoclays particles were found engulfed by a single macrophage or extracellularly associated with several macrophages. Collectively, this indicated that sub-micron, pre-incinerated nanoclay particles were more likely to undergo macrophage uptake, enclosed in endosomes, and interact with intracellular components.

### Particle type-dependent differential THP-1 macrophage cytotoxicity and inflammatory response

**Fig. 4:**
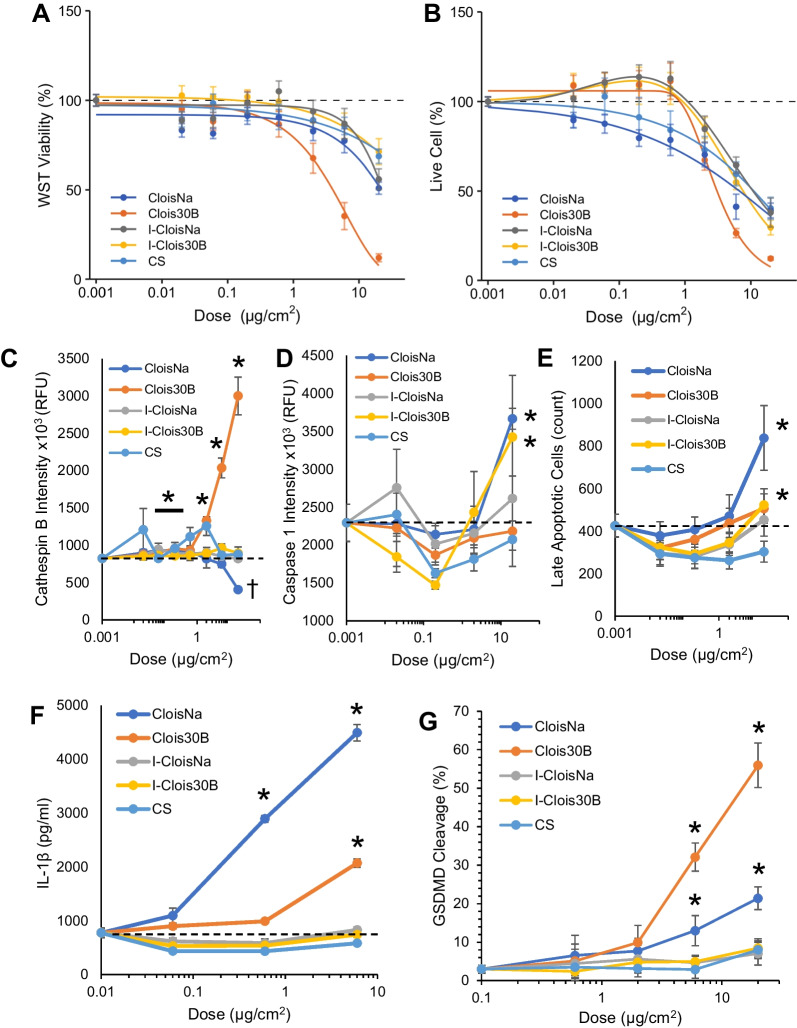
Quantitative HTS toxicity data of differentiated THP-1 cells exposed to pre- and post-incinerated organomodified nanoclays in the presence of LPS. **A** Cell viability via WST-1 assay and B) live/dead counts at 24 h indicated that Clois30B produced a robust cytotoxic effect on THP-1 cells compared to all other particles. **C** Cathepsin B release, **D** Caspase 1, and **E** late apoptosis in exposed THP-1 cells at 6 h post-exposure **F** Secreted IL-1β levels from nanoclay-exposed THPs at 24 h. **G** Gasdermin D N-terminus (GSDMD) cleavage product indicating inflammasome-induced pyroptosis. All HTS measures reflect mean ± SE integrated intensities. Horizontal dotted line indicates unexposed control level. * and † indicates those treatments with a significant increase or decrease from unexposed cells, respectively (*p* ≤ 0.05, n = 3–4)

**Fig. 5:**
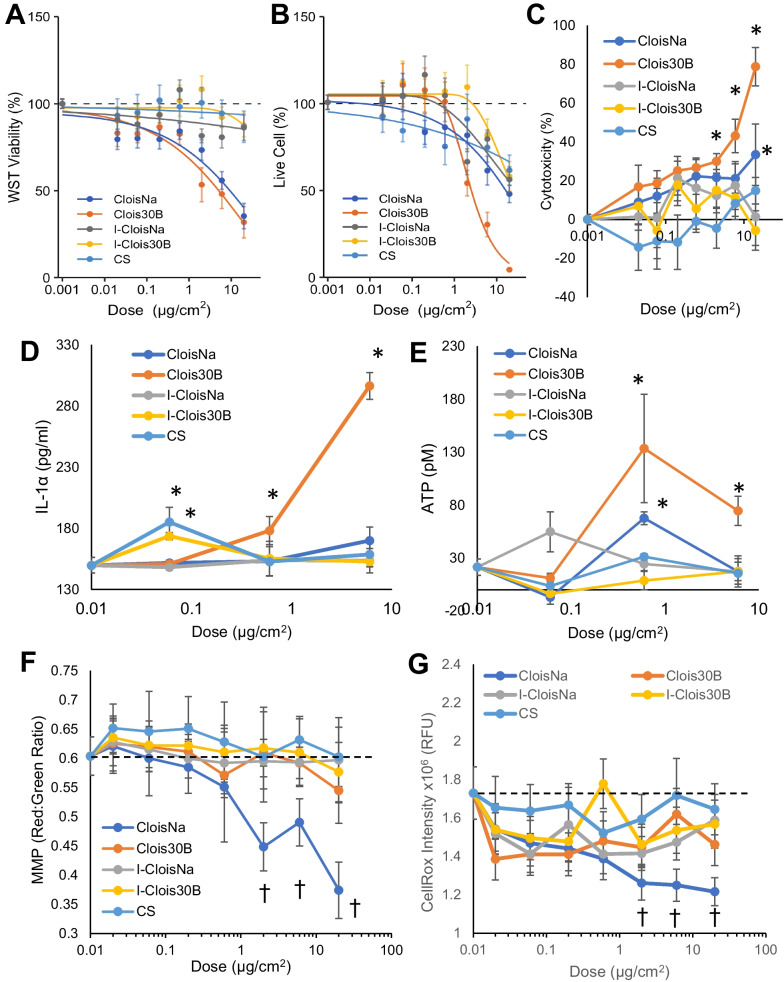
Differentiated THP-1 macrophage cell metabolism, DAMP release, mitochondrial membrane potential, and ROS at 24 h post-exposure in the absence of LPS. **A** CloisNa and Clois30B exposure both reduced THP-1 cell WST-1 metabolic activity. **B** CloisNa and Clois30B exposure caused a dose-dependent decrease in live cell counts while **C** Clois30B exposure caused a dose-dependent increase in cytotoxicity (LDH). **D**, **E** Clois30B produced dose-dependent release of DAMPs (IL-1α and ATP) while all other particles had significant minimal or no effect. **F** Only CloisNa caused a dose-dependent mitochondrial membrane depolarization. **G** Intracellular reactive oxygen species using CellROX. Points represent mean ± SE. Horizontal dashed bar represents unexposed cell level. * and † indicated significant increase and decrease compared to unexposed cells, respectively (*p* ≤ 0.05, n = 4)

Since all aspirated particles displayed a high propensity to interact with macrophages in in vivo exposures, we conducted quantitative HTS in vitro screening approaches to identify potential modes of action for macrophage cytotoxicity and inflammation responses in the absence and presence of LPS. Clois30B exposure caused significant decreased cell viability (4.8-fold) compared to all other particles with the largest maximal response (> 80% at highest dose; Clois30B ED_50_ = 4.1 µg/cm^2^; all others ED_50_ > 20 µg/cm^2^). Comparatively, all other nanoclays caused dose-dependent reductions in WST-1 at doses > 0.6 µg/cm^2^ at 24 h. Clois30B exposure retained this potent cell viability reduction ability without LPS co-exposure (Clois30B ED_50_ = 5.4 µg/cm^2^; Fig. 4A, Table 3). LPS-primed THP-1 cells were less sensitive to CloisNa-induced loss of cell viability compared to unprimed cells (ED_50_ = 11.1 µg/cm^2^). These results were largely reflected in live cell imaging analysis (Fig. 4B; Additional file 1: Figure S9), though the threshold of cell viability loss was generally higher at > 0.6 µg/cm^2^. Clois30B exposure caused significantly greater toxicity (3.2- to 4.7-fold) than both incinerated nanoclays and CS (p ≤ 0.02), with a non-significant increase compared to CloisNa value (*p* = 0.053; Table 3; Additional file 1: Figure S10). Without LPS co-exposure CloisNa and Clois30B exposure resulted in significant loss of cell viability (58% and 87% at 20 µg/cm^2^, respectively) compared to controls as measured by WST-1 metabolism and live cell imaging with minimal shift in induction threshold (Fig. 5A, B; Table 3). Only 20 µg/cm^2^ CloisNa showed a significant 33% increase in cytotoxicity (i.e. LDH) while Clois30B exposure caused a robust, dose-dependent increase in cytotoxicity (≥ 2 µg/cm^2^; Fig. 5C). Incinerated nanoclays and CS showed minimal effect.

**Table 3:** ED_50_ comparison (µg/cm^2^) of pre- and post-incinerated nanoclay exposures to differentiated human THP-1 macrophages in the presence or absence of LPS

Assay	Particle	With LPS	Without LPS
WST-1	CloisNa	> 20*^,a^	11.1 ± 3.5
	Clois30B	4.1 ± 0.7	5.4 ± 2.0
	I-CloisNa	> 20*^,a^	> 20*^,†,a^
	I-Clois30B	> 20*^,a^	> 20*^,†,a^
	CS	> 20*^, a^	> 20*^,†,a^
Live cell imaging	CloisNa	6.8 ± 2.00	15.2 ± 6.9
	Clois30B	2.4 ± 0.5^b^	2.9 ± 0.6^b^
	I-CloisNa	11.1 ± 2.5*	> 20*^,a^
	I-Clois30B	7.6 ± 1.4*	> 20*^,a^
	CS	11.1 ± 03.5*	> 20*^,a^

^*^ and ^†^denote those particles with significantly different ED_50_ values from Clois30B and CloisNa, respectively (*p* ≤ 0.05, n = 4 independent experiments)

^a^Estimated ED_50_ value was greater than the highest tested dose (20 µg/cm^2^). Standard error of the mean values were not reported for these estimates

^b^Clois30B exhibited a non-significant lower ED_50_ than CloisNa with (*p* = 0.053) and without (*p* = 0.062) LPS

Since some nanoclays produced acute loss of cell viability, we evaluated nanoclays for release of DAMPs as evidence of interaction of nanoclay with the cell membrane, which is the MIE in the lung fibrosis AOP. Clois30B exposure caused a dose-dependent increase in IL-1α and ATP, two well-established DAMPs, with significant levels at ≥ 0.6 µg/cm^2^ (Fig. 5D, E) compared to unexposed cells. I-Clois30B and CS caused a significant moderate increase in IL-1α at 0.06 µg/cm^2^ while only CloisNa caused a significant increase in ATP at 0.6 µg/cm^2^. All other treatment groups showed no significant effect.

Next, CloisNa exposure caused a dose-dependent decrease (≥ 2 µg/cm^2^) in THP-1 cell mitochondrial membrane potential (MMP) while all other particles had no effect when compared to unexposed cells. This effect was mirrored in a dose-dependent drop in intracellular ROS production in CloisNa-exposed cells (Fig. 5F, G). Notably, most CloisNa-exposed THP-1 cells were still observed on plates while Clois30B-exposed cells were clearly absent at high toxic doses with clear evidence of cell debris, potentially suggesting complete cell lysis or at least detachment of THP-1 cells at the time of measurement. Since engineered nanomaterial exposures are associated with apoptosis induction and/or inflammasome activation in vitro [53], treated THP-1 cells were assessed for markers of inflammasome activation resulting in pyroptotic cell death and apoptosis via caspase 3/7 activity. Analysis showed that CloisNa exposure induced a mixed pyroptotic and apoptotic profile, as determined by moderate significant increase in cathepsin B release (0.06 and 0.2 µg/cm^2^), caspase 1 activity, markers of caspase 3/7 activity, and extracellular IL-1β release. Clois30B-treated THP-1 cells, by contrast, induced significant dose-dependent elevations in putative markers of the pyroptotic cell death pathway: Cathepsin B release, GSDMD-NT cleavage, and IL-1β release (Fig. 4C–G; Additional file 1: Figure S11). CloisNa exposure caused a significant, but intermediate increase in GSDMD-NT compared to untreated controls, while IL-1β levels were significantly higher than Clois30B-treated cells. Incinerated nanoclay analogues or CS did not cause acute pyroptotic or apoptotic cell death in LPS-stimulated THP-1 cells. Collectively, these findings suggest that differences in surface chemistry on pre-incinerated nanoclay impact mode of cell death in exposed macrophages.

**Fig. 6:**
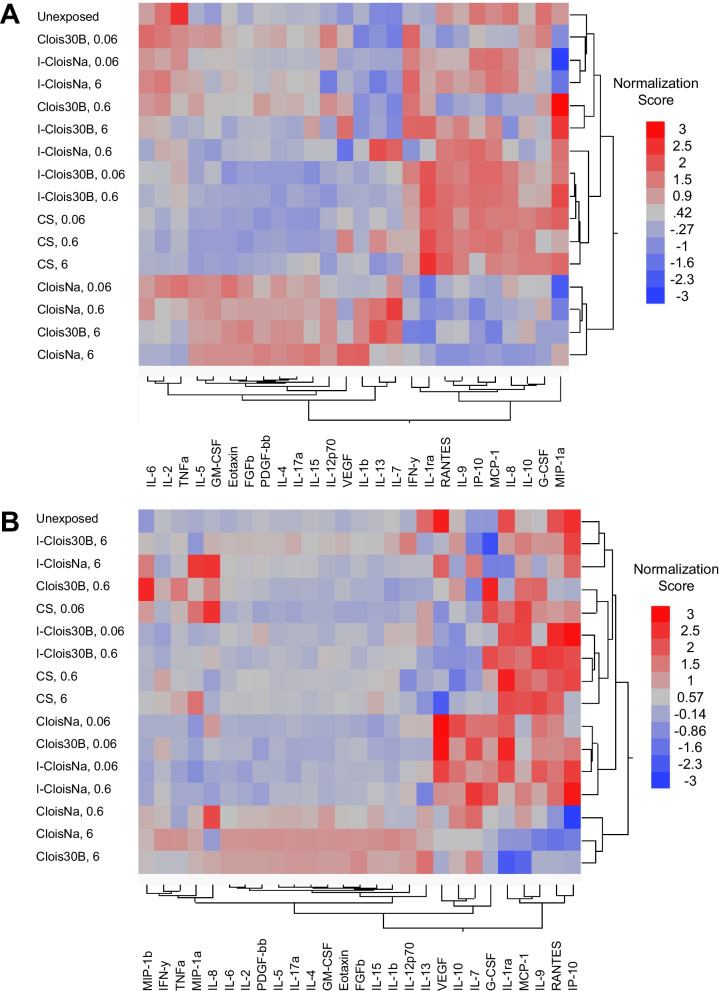
Secreted cytokine response profile of differentiated THP-1 macrophage cells following pre- and post-incinerated nanoclay exposure for 24 h (n = 3). **A** Cells exposed to particles in the presence of LPS. Pre-incinerated nanoclay exposure caused an inflammatory profile which differed from post-incinerated nanoclays and crystalline silica (CS). **B** Cells exposed to particles in the absence of LPS

Given the differences in THP-1 in vitro cell death mechanism and the differential inflammatory response in vivo, multiplex analysis of secreted or released in vitro cytokines and chemokines following acute nanoclay exposure (0–6 µg/cm^2^) was conducted to evaluate differences in THP-1 cell inflammatory signaling, increased secretion of pro-inflammatory mediators (KE1), and their correlation to in vivo effect. Particle co-exposure with LPS produced a response dominated by IL-1β secretion with magnification of cytokine release at lower doses (Fig. 6A). IL-1β, IL-7, and IL-13, known key drivers of leukocyte recruitment, clustered together with their over-expression primarily associated with higher pre-incinerated nanoclay doses. Dose-dependent cytokine release following CloisNa and Clois30B exposures including those associated with acute inflammation (IL-1β, IL-7), IL-17α, T_H_2 (IL-4, IL-13, eotaxin) and pro-fibrotic responses (PDGF-ββ, FGFβ; Additional file 3: Table S5). This large cluster of cytokines was more responsive to Clois30B than CloisNa at 0.06 µg/cm^2^, showed minimal response to incinerated nanoclay exposure, and showed decreased secretion (20–25%) in response to CS exposure. LPS co-stimulation caused moderate doses of both nanoclays to elicit elevated acute damage (IL-6), T_H_2 (IL-5, GM-CSF), and IL-15, while elevated IL-1ra was alone elevated following Clois30B exposure. Next, a group of cytokines (IP-10, MCP-1, IL-8, and IL-10) with decreased secretion (63–90%) was primarily associated with pre-incinerated nanoclays. Acute incinerated nanoclay and CS exposure produced minimal response compared to LPS-only exposed cells. Lastly, non-significant elevated IL-1ra levels were primarily associated with I-Clois30B and CS exposure. In a similar experiment of pre-incinerated nanoclays in the absence of LPS, an acute inflammatory response cytokine cluster (MIP1α, MIP1β, IFNγ, TNFα, and IL-8) displayed a different expression pattern across particle type compared to a large T_H_1/T_H_2/T_H_17 cytokine cluster (Fig. 6B). CloisNa and Clois30B produced the most similar secreted mixed T_H_1/T_H_2/T_H_17 cytokine profile with noticeable clustering of cytokines with similar functions. Dose-dependent significant increases in acute pro-inflammatory mediators (IL-1β, MIP1α, MIP1β), acute cell damage and T_H_1 (IL6, IL-12p70), T_H_2 and T_H_17 allergic response (IL-4, IL-5, eotaxin, GM-CSF, IL-17α), STAT3 pathway-associated T cell regulation (IL-2 and IL-15), and wound healing/pro-fibrotic (FGFβ, PDGF-ββ) signals were observed (Additional file 3: Table S6). Significant 1.3- to 7.1-fold increases occurred with IFNγ, TNFα, VEGF, IL-7, and IL-10, while decreases in RANTES and IP-10 were only observed with increasing doses of CloisNa. Conversely, the incinerated nanoclay exposure produced a comparatively muted response and possessed strikingly different profiles than pre-incinerated forms, with I-CloisNa clearly the most acutely inflammatory. Only the 6 µg/cm^2^ dose of both incinerated nanoclays produced noticeable similar acute inflammatory versus T_H_1/T_H_2/T_H_17 cytokine clusters. Notably, both IL-5 and IL-6 were secreted across all doses of I-CloisNa. I-CloisNa was also more acutely inflammogenic (e.g., TNFα, IL-6, IL-1β, IL-5) than CS, though both dampened VEGF secretion. CS exposure produced a minimal response with increased MIP-1β and non-significant increase in MIP1α. A summary of differentiated THP-1 cell response to pre- and post-incinerated nanoclays is presented in Table 4.

**Table 4:** Summary of THP-1 macrophage acute toxicity and inflammation response to pre- and post-incinerated organomodified nanoclay exposure in the presence or absence of LPS

Endpoint	Clois Na	Clois 30B	I-CloisNa	I-Clois30B	CS
Crystal structure	Montmorillonite	Montmorillonite	Amorphous quartz	Crystalline quartz	Crystalline quartz
Mean particle size in RPMI (µm)	0.304	1.064	2.302	2.104	0.786
Cellular uptake	+++; endosome	++; endosome	+; endosome	+; endosome	ND
w LPS					
WST-1 viability^a^	> 20	4.11	> 20	> 20	> 20
Live cell count^a^	6.82	2.36	11.06	7.56	11.06
LDH cytotoxicity (%)	n/a	n/a	n/a	n/a	n/a
Cathepsin B	**1.1**	**2.47**	1.05	1.17	1.05
Caspase I	**1.60**	− 1.05	1.14	**1.49**	− 1.11
Late apoptosis	**1.97**	1.19	1.07	**1.23**	− 1.41
IL-1β	**5.80**	**2.67**	1.07	− 1.04	− 1.33
Gasdermin D cleavage (%)	**13.00**	**32.11**	4.62	4.93	2.89
Inflammatory profile	IL-1β, T_H_1/T_H_2/T_H_17	IL-1β, T_H_1/T_H_2/T_H_17	Minimal	IL-1ra	IL-1ra
w/o LPS					
WST-1 viability^a^	11.13	5.41	> 20	> 20	> 20
Live cell count^a^	15.32	2.91	> 20	> 20	> 20
LDH cytotoxicity (%)	14.5	**74.6**	22.5	16.4	13.6
IL-1α	1.14	**1.97**	1.06	**1.16**	**1.24**
ATP	**3.19**	**6.2**	2.56	− 6.0	1.46
MMP	**-1.23**	− 1.02	− 1.02	1.01	1.05
Reactive oxygen species	− 1.39	− 1.06	− 1.18	− 1.12	− 1.01
Inflammatory profile	T_H_1/T_H_2/T_H_17	T_H_1/T_H_2/T_H_17	T_H_1/T_H_2/T_H_17	MIP-1β, TNFα, IL-2, Eotaxin, FGFβ	MIP-1β

All data represent mean fold change compared to unexposed cells unless otherwise noted

^a^Calculated EC_50_ values (µg/cm^2^)

+++ high; ++ moderate; + modest. *ND* not determined. *n/a* not applicable

Bold font indicates a significant difference compared to unexposed controls

n = 3–4 independent experiments

### In vitro THP-1 macrophage response correlation to in vivo BAL inflammatory indices

**Fig. 7:**
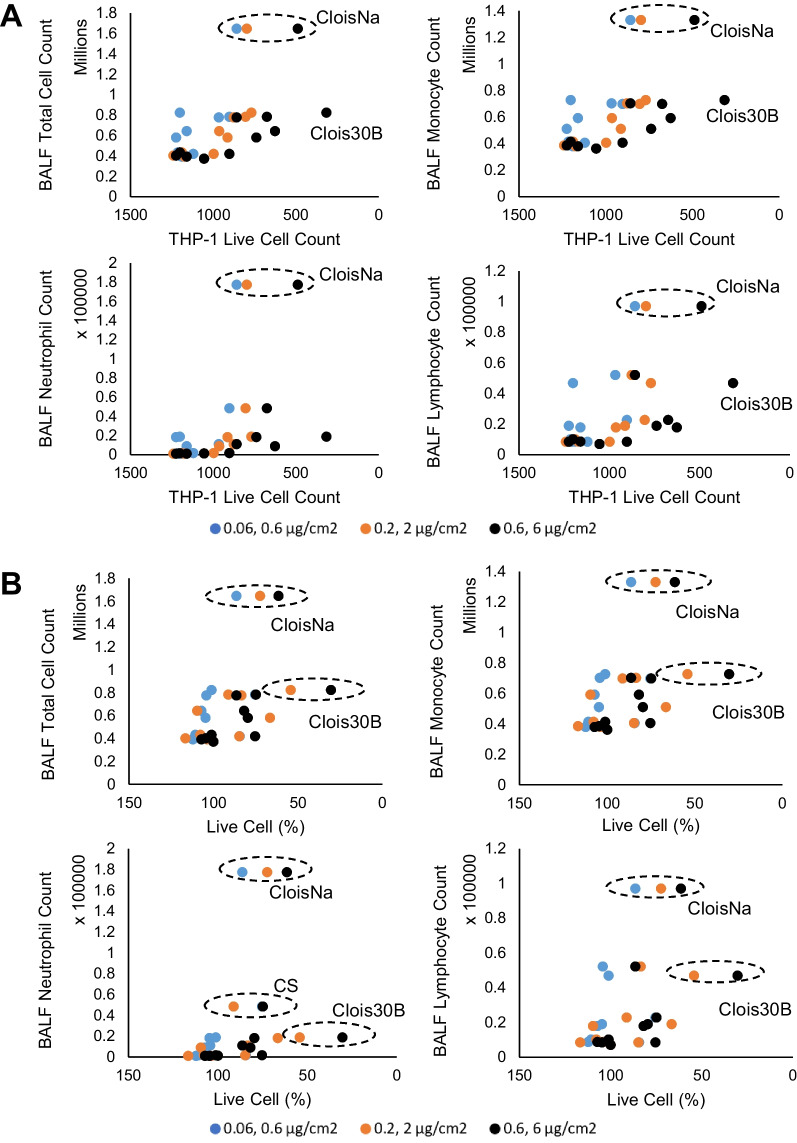
Representative plots of **A** LPS and **B** non-LPS stimulated THP-1 macrophage cell viability metrics significantly correlated to BAL cell differentials at Day 7 post-exposure following pre- and post-incinerated nanoclay particle exposure. Data from all particle types are presented. Most significant correlations were found with THP-1 live cell count in LPS stimulated cells (**A**) and non-LPS stimulated cells (**B**; Additional file 3: Table S7). Decreases in live THP-1 macrophages correlated with increased BAL immune cell counts. Comparisons were made at three different in vitro dose equivalent ratios (blue 0.06 and 0.6 µg/cm^2^; orange 0.2 and 2 µg/cm^2^; black 0.6 and 6 µg/cm^2^) versus in vivo aspiration doses (30 and 300 µg /lung) in male C57Bl/6J mice. Particle names and hashed ovals call out noticeable differences between CloisNa, Clois30B, and CS responses

In vitro to in vivo comparison analyses across all tested particles found that WST-1 and live cell count response in a tenfold in vitro dose scheme (0.6 and 6 µg/cm^2^) above the equivalent in vivo dose (30 and 300 µg/lung) produced significant moderate correlations with in vivo BAL total cell, monocyte, and lymphocyte counts in Day 7 post-exposure animals, regardless of LPS presence in THP-1 cultures (Fig. 7; Additional file 3: Table S7). In addition, non-LPS THP-1 cell LDH release at equivalent in vitro doses (0.06 and 0.6 µg/cm^2^) significantly correlated with in vivo BAL total cell and neutrophils on Day 1 and 7, and with monocytes and leukocytes on Day 7. Correlation of non-LPS THP-1 cell viability to Day 1 BAL cell differential endpoints was not significant, except for neutrophils at tenfold dose equivalent. Notably, the addition of LPS co-stimulation lowered the in vitro dose range for in vivo correlation with significant correlation of live cell count occurring with neutrophils. Both CloisNa and Clois30B treatments showed the strongest reliance on this trend. Next, gasdermin D cleavage and IL-1β levels, but not Cathepsin B release, in THP-1 in vitro model strongly correlated with in vivo BAL total cell, monocyte, neutrophil, and lymphocyte counts at Day 7 (Additional file 1: Figure S12, Additional file 3: Table S8). Notably, in vitro IL-1 β levels correlated with Day 1 BAL total cell and monocyte counts, with LPS co-stimulated cells showing a moderate to strong correlation at equivalent mass doses. Cathepsin B release strongly correlated with Day 28 BAL total cell, monocyte, and lymphocyte data most likely due to the low response profile at this late point.

**Fig. 8:**
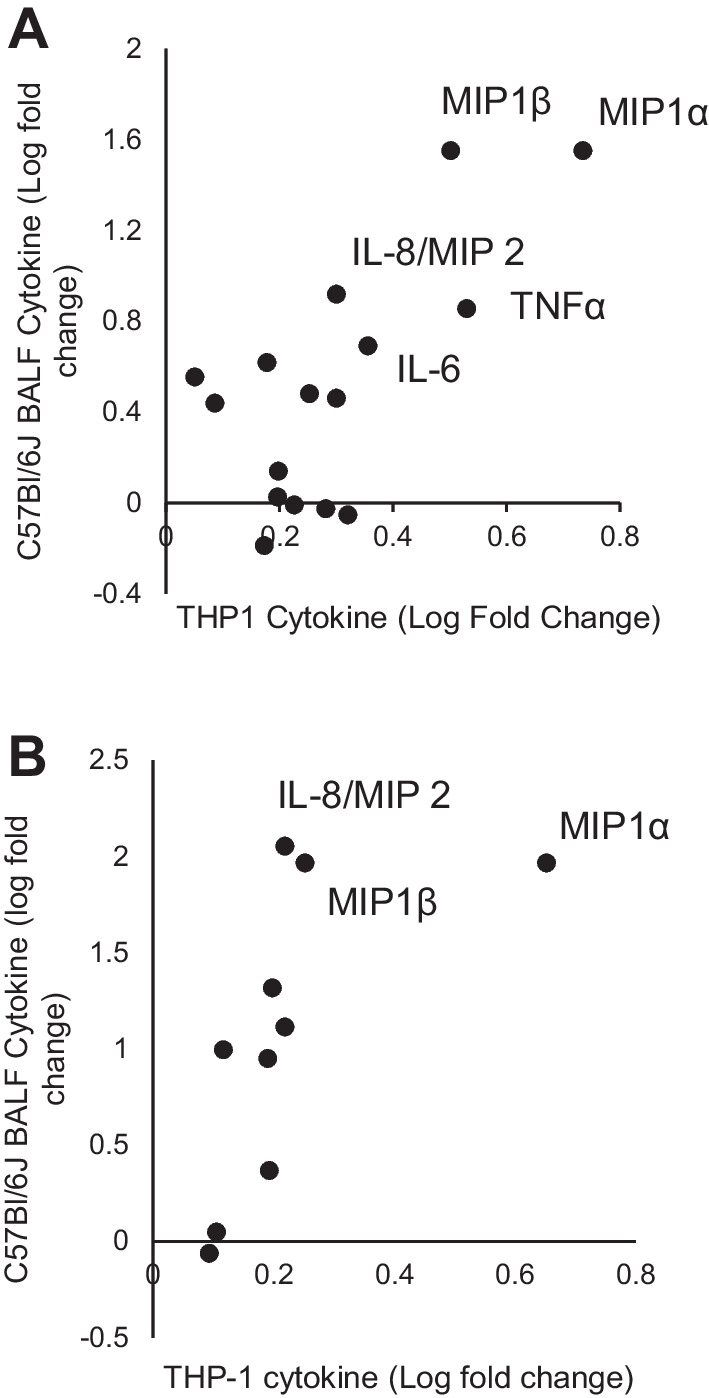
Representative plots of significant in vitro macrophage/in vivo BAL inflammatory cytokine correlation. **A** THP-1 cell cytokine profile after 24 h Clois30B 0.6 µg/cm^2^ exposure correlation with 300 µg Day 1 post-exposure BAL cytokine inflammatory profile in male C57Bl/6J mice. **B** I-CloisNa 6 µg/cm^2^-exposed THP-1 profile correlation with 300 µg Day 1 BAL profile in male C57Bl/6J mice. All other comparison correlations were not significant

Next, Clois30B in vitro 24 h exposure at 0.6 µg/cm^2^ to non-LPS stimulated THP-1 macrophages produced a cytokine response significantly correlated with the equivalent 300 µg/lung response in Day 1 exposed mouse lung (Fig. 8A; Table 5). MIP-1α, MIP-1β, TNFα, and IL-8/MIP2 were the cytokines most responsive to both models for Clois30B, thus indicating their potential role as inflammatory markers in future ONC toxicology studies. I-CloisNa at 6 µg/cm^2^ animals showed significant positive correlations with 300 µg/lung at Day 1 post-exposure (Fig. 8B). MIP-1α, MIP-1β, and IL-8/MIP2 showed elevated levels in both models. All other particles showed no significant correlation relationships between models or particle treatments did not elicit significant THP-1 cell cytokine production. Co-stimulation with LPS showed no significant correlation of in vitro versus in vivo pro-inflammatory cytokine response (Additional file 3: Table S9).

**Table 5:** Correlation coefficients of equivalent dose non-LPS stimulated THP-1 cells versus C57Bl/6J BALF (300 µg) at Day 1 and 7 post-exposure for 16 pro-inflammatory cytokines

	CloisNa	Clois30B	I-CloisNa	I-Clois30B	CS
24 h 0.6 µg/cm^2^ versus Day 1 300 µg/lung					
r	0.16	**0.69**	n/a	n/a	n/a
*p* value	0.56	0.003*	–	–	–
24 h 6 µg/cm^2^ versus Day 1 300 µg/lung					
r	− 0.07	0.33	**0.88**^**a**^	n/a	n/a
*p* value	0.81	0.22	**0.001**	–	–
24 h 0.6 µg/cm^2^ versus Day 7 300 µg/lung					
r	0.33	0.44	n/a	n/a	n/a
*p* value	0.21	0.08	–	–	–
24 h 6 µg/cm^2^ versus Day 7 300 µg/lung					
r	− 0.15^a^	0.01^a^	0.38^a^	n/a	n/a
*p* value	0.58	0.97	0.14	–	–

^*^Indicates those log-transformed correlations that were statistically significant (*p* ≤ 0.05)

^a^Non-parametric data; Spearman’s coefficient correlation analysis was performed

n/a indicates correlations not performed due to treatments producing minimal statistically significant THP1 cytokines

Correlation analyses for each cytokine between the two models for nanoclay particles revealed that equivalent in vitro dose 0.6 µg/cm^2^ at 24 h versus in vivo 300 µg/lung Day 1 post-exposure resulted in no significant correlations. Increasing the in vitro dose tenfold (6 µg/cm^2^) showed significant strong positive correlation with in vivo Day 1 for IL-6, Eotaxin, PDGF-ββ, and IL-12p70 (Additional file 3: Table S10). Macrophages exposed to 0.6 µg/cm^2^ for 24 h showed significant, strong positive correlations to 300 µg/lung Day 7 post-exposure for IL-1β, IL-10, and PDGF-ββ. Strong positive correlations (non-significant) also existed for IL-6 and eotaxin (*p* ≤ 0.06). Most of these significant correlations were driven by increased cytokine expression from both pre-incinerated nanoclays with minimal responses from incinerated nanoclays or CS. In summary, among all particle types, in vitro macrophage loss of cell viability, gasdermin D cleavage, IL-1β release, and a select number of cytokines correlated with in vivo BAL inflammation responses.

## Discussion

**Fig. 9:**
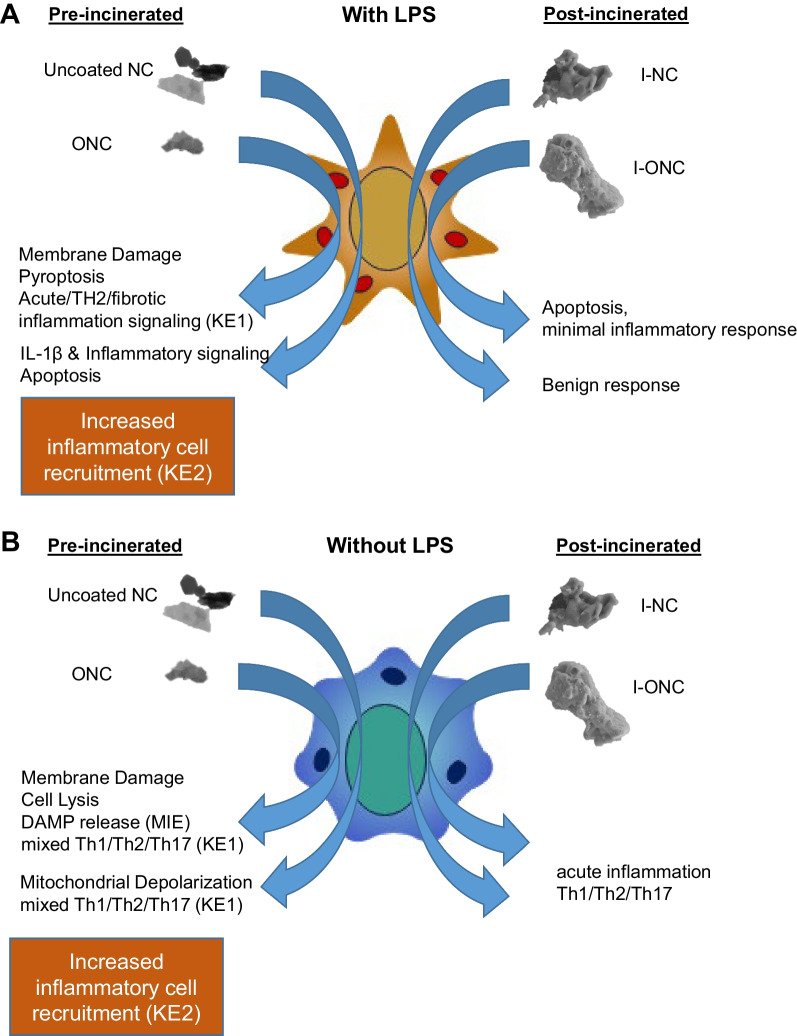
Proposed airway macrophage response to pre- and post-incinerated nanoclay particles in the **A** presence or **B** absence of lipid polysaccharide and its role in pulmonary fibrosis AOP. With LPS co-stimulation, uncoated nanoclay (NC) exposure caused a robust inflammatory response (KE1) and apoptosis while ONC exposure caused membrane damage, pyroptosis, and an acute T_H_2 inflammation (KE1) signal. Incinerated nanoclay exposure resulted in apoptosis, minimal inflammatory (I-ONC), or a relatively benign (I-NC) response. In a sterile exposure model uncoated NC and ONC exposure caused DAMP release (MIE), mitochondrial membrane depolarization and cell lysis, respectively, along with a mixed T_H_1/T_H_2/T_H_17 pro-inflammatory response (KE1). Incinerated nanoclay exposure collectively produced an acute mixed T_H_1/T_H_2/T_H_17 inflammatory response. These findings for pre-incinerated nanoclays aligned with increased inflammatory cell recruitment in the in vivo model (KE2)

The HTS in vitro screening and protein secretion profiling studies support the hypothesis that physicochemical characteristics, namely surface coating and incineration status, of nanoclays along their life cycle influence inflammation and cytotoxic effects in human macrophages. In addition, these findings correlated with previous in vivo inflammatory responses, thus identifying macrophage as a key cell type that modulate adverse effects following pre- and post-incinerated nanoclay particle exposure. These findings match the molecular initiating event (substance interaction with lung cell membrane components) and first key events (KE1 increased pro-inflammatory mediators, KE2 increased inflammatory cell recruitment) in the proposed lung fibrosis AOP #173 [37]. A summary of this study’s findings with and without LPS stimulus is presented in Fig. 9. Lastly, the findings suggest that in vitro model using human alveolar macrophage can serve as a potential screening tool for acute nanoclay in vivo exposure-induced lung toxicity.

Using both non- and LPS co-exposure assisted in identifying key factors driving macrophage response to nanoclay exposure. Other particle studies including metals, silica, and PM2.5 report that LPS, alarmins (e.g. TNFα, IL-1α), and exogenous pathogen molecular patterns act to prime the macrophage inflammasome to produce a IL-1β and IL-18 inflammatory response [28, 54, 55]. LPS priming allowed us to identify underlying mechanisms driving inflammatory response. By using non-LPS exposed macrophages, we identified nanoclay exposure-induced release of DAMPs, altered mitochondrial polarization, and improved correlations with our in vivo study’s inflammatory response findings.

Pre-incinerated nanoclays were more acutely toxic to macrophages compared to post-incinerated nanoclay and CS particles matching findings previously reported [16, 19]. However, dose–response curves among particles differed based on the assay indicating potential differences in mode of toxic action due to the presence/absence of a QAC coating. CloisNa exposure (i.e. no organic coating) resulted in uptake of nanoclay particles in endosomes, caused decreased cell viability, and moderate cell membrane damage (i.e. LDH release) indicating the cell death mechanism was potentially more dependent on internal cell death mechanism, and less than plasma membrane lysis. Alternatively, Clois30B exposure reduced THP-1 cell viability which coincided with LDH and DAMP (i.e. IL-1α, and ATP) release suggesting that plasma membrane damage is a potential mode of action for ONC-induced macrophage toxicity, which aligns with previous studies [56, 57] and confirms the MIE in the lung fibrosis AOP [37].

Our results indicate that moderate CloisNa exposure aligns with a NLRP3 inflammasome-mediated macrophage pyroptosis which involves endocytosed particle damaging phagolysosome membranes, increasing permeability, releasing proteolytic enzymes (e.g. Cathespin B), thereby initiating the NLRP3 inflammasome, Caspase 1 cleavage [17], gasdermin D cleavage [58], and IL-1β release [27]. The observed release of ATP into conditioned medium also potentially acted as an inflammasome activation signal through P2X_7_ receptor resulting in K^+^ ion efflux [58], a known rate limiting step inflammasome activation. Silica, carbon nanotubes, and titanium nanobelts were shown to induce lysosome membrane permeability and downstream cathepsin B activation of NLRP3 inflammasome [59]. Silica nanoparticles induce the NLRP3/Caspase-1/GSDMD pathway in cardiomyocytes in both in vivo and in vitro inhalation exposure models. Large graphene oxide, a 2-D nanoparticle, exposure to Kupffer cells cause lipid peroxidation following phagocytosis, calcium flux, mitochondrial ROS generation, and NLRP3/Caspase-1/GSDMND-mediated pyroptosis [54, 55]. Here, higher doses of CloisNa, however, caused MMP loss which coincided with apoptosis/necrosis and cell viability dose–response curves. Cathepsin B release is known to regulate Bcl2 degradation, thus increasing Bax/Bak pore formation in the outer mitochondrial membrane, mitochondrial depolarization, and subsequent apoptosis [60–62]. Damaged mitochondria can occur following particle-induced phagolysosome leakage (e.g. carbon black nanoparticles) or direct particle damage (e.g. residual fly ash, nano-ZnO) to mitochondria which enhances NLRP3 inflammasome formation [63–67]. Mitochondrial depolarization has been described as a decision point for apoptotic signaling [68]. Past studies with nanosilica and quartz particulate exposure reported loss of MMP, increased mitochondrial ROS production, and increased apoptosis [69, 70]. Elevated intracellular ROS appears to not play a role in THP-1 cell inflammatory or cytotoxicity responses following pre-incinerated nanoclay exposure and matches previously reported in vitro and in vivo findings [15, 17, 19, 57, 71]. This mode of action is well-established for other silicates and aluminosilicates [14] and contributes to their pro-inflammatory effect.

Clois30B particle uptake without an intact endosome membrane in surviving macrophages suggests potential membrane damage or passive uptake of these particle with a hydrophobic coating. Previous studies hypothesized that dissolution of the QAC coating into physiological fluids or in vitro cell culture medium is the initiating event for ONC exposure-induced toxicity [15, 22, 72]. Solubilization of QAC coating from Clois30B was previously observed in cell culture medium suspensions [23, 72]. Cationic lipids, such as QACs, can interact with lipid membranes resulting in endosome disruption, ROS production, and cell necrosis [73]. However, filtration studies reported that a wide diversity of nanoclay with QAC coatings exhibited more cytotoxic potency than solubilized coating [22, 25]. For THP-1 cells, this suggests that exposure to particle-bound QAC, both extra- and intracellularly, potentially disrupts both cellular plasma and endosome membranes causing robust DAMP release (i.e. IL-1a and ATP), rapid induction of the NLRP3 inflammasome and pyroptosis/necrosis, prior to cleavage of pro-IL-1β taking place. In our in vivo model, both CloisNa and Clois30B induced Caspase 1 cleavage in whole lung lysate at Day 7 post-exposure [17]. In THP-1 cells, higher doses of Clois30B did not elicit a strong IL-1β release, but dose-dependent response curves from cell viability, cell metabolism, membrane damage, and IL-1α release trended well with cathepsin B release and Gasdermin D cleavage suggesting a pyroptotic mode of action. Liu et al. showed that cleaved GSDMD-NT binds to cellular membranes to form pores causing membrane permeability, leading to cell lysis and IL-1β release [51]. In this study, the lack of strong Caspase 1 activity, especially for Clois30B, suggests LPS activation of other caspases, such as non-canonical caspases or Caspase 8, may cleave Gasdermin D to initiate nanoclay-induced pyroptosis [34, 74]. Alternatively, necrotic macrophages release the master alarmin IL-1α which leads to elevated IL-1β secretion in surviving macrophages [75]. The inherent absence of apoptosis contradicts some previous ONC in vitro findings [15], but aligns with several studies in that methyl dihydroxylethyl hydrogenated tallow ammonium and other QAC coatings on nanoclay have minimal apoptotic induction ability [18, 22, 57]. Several different occupational inhalation hazards are known to induce acute lung damage and low initial or delayed inflammation response including Cd/Se-ZnS quantum dots, in part due to slow dissolution rates [76]. Low IL-1β release, but with enhanced IL-1α, ATP, TNFα, IL-6, and IL-8 release at high ONC doses suggests that pyroptosis and necrosis occurs quickly and releases alarmins upon macrophage breakdown [77], but cells are not intact to undergo proper inflammasome-mediated IL-1β release [60, 78]. Alternatively, it has been shown that autophagy may disrupt IL-1β release by targeting ubiquitinated inflammasomes [79]. Low IL-1β release may also partially explain the low and delayed inflammatory response observed in lung tissue in vivo. Slow solubilization of the QAC coating from Clois30B most likely induced a delayed response in vivo resulting in enhanced moderate inflammatory profile with alarmins and low IL-1β at Day 7 [17].

Human THP-1 macrophage release of mixed T_H_1/T_H_2/T_H_17 cytokines following pre-incinerated nanoclay exposure and uptake supports the KE1 secretion of pro-inflammatory cytokines in the lung fibrosis AOP. This matches the in vivo response that included nanoclay uptake by macrophages, mixed T_H_1/T_H_2/T_H_17 inflammatory and pro-fibrotic cytokine profile, and monocyte recruitment following nanoclay deposition in the terminal bronchiole and surrounding alveoli [17]. Silica, aluminum, aluminosilicates, and asbestos are well known to cause acute damage to airway and alveoli [14] with a well-established T_H_1 and T_H_17 inflammatory response [80–82]. Similar acute inflammatory response with elevated IL-1α, IL-1β, TNFα, IL-6, and IL-8 were recently reported in a 3D alveolar model following both nanosilica and crystalline silica [83] and in THP-1 cells following nanosilica exposure [84]. The T_H_2 response (IL-4, IL-5, eotaxin, IL-17α) participates in regulating and maintaining homeostasis during acute phase inflammatory response [85], assists in wound repair of lung lesions [86, 87], and facilitate these particles to act as sensitization adjuvants [88]. This may be a more important factor for ONCs that possess a QAC organic coating since many QACs are known to induce an allergic sensitization response following inhalation or dermal exposure [89, 90]; however, this area requires further research. Interestingly, elevated levels of several T cell chemotaxis, differentiation, and proliferation signals (IL-2, IL-15, IL-7, IL-9) were observed which may partially explain elevated lymphocyte levels at Day 7 following in vivo exposure [17]. However, the role of T cell types, levels, and involvement of T cell-mediated immunological responses during silica pathogenesis [80] are not fully elucidated in nanoclay toxicology and remain a potential area for future research.

Enhanced in vitro secretion of IL-6, GM-CSF, MIP1α/β, MIP-2, and TNFα in exposed THP-1 cells matched in vivo model response at Day 7 and 28 post-exposure. Macrophages typically secrete these factors following silica particulate exposure and play roles in development of adverse lung pathology. For example, prolonged elevated levels of MIP1α and MCP-1 promote chronic lung fibrotic disease [91] while IL-6 is an important driver of bleomycin-induced lung fibrosis in mice [92]. GM-CSF is typically secreted by macrophages, epithelial cells, and fibroblasts in the lung following silica particulate exposure and promotes eosinophil recruitment and stimulates fibroblast collagen production [93, 94]. Collectively, these findings suggest that macrophage exposure to pre-incinerated nanoclay promotes monocyte, eosinophil, and fibroblast chemotaxis and activation that persists in vivo, indicating elevated pro-fibrotic signaling. In both of our in vitro and in vivo models, however, a distinction between how the presence and absence of the organomodifier coating influences inflammation profile and mode of cytotoxicity was clear. Pristine nanoclay (CloisNa) exposure elicited increased TNFα, IFNγ, and IL-10 secretion suggesting an enhanced macrophage chemotaxis and granuloma formation ability compared to Clois30B. Notably, Clois30B only induced TNFα and VEGF secretion in vivo and not in vitro suggesting that another cell type, such as dendritic cells, lymphocytes, or PMNs may secrete these cytokine sources and enhance T_H_1 in vivo response as suggested by previous studies [27, 91, 95, 96].

Comparatively, incineration drastically changed nanoclay particle structure with complete degradation of platelet crystal structure, increased particle size/reduced surface area, and complete loss of the QAC modifier [16] which lead to minimal to moderate reductions in THP-1 live cell counts, apoptosis, and inflammatory potential, with LPS co-exposure increasing sensitivity to cytotoxicity and cell death. The observed morphological and crystal structural differences between I-CloisNa and I-Clois30B moderately impacted cellular responses. The absence of an organic coating allowed for cooling silica oxide to form an amorphous quartz silicon oxide (i.e. I-CloisNa) [97] while presence of the organic coating allowed for some maintenance of a quartz structure (i.e. I-Clois30B) which follows previous findings [98, 99]. Following in vivo exposure, large incinerated particulate with surrounding macrophage was observed in lung tissue while small endocytosed particulate was observed both in tissue and BALF cytospins [17]. Amorphous silica, such as I-CloisNa, possesses high inflammatory potential that is transient in nature with little persistent effect [14, 17]. I-CloisNa clearly displayed elevated inflammatory ability over I-Clois30B at equal high dose in non-LPS stimulated THP-1 cells with elevated levels of TNFα, IL-1β, IL-5, IL-17α, IL-15 and PDGF-ββ which matched in vivo acute transient BALF inflammatory response, increased Caspase 1 cleavage, NF-κB, and Nrf2 expression in lung tissue [17]. I-Clois30B’s mixed morphology of amorphous and platelet-like silica [16, 18] produced a similar inflammatory cytokine profile compared to I-CloisNa, with both highly differing from CS. Interestingly, our finding that I-Clois30B crystal structure matches that of quartz further explains the increase in chronic inflammation in the in vivo model in concordance with the BALF cytokine profile and histopathology of CS-exposed animals. Crystalline silica was previously shown to cause acute mild inflammation and perturb macrophage phagocytosis and apoptosis that results in chronic inflammation and elevated risk of silicosis [14, 100]. The absence of CS effect following acute exposure is possibly due to the absence of reactive silanol groups on aged CS [14] and the retention of some amorphous silica particles within the I-Clois30B sample [16, 17]. Recent increased research into nano-sized, surface coatings, vitreous, and sub-micron amorphous silica largely supports that amorphous surfaces can elicit acute inflammatory responses, but may exhibit reduced toxicity compared to uncoated, crystalline, or highly reactive surface chemistries [14, 101, 102]. The morphological and toxicodynamic similarities of I-Clois30B and CS, in the absence of the QAC coating, indicate the crystalline structure becomes the prevailing factor dictating chronic inflammatory response. This is juxtaposed to I-CloisNa with a pyrogenic amorphous structure and acute, but transient, inflammatory effect. This indicates that prolonged incinerated ONC exposure represents a potential risk to long-term pulmonary health.

In vitro to in vivo correlation analyses revealed that in vitro model for human alveolar macrophage for cell viability, IL-1β release, and gasdermin D cleavage moderately to strongly aligned with in vivo BAL monocyte, neutrophil, and lymphocyte cell infiltrate counts on either Day 1 or Day 7 post-exposure. Furthermore, it supports the role of macrophages in the KE1 release of pro-inflammatory mediators driving KE2 increased recruitment of pro-inflammatory cells in the proposed lung fibrosis AOP. Some of the stronger correlations occurred with 3- or 10-fold increase in in vitro dose above the in vivo equivalent mass per area dose. This is not surprising since in vivo mass dose assumes that particles deposit entirely in the respiratory bronchi/alveoli and homogeneously across the alveolar surface in the lung while assuming macrophages are not mobile. Since macrophages are mobile with a primary function to remove foreign material, it is expected that intracellular mass dose will be well above the local deposited mass dose. Next, non-LPS exposed macrophage cytokine profiles correlated with in vivo BAL profile (while LPS-stimulated macrophages did not) as both in vitro and in vivo hazard characterization studies are conducted in pathogen-free conditions with sterile particles. This analysis identified a five-cytokine profile (MIP1α, MIP1β, IL-8, TNFα, IL-6) for ONC that could be used in future nanoclay in vitro toxicity studies to screen for inflammatory effects. A previous ONC study also found in vitro macrophage IL-8 expression correlated with their in vivo findings [26]. Lastly, several cytokines involved in T_H_2 cell recruitment and fibrosis (IL-6, eotaxin, PDGF-ββ, IL-10, IL-1β) were released from macrophages in vitro and correlated with in vivo BAL response, which aligns with KE4 (T_H_2 cell activation) in the lung fibrosis AOP. Collectively, this suggests in vitro macrophage response assessment as an appropriate in vitro screening tool for ONC lung toxicology research.

Given the diversity of different morphologies and chemistries along nanoclay life cycles, traditional in vivo toxicity testing is not feasible. Here, we used a tiered screening strategy evaluating relationships between particle physicochemical properties and biological effect. Limitations to the study include uncertainty in deposited dose based on differences in effective density and heterogeneity in particle suspensions and how in vitro doses relate to unknown in vivo deposited doses in the deep lung following known administered dose. Improvements to this approach can be developed to rapidly identify those nanoclays that pose elevated risk in occupational settings during handling and disposal processes [50, 103, 104].

## Conclusions

Herein, we evaluated two types of montmorillonite nanoclay and their incinerated byproducts for their toxicological and immunological profiles. We found clear evidence that physicochemical differences, namely presence/absence of QAC coating and incineration status, of nanoclays determines key events and molecular signaling patterns associated with lung macrophage inflammatory and cytotoxic responses. Further, we found correlations between toxicological and immunological effects observed in vitro to those observed in a mouse model, adding to our understanding of ONC’s toxic mechanism along its life cycle and demonstrating the suitability of the in vitro non-LPS primed differentiated THP-1 model as a screening tool for nanoclays along their lifecycle.

### Supplementary Information

**Additional file 1: Figures**. 1–12 documenting particle characterization, uptake, high-throughput imaging, and protein expression data.

**Additional file 2**: Methods for fluorescent high content imaging of macrophage differentiation, differentiation results, and detailed physicochemical analysis results.

**Additional file 3: Tables×** S1–10 containing particle characterization, secreted cytokine levels, and correlation coefficients.

## Data Availability

A majority of the data generated or analyzed during this study are included in this published article [and its Supplemental information files]. Additional datasets used during the current study and the code block for generating ED_50_ curves are available from the corresponding author on reasonable request or can be found on the NIOSH Data and Statistics Gateway.

## Abbreviations

AOP: Adverse outcome pathway
CloisNa: Cloisite^®^ Na, uncoated montmorillonite
Clois30B: Cloisite^®^ 30B, organomodified montmorillonite
I-CloisNa: Incinerated Cloisite^®^ Na^+^
I-Clois30B: Incinerated Cloisite^®^ 30B
CS: Heat-inactivated crystalline silica
DLS: Dynamic light scattering
ELISA: Enzyme-linked immunosorbent assay
FESEM: Field emission scanning electron microscopy
GSDMD: Gasdermin D
GSDMD-NT: Gasdermin D N-terminus
HTS: High-throughput screening
LDH: Lactate dehydrogenase
LPS: Lipopolysaccharide
MMP: Mitochondrial membrane potential
ONC: Organomodified nanoclay
PdI: Particle dispersity index
QAC: Quaternary ammonium compound
RPMI: Roswell Park Memorial Institute
TEM: Transmission electron microscopy
THP-1: Human monocytic cells
WST-1: Water soluble tetrazolium salt—1
XRD: X-ray diffraction
