# Supplementary material for: In vitro inflammation and toxicity assessment of pre- and post-incinerated organomodified nanoclays to macrophages using high-throughput screening approaches
Source: Part Fibre Toxicol. 2024 Mar 21;21:16. doi: 10.1186/s12989-024-00577-7 (PMC10956245; doi:10.1186/s12989-024-00577-7)
Supplement: Supplementary file 1 — Additional file 1: Figures. 1–12 documenting particle characterization, uptake, high-throughput imaging, and protein expression data. [file 12989_2024_577_MOESM1_ESM.pdf]

Supplementary Figures

In vitro inflammation and toxicity assessment of pre- and post-incinerated organomodified nanoclays to macrophages using high-throughput screening approaches

Todd A. Stueckle[1*], Jake Jensen[1], Jayme Coyle[1], Raymond Derk[1], Alixandra Wagner[2], Cerasela Zoica Dinu[2], Tiffany G. Kornberg[1], Sherri A. Friend[1], Alan Dozier[1], Sushant Agarwal[2], Rakesh K. Gupta[2], Liying W. Rojanasakul[1]

[1] *Health Effects Laboratory Division, National Institute for Occupational Safety and Health, Morgantown, WV*

[2] *Biomedical and Chemical Engineering, West Virginia University, Morgantown, WV*

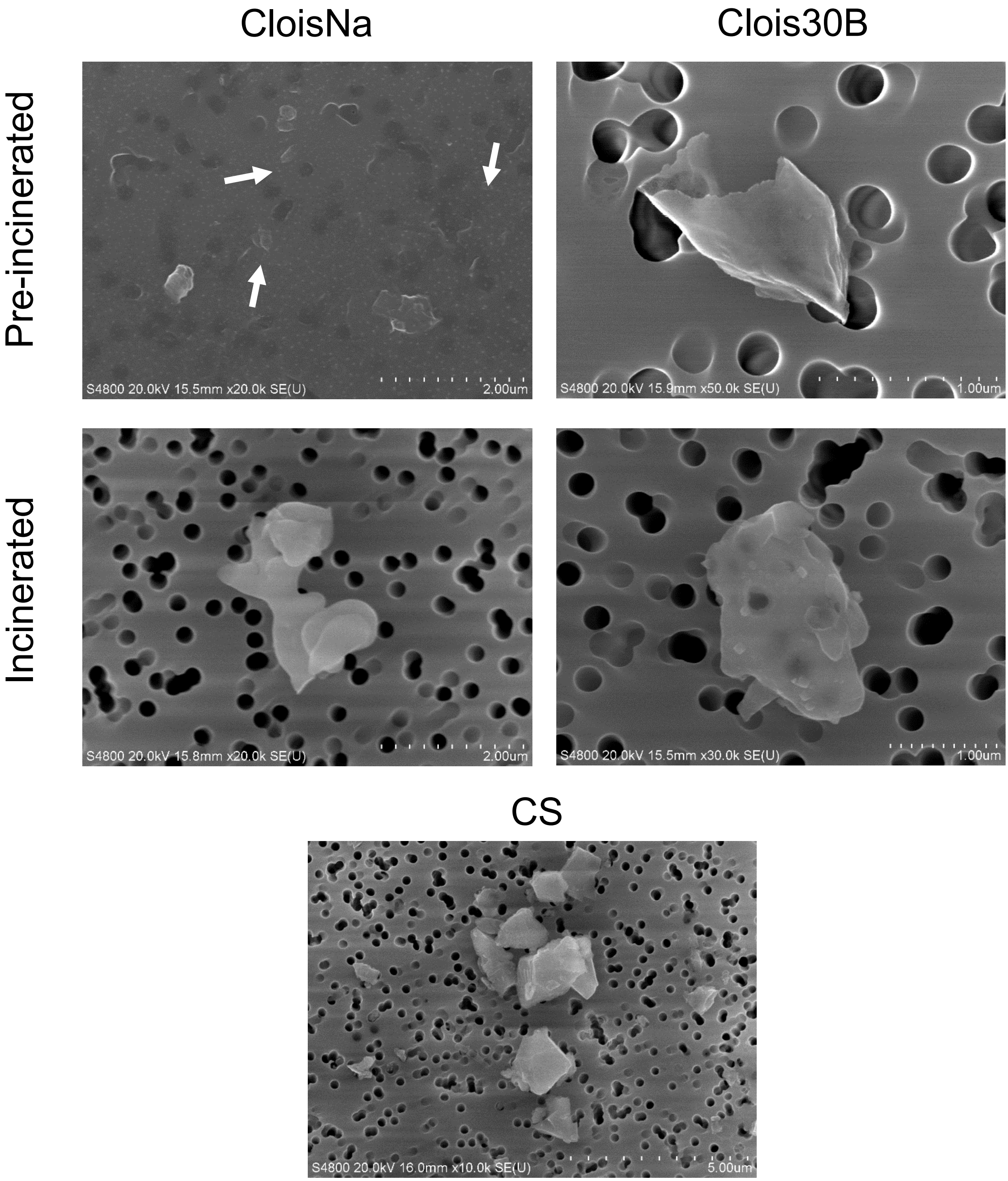

Supplementary Figure 1. Dispersion and morphology of pre- and post-incinerated organomodified nanoclays in RPMI + 10% FBS medium. Particles at 1 mg/ml in water were sonicated and diluted in cell culture medium to 10 µg/ml, filtered, and prepared for FESEM imaging. White arrows indicate well-dispersed single platelets.

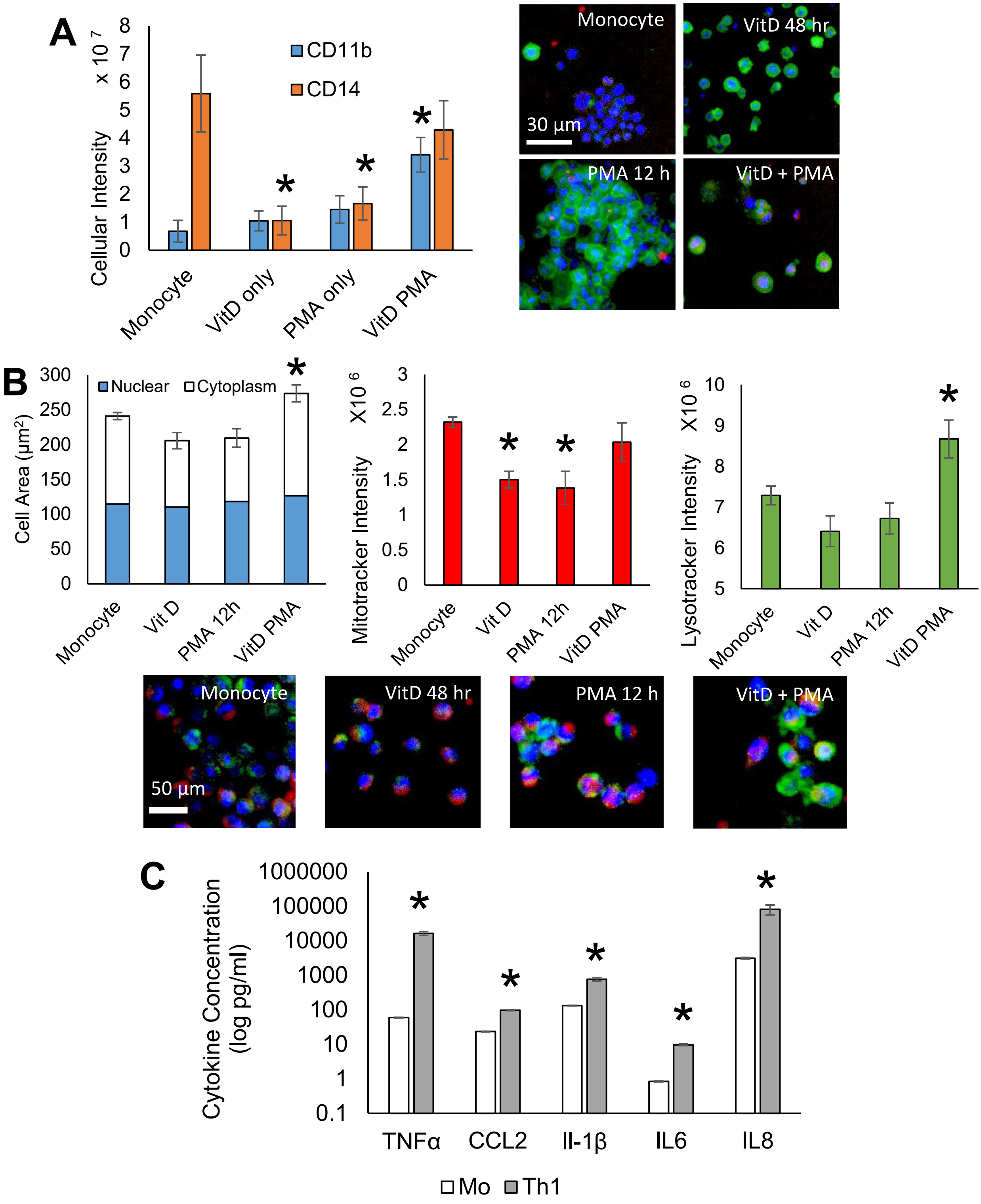

Supplementary Figure 2. Characterization of THP-1 cells following Mo and Th1 differentiation. Cells were undifferentiated monocytes, vitamin D for 48 hr, PMA for 12 hours, or Vit D + PMA for 48 hr. A) CD11b (green) and CD14 (red) cellular signal intensity. B) Cell area, Mitotracker Red, and Lysotracker Green cellular intensity. C) Th1-associated cytokine expression in non-particle-exposed cells in PMA differentiated cells (Mo) and LPS-stimulated cells (Th1). Data represent means ±SE (n=3). * are those treatments significantly different from monocytes (Mo; p ≤ 0.05).

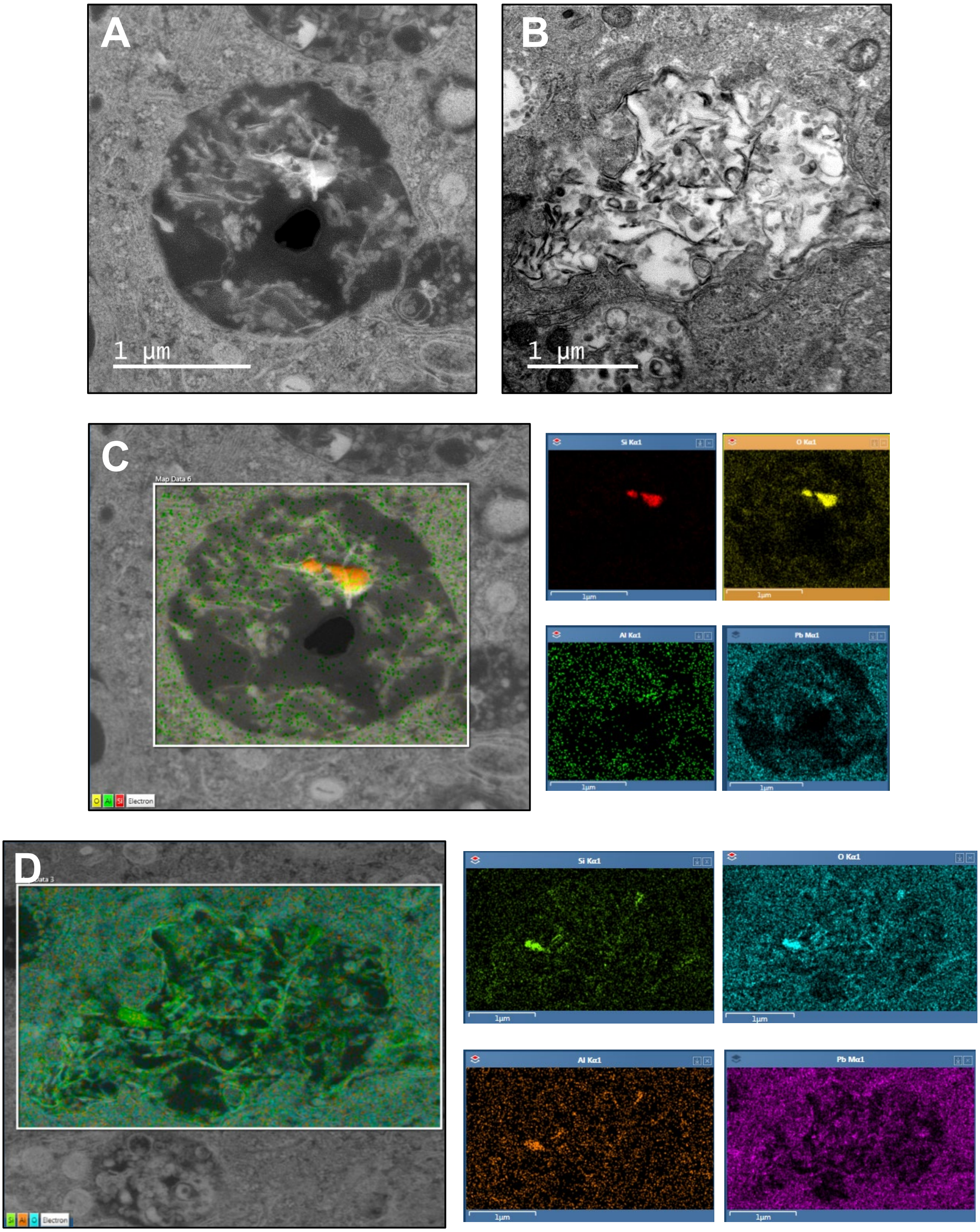

Supplementary Figure 3. Elemental mapping analysis of CloisNa particles in differentiated THP-1 macrophages. A and B) Transmission electron microscopy localization of CloisNa within endosomes. C and D) Energy dispersive x-ray spectroscopy mapping of positive co-localization of silicon (red C, green D), oxygen (yellow C, blue D) and aluminum (green C, orange D) along with particle morphology confirmed CloisNa. Nanoclay platelets perpendicular to the camera are observable in B and D. Sections were stained with lead acetate (purple).

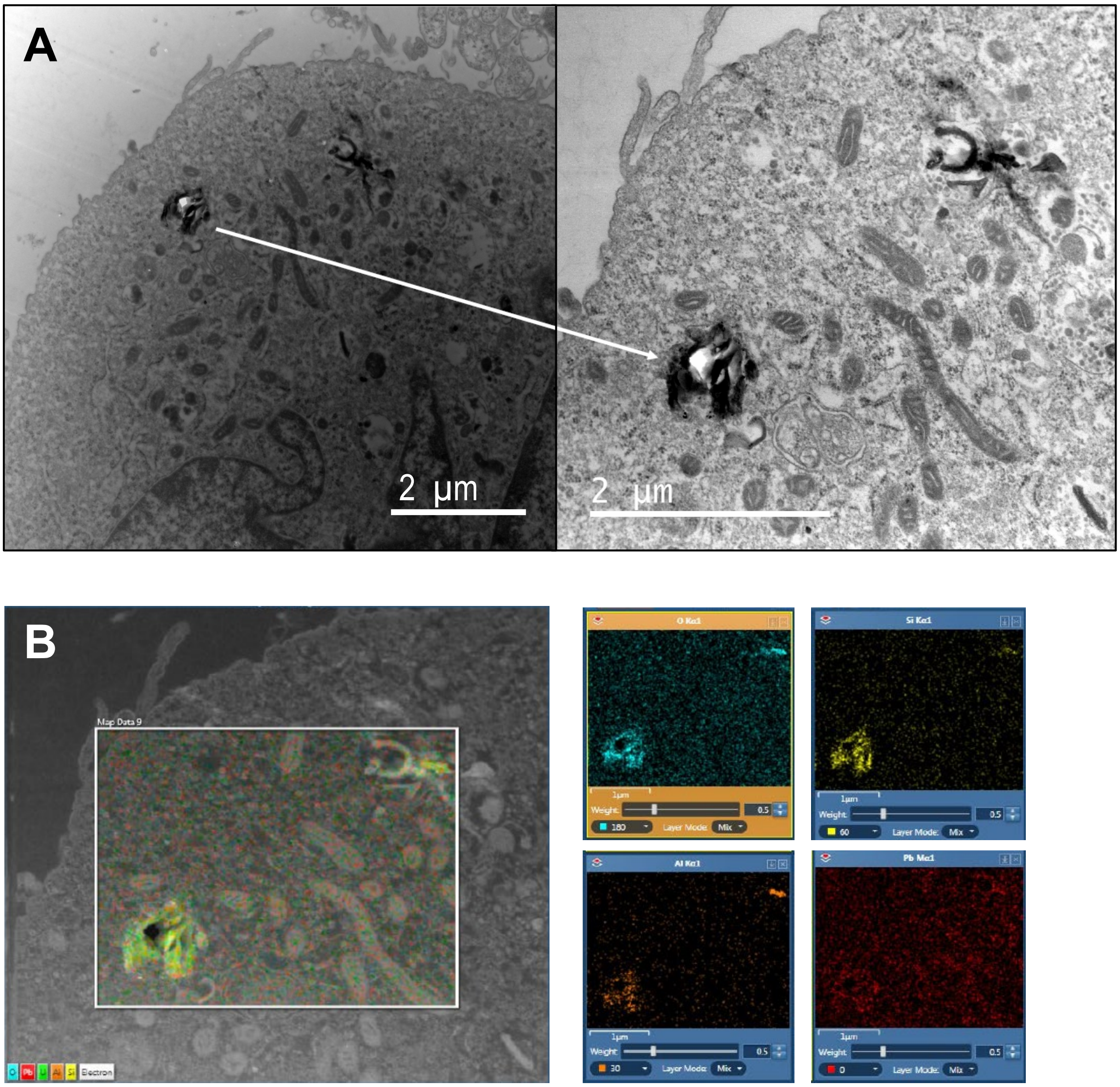

Supplementary Figure 4. Elemental mapping analysis of Clois30B particles in differentiated THP-1 macrophages. A) Transmission electron microscopy localization of Clois30B. B) Energy dispersive x-ray spectroscopy mapping of positive co-localization of silicon (yellow), oxygen (blue) and aluminum (orange) along with particle morphology confirmed Clois30B. Sections were stained with lead acetate (red).

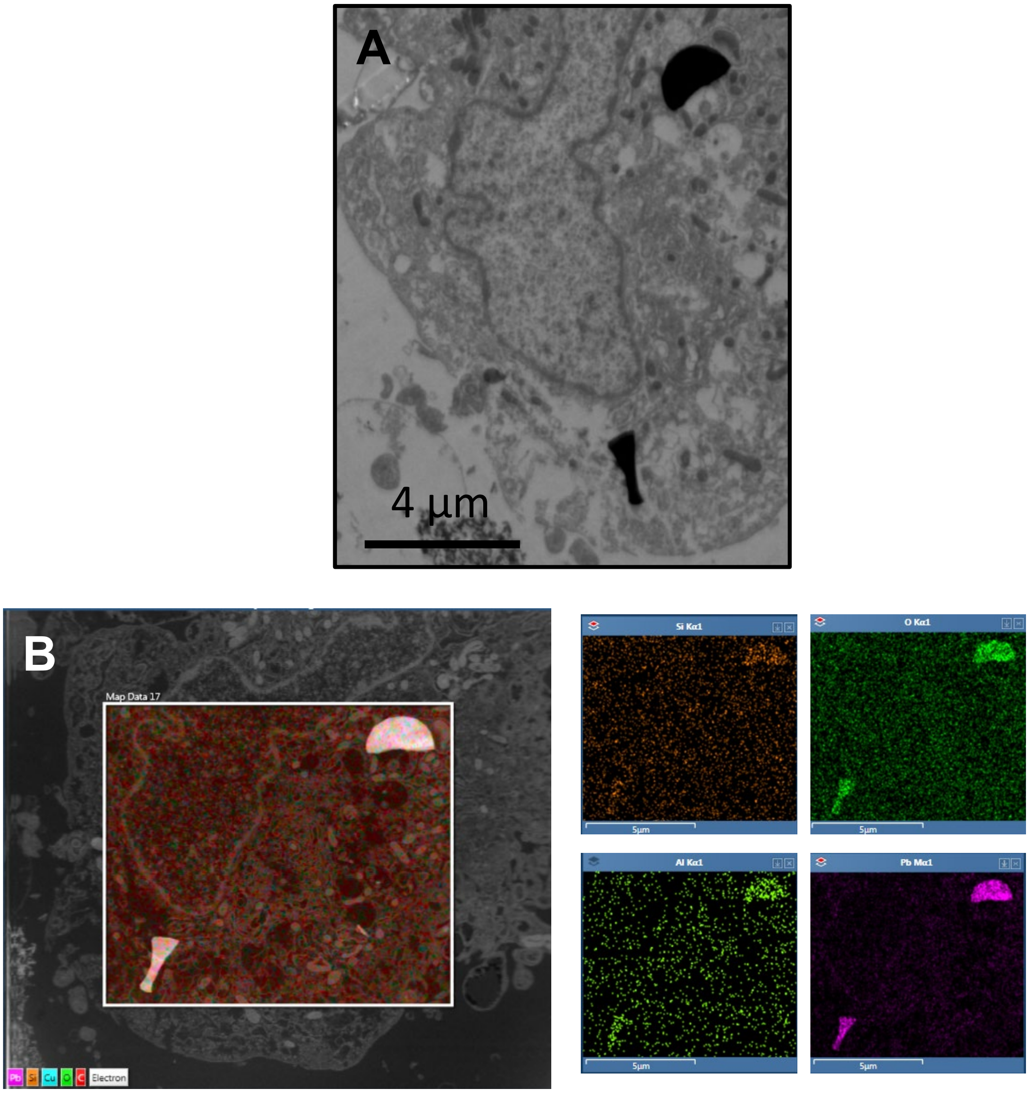

Supplementary Figure 5. Elemental mapping analysis of I-CloisNa particles in differentiated THP-1 macrophages. A) Transmission electron microscopy localization of I-CloisNa. B) Energy dispersive x-ray spectroscopy mapping of positive co-localization of silicon (orange), oxygen (green) and aluminum (chartreuse) along with particle morphology confirmed I-CloisNa. Sections were stained with lead acetate (purple).

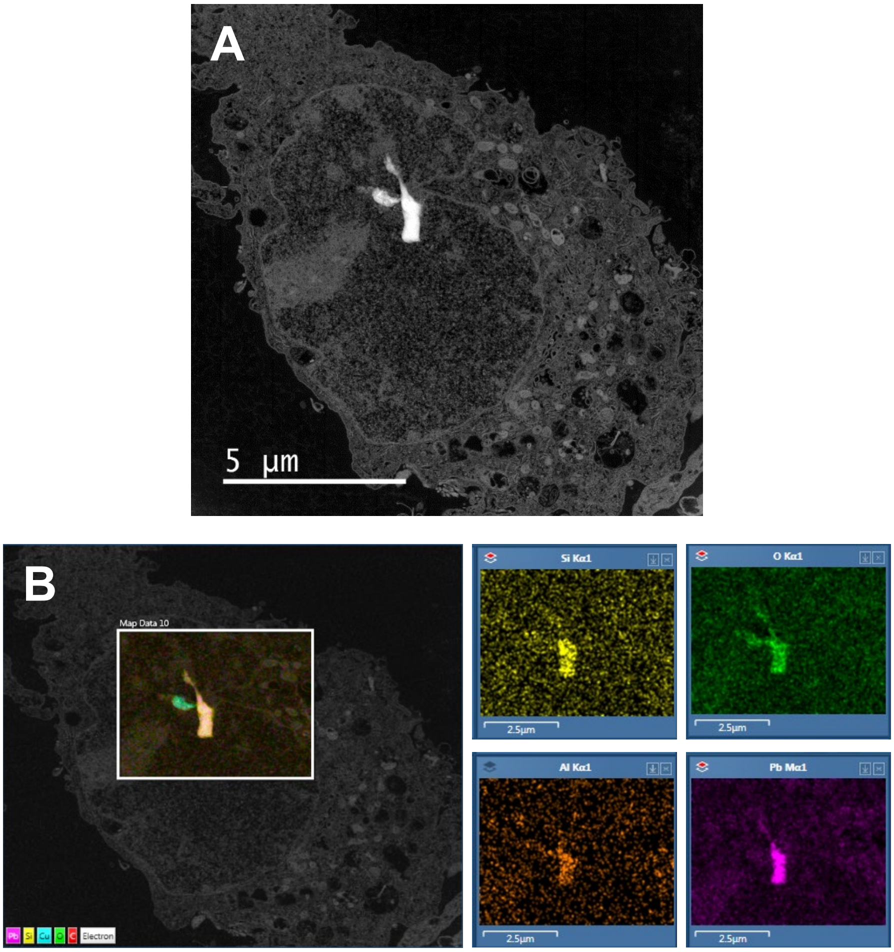

Supplementary Figure 6. Elemental mapping analysis of I-Clois30B particle in differentiated THP-1 macrophages. A) Transmission electron microscopy localization of I-Clois30B. B) Energy dispersive x-ray spectroscopy mapping of positive co-localization of silicon (yellow), oxygen (green) and aluminum (orange) along with particle morphology confirmed I-Clois30B. Sections were stained with lead acetate (purple).

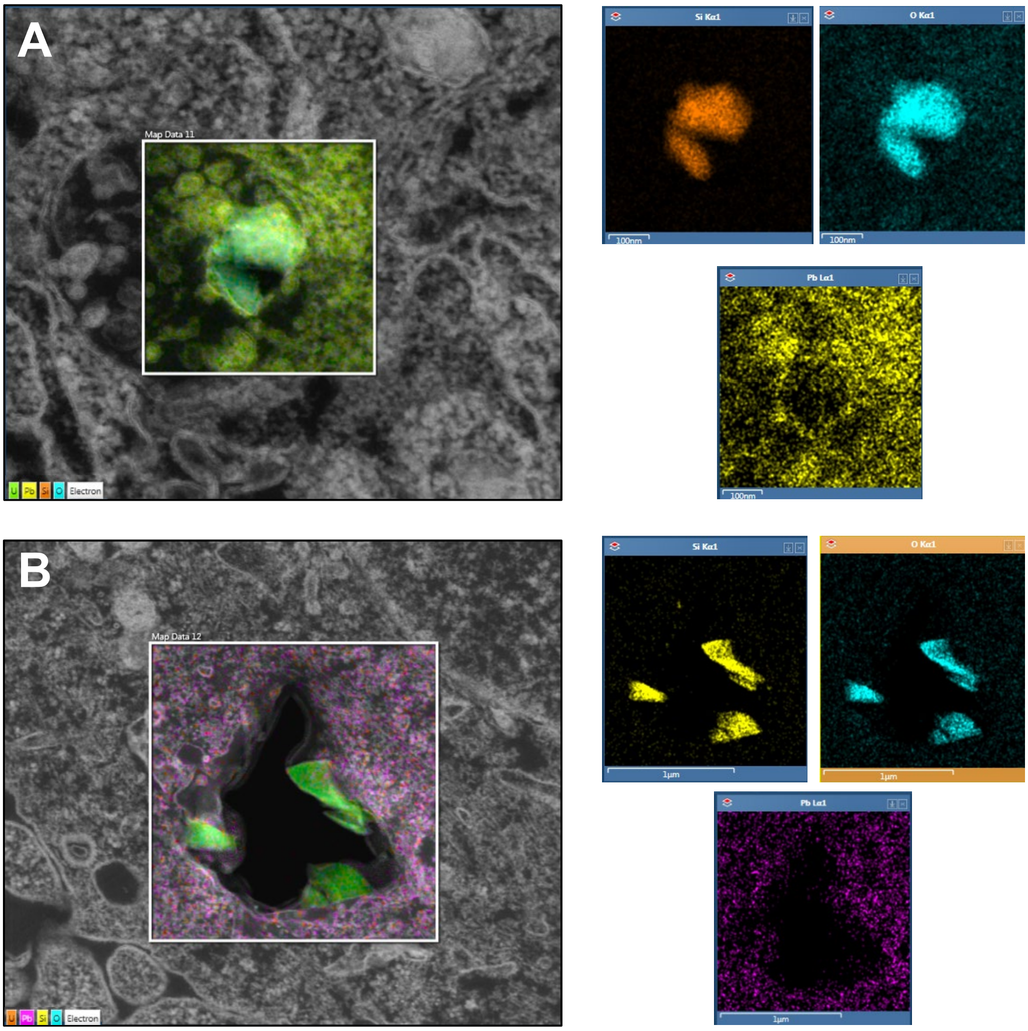

Supplementary Figure 7. Elemental mapping analysis of crystalline silica (CS) particles in differentiated THP-1 macrophages. A) Transmission electron microscopy localization of CS (left). Energy dispersive x-ray spectroscopy mapping of positive co-localization of silicon (orange), oxygen (blue) and particle morphology confirmed CS. B) Transmission electron microscopy localization of several CS in an endosome (left). Energy dispersive x-ray spectroscopy mapping of positive co-localization of silicon (yellow), oxygen (blue) and particle morphology confirmed CS. Sections were stained with lead acetate (yellow and purple, respectively).

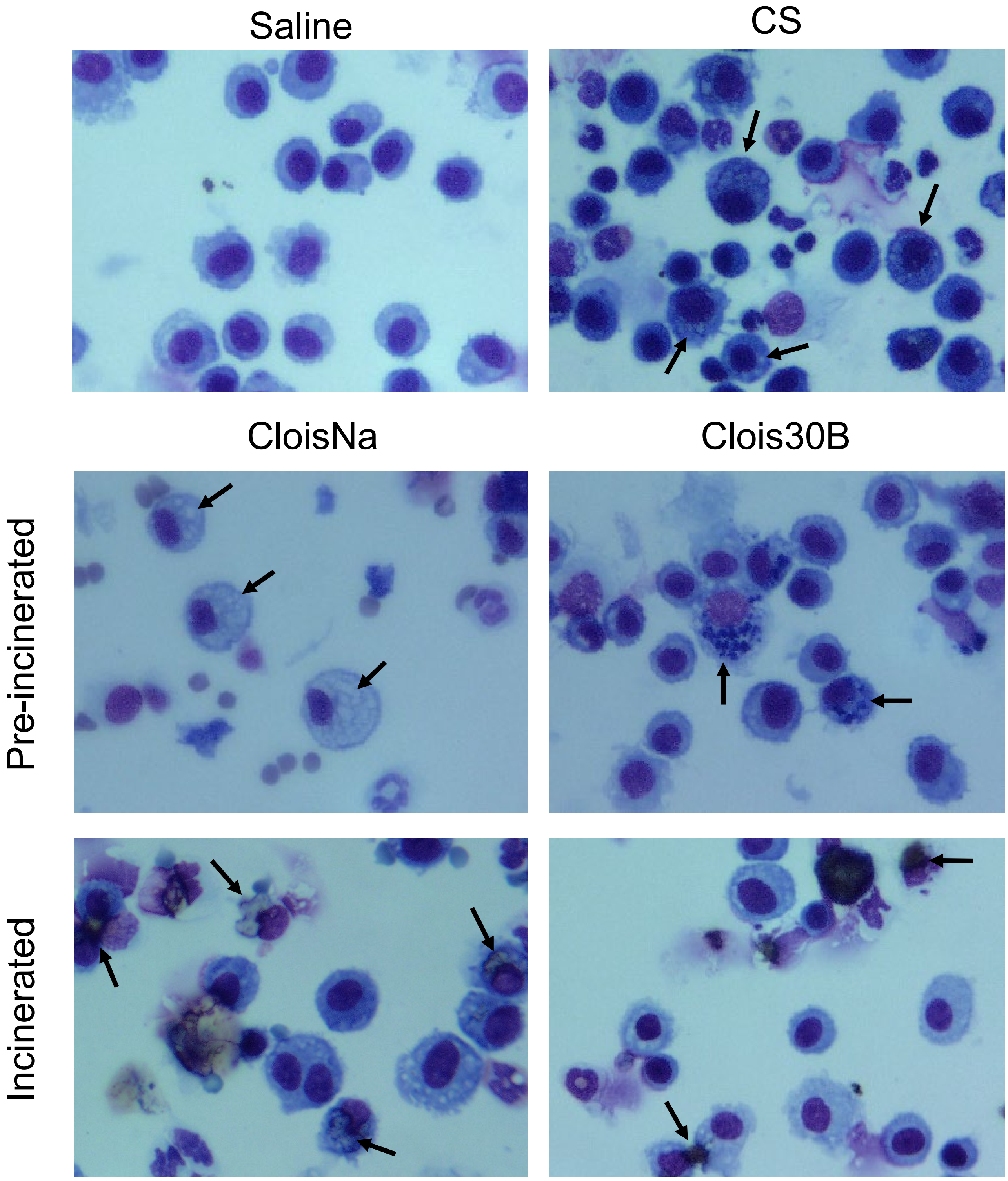

Supplementary Figure 8. Bronchioalveolar lavage cells at Day 1 post-aspiration of pre- and post-incinerated organomodified nanoclays in male C57BL/6J mice. BALF samples were centrifuged on a Cytospin and HEMA3-fixed and stained following collection. All particle exposures elicited active macrophages and neutrophils within collected BALF samples. Arrows indicate macrophages with engulfed particulate. Images captured with 40X objective.

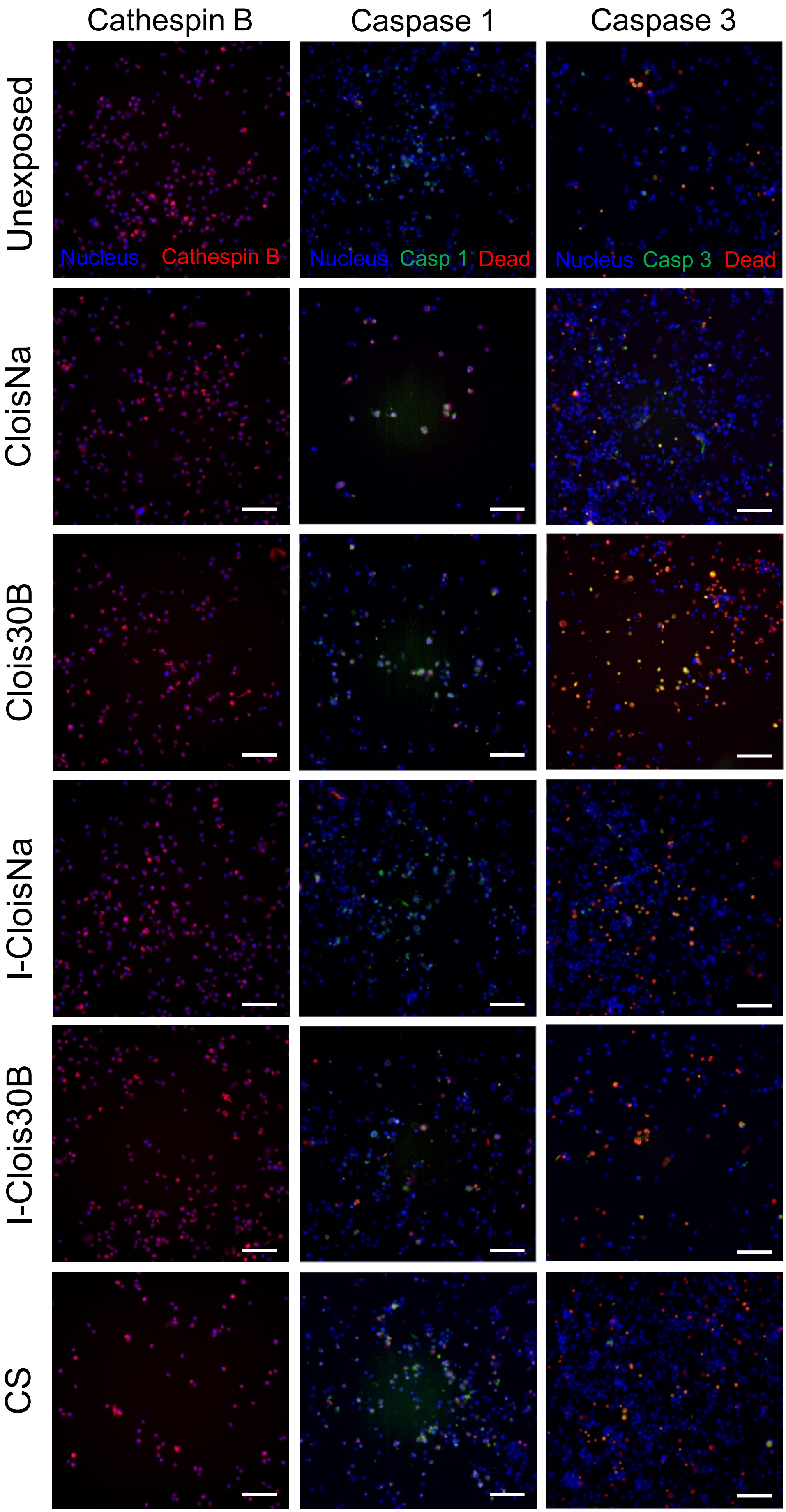
Cathespin B
Caspase 1
Caspase 3
Unexposed
CloisNa
Clois30B
I-CloisNa
I-Clois30B
CS
Nucleus
Cathespin B
Nucleus
Casp 1
Dead
Nucleus
Casp 3
Dead

Supplementary Figure 9 (previous page). Representative images of a HCI screening panel for pyroptosis, apoptosis, and necrosis of nanoclay-exposed (2.0 μg/cm^2) differentiated THP-1 cells. Differentiated cells were exposed (0 – 20 μg/cm^2) for 6 hrs to pre- and post-incinerated nanoclays followed by multiplex staining and high-throughput imaging. Crystalline silica (CS) was included as a legacy particle control. Data are quantitated in Figure 4. Scare bars represent 200 μm.

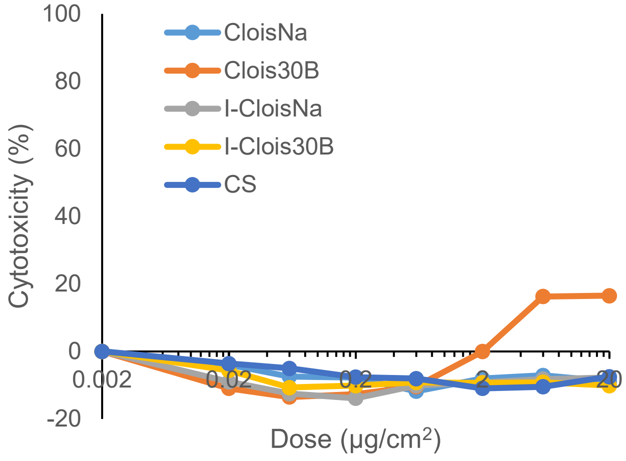

Supplementary Figure 10. Differentiated THP-1 cell cytotoxicity measured by LDH assay in the presence of LPS.

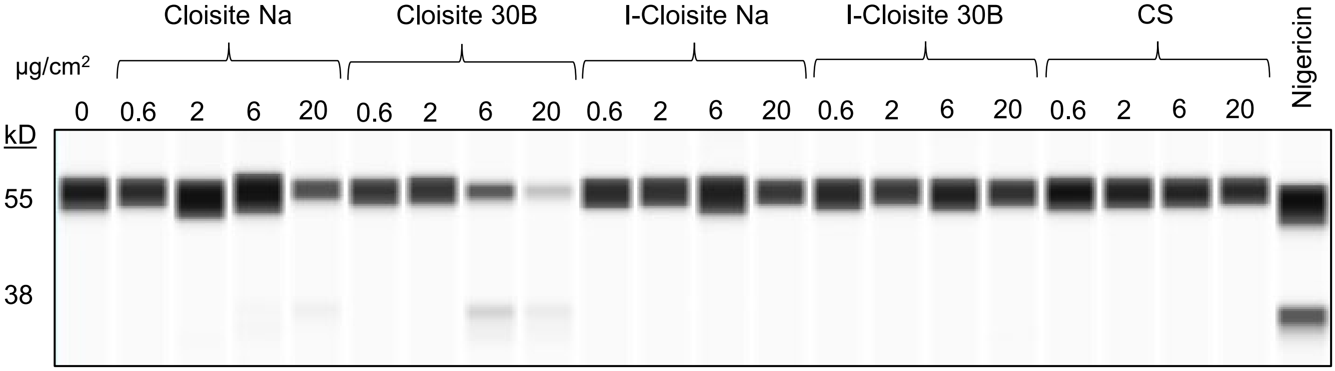

Supplementary Figure 11. Gasdermin D (55 kD) and N-terminus cleavage product(38 kD) expression in differentiated THP-1 cells co-stimulated with LPS following exposure to pre- and post-incinerated organomodified nanoclay for 24 h. Nigericin-exposed cells served as a positive pyroptosis control.

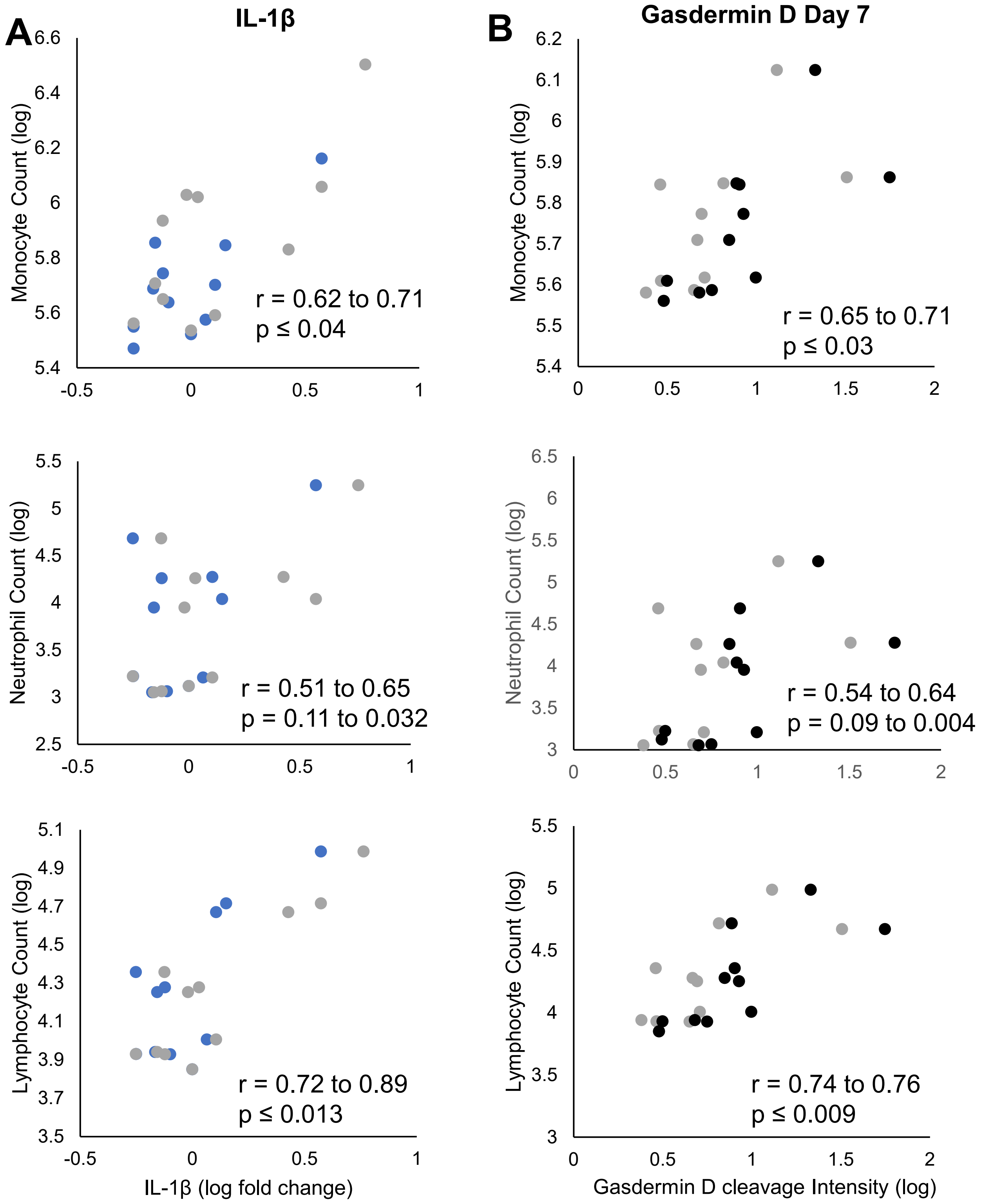

Supplementary Figure 12. Representative plots of *in vitro* THP-1 macrophage IL-1β and gasdermin D cleavage response significantly correlated to male C57Bl/6J mouse BAL monocyte, neutrophil, and lymphocyte counts at 30 and 300 μg/lung of pre- and post-incinerated nanoclay exposure. A) IL-1 β levels at Day 1 (top) and Day 7 (middle and lower) post-exposure. B) Gasdermin D cleavage levels correlated to Day 7 cell differentials. Blue, grey, and black dots represent 0.06 and 0.6 μg/cm^2, 0.6 and 6 μg/cm^2, and 2 and 20 μg/cm^2 respectively.
