# Supplementary material for: In vitro inflammation and toxicity assessment of pre- and post-incinerated organomodified nanoclays to macrophages using high-throughput screening approaches
Source: Part Fibre Toxicol. 2024 Mar 21;21:16. doi: 10.1186/s12989-024-00577-7 (PMC10956245; doi:10.1186/s12989-024-00577-7)
Supplement: Supplementary file 2 — Additional file 2: Methods for fluorescent high content imaging of macrophage differentiation, differentiation results, and detailed physicochemical analysis results. [file 12989_2024_577_MOESM2_ESM.docx]

**Supplementary Methods and Results**

***In vitro* inflammation and toxicity assessment of pre- and post-incinerated organomodified nanoclays to macrophages using high-throughput screening approaches**

Todd A. Stueckle^1^*, Jake Jensen^1^, Jayme Coyle^1^, Raymond Derk^1^, Alixandra Wagner^2^, Cerasela Zoica Dinu^2^, Tiffany G. Kornberg^1^, Sherri A. Friend^1^, Alan Dozier^1^, Sushant Agarwal^2^, Rakesh K. Gupta^2^, Liying W. Rojanasakul^1^

*^1^ Health Effects Laboratory Division, National Institute for Occupational Safety and Health, Morgantown, WV*

*^2^ Biomedical and Chemical Engineering, West Virginia University, Morgantown, WV*

**Supplementary Methods**

***THP-1 macrophage differentiation confirmation analyses***

Confirmation of THP-1 cells from suspended monocytes to differentiated, attached macrophages with specific markers using a combined treatment of 1α, 25-dihydroxy-Vitamin D3 (VitD) and phorbol, 12-myristate, 13-acetate (PMA) was conducted. First, human THP-1 monocytes were plated in quadruplicate at 2 x 10^4^ cells in black-walled clear bottom tissue culture-treated 96 well plates. Monocytes were exposed to one of four treatments which included normal growth medium for 48 hours, 150 nM VitD for 48 hours, normal growth medium for 36 hours followed by 10 nM PMA for the last 12 hours, or 150 nM for 36 hours with 10 nM PMA for the last 12 hours. Single compound exposures were performed to ascertain their effect on macrophage differentiation.

Cells were prepared for immunofluorescent staining and high content imaging following a previously published protocol(1). Briefly, following exposure all treatments (except monocytes) were washed with sterile-filtered phosphate-buffered saline (PBS) to remove unattached cells. Next, cells were fixed with 4% paraformaldehyde for 15 minutes and permeabilized with 0.025% Triton-X in PBS for 15 minutes. Using a multi-channel pipette cells were triple rinsed in PBS and blocked using 1% bovine serum albumin in PBS (Themofisher Scientific). Next, 1:200 dilution of mouse monoclonal human CD14 antibody (eBioscience) and 1:400 dilution of rabbit monoclonal human CD11b antibody were incubated with cells for 1 hour at room temperature. Cells were triple-rinsed and incubated with 1 µM Hoescht 33342, 1:600 dilutions of secondary goat anti-mouse (AlexaFluor 647) and goat anti-rabbit (AlexaFluor 488) antibodies (Thermofisher Scientific) for 1 hour at room temperature. Cells were triple-rinsed and held in PBS for high content imaging. Nine sites per well were imaged at 20X magnification using DAPI, FITC, and Cy5 channels on the ImageXpress Micro XLS system. Cellular integrated intensity was quantified for each cell, averaged per site, and averaged across technical replicates in MetaXpress. Summary statistics including means and standard error were calculated with data from three independent experiments.

To ascertain how differentiation protocol influenced the amount of lysosomes and mitochondria, live cell staining and imaging were conducted. Cells were seeded and exposed to treatments as described above. Next, the plate was centrifuged at 125 x g for 5 minutes to retain suspended monocytes. Medium was carefully aspirated and live cells were stained with 50 nM Lysotracker Green DND-26, 200 nM Mitotracker Red C2XRos, and 1 µM Hoescht 33342 in warm PBS in the incubator for 45 minutes following manufacturer’s protocols. Staining solution was carefully aspirated followed by the addition of 100 µL of fresh growth medium. The plate was again centrifuged and immediately imaged under culture conditions (humid 5% CO_2_ air) using the Environmental Control module (Molecular Devices). Nine sites per well were imaged at 20X magnification using DAPI, FITC, and Texas Red channels on the ImageXpress Micro XLS system. Cell area, nuclear area, and cellular integrated intensity for each channel was quantified for each cell, averaged per site, and averaged across technical replicates in MetaXpress. Summary statistics including means and standard error were calculated with data from three independent experiments. Differences among treatment groups was performed using a one-way ANOVA and post-hoc tests described in the Methods.

Lastly, multiplex cytokine data from the Luminex array was used to show the major baseline differences of several inflammatory cytokines in non-LPS exposed naïve macrophages (M_o_) and LPS-exposed T_H_1 macrophages. Means, error estimates, and associated statistical outcomes were plotted.

**RESULTS**

***Basal spacing and crystal structure characterization by XRD***

All particle characteristics in dry and culture medium suspension are summarized in Table 2. Basal spacing and material structure of the nanoclays was determined via XRD in the 2θ range of 2-9.5° and 5-100°, respectively. Similar to previous results, pre-incinerated CloisNa and Clois30B had basal spacing’s of 1.21 nm (indicated by a 2θ peak around 7.3°) and 1.85 nm (indicated by peak a 2θ around 4.8°), respectively (Figure 1A). Neither of the incinerated forms of the nanoclays displayed peaks in the 2-9.5° 2θ range. CloisNa displayed a pattern strongly resembling that of montmorillonite (RRUFF database: Montmorillonite R110052) (2-7) with dioctahedral structure (MMT; Figure 1B), with 2θ peaks at 6.4, 20.1, 26.9, 35.2, 54.4, 62.2, 73.3 and 76.6 °. Clois30B displayed a similar pattern relative to CloisNa (and MMT) though some of the 2θ peak positions were shifted a few tenths of a degree in the 2θ range to the left toward smaller angles (8, 9). The XRD pattern of I-CloisNa displayed reflections representative of amorphous quartz silicon oxide (X’Pert HighScore: 98-015-5251) (10, 11) indicated by 2θ peaks at 21.9, 35.9, and 65.9°. Finally, I-Clois30B strongly resembled that of quartz reference pattern (X’Pert HighScore: 98-008-9289) (6, 12-14) with 2θ peaks of 20.2, 26.2, 35.6, 39.5, 49.3, and 67.2°. Collectively, these data indicated clear differences in both platelet spacing and material structure related to the presence of organic coating and incineration status.

***Vitamin D with low dose PMA differentiate THP-1 monocytes into attached macrophages***

THP-1 monocytes in culture exhibited high CD14 expression, low CD11b expression, and clumped morphology in suspension (Supplementary Figure 2A). Sole addition of either VitD for 48 h or PMA for 12 h caused a substantial significant 5.1-fold drop in CD14 expression, no change in CD11b expression, and attached cells compared to monocytes. Notably, VitD exposure reduced the amount of cell clumping compared to monocytes and PMA-exposed cells. Co-exposure to VitD followed by PMA caused a significant 5-fold increase in CD11b expression, a return of CD14 expression comparable to THP-1 monocytes, and reduced cell clumping.

Next, sole addition of either VitD or PMA caused no significant change in cell area, lysosome intensity, and a significant decrease in mitochondria intensity compared to monocytes (Supplementary Figure 2B). Co-exposure to VitD and PMA resulted in increased cell area, and significant increase in lysosome signal intensity. Mitochondria intensity did not differ compared to monocytes. Collectively, these results show that VitD followed by low dose PMA exposure resulted in attached CD11b-positive macrophages with greater number of lysosomes and cytoplasm than THP-1 monocytes.

**References**

1. Coyle JP, Johnson C, Jensen J, Farcas M, Derk R, Stueckle TA, et al. Variation in pentose phosphate pathway-associated metabolism dictates cytotoxicity outcomes determined by tetrazolium reduction assays. Sci Rep. 2023;13(1):8220.

2. Veiskarami M, Sarvi MN, Mokhtari AR. Influence of the purity of montmorillonite on its surface modification with an alkyl-ammonium salt. Applied Clay Science. 2016;120:111-20.

3. Fatimah I, Huda T. Indonesian Montmorillonite-supported ZnO: Preparation, Characterization and Activity Test in Methanol Dehydration. Asian Journal of Materials Science. 2012;4(1):13-20.

4. He Y, Jiang B, Chen J, Jiang Y, Zhang YX. Synthesis of MnO2 nanosheets on montmorillonite for oxidative degradation and adsorption of methylene blue. Journal of colloid and interface science. 2018;510:207-20.

5. Yu S, Mei H, Chen X, Tan X, Ahmad B, Alsaedi A, et al. Impact of environmental conditions on the sorption behavior of radionuclide 90Sr(II) on Na-montmorillonite. Journal of Molecular Liquids. 2015;203:39-46.

6. Bieseki L, Bertell F, Treichel H, Penha FG, Pergher SBC. Acid treatments of montmorillonite-rich clay for Fe removal using a factorial design method. Materials Research. 2013;16(5):1122-7.

7. Wu H, Xie H, He G, Guan Y, Zhang Y. Effects of the pH and anions on the adsorption of tetracycline on iron-montmorillonite. Applied Clay Science. 2016;119:161-9.

8. Romanzini D, Piroli V, Frache A, Zattera AJ, Amico SC. Sodium montmorillonite modified with methacryloxy and vinylsilanes: Influence of silylation on the morphology of clay/unsaturated polyester nanocomposites. Applied Clay Science. 2015;114:550-7.

9. Charlon S, Marais S, Dargent E, Soulestin J, Sclavons M, Follain N. Structure–barrier property relationship of biodegradable poly(butylene succinate) and poly[(butylene succinate)-co-(butylene adipate)] nanocomposites: influence of the rigid amorphous fraction. Physical Chemistry Chemical Physics. 2015;17:29918-34.

10. Pouya ES, Abolghasemi H, Fatoorehchi H, Rasem B, Hashemi SJ. Effect of dispersed hydrophilic silicon dioxide nanoparticles on batch adsorption of benzoic acid from aqueous solution using modified natural vermiculite: An equilibrium study. Journal of Applied Research and Technology. 2016;14(5):325-37.

11. Ullah R, Deb BK, Mollah MYA. Synthesis and Characterization of Silica Coated Iron-Oxide Composites of Different Ratios. International Journal of Composite Materials. 2014;4(2):135-45.

12. Munasir M, Triwikantoro T, Zainuri M, Darminto D. Synthesis of SiO2 nanopowders containing quartz and cristobalite phases from silica sands. Materials Science-Poland. 2015;33(1):47-55.

13. Saceda JF, de Leon RL, Rintramee K, Prayoonpokarach S, Wittayakun J. Properties of Silica from Rice Husk and Rice Husk Ash and their Utilization for Zeiolite Y Synthesis Quim Nova. 2011;34:1394-7.

14. Sanz J, Madani A, Serratosa JM, Moya JS, Aza S. Aluminum-27 and Silicon-29 Magic-Angle Spinning Nuclear Magnetic Resonance Study of the Kaolinite-Mullite Transformation. Communications of the American Ceramic Society. 1988;71:C-418-C-21.
