# Supplementary material for: In vitro inflammation and toxicity assessment of pre- and post-incinerated organomodified nanoclays to macrophages using high-throughput screening approaches
Source: Part Fibre Toxicol. 2024 Mar 21;21:16. doi: 10.1186/s12989-024-00577-7 (PMC10956245; doi:10.1186/s12989-024-00577-7)
Supplement: Supplementary file 3 — Additional file 3: Tables× S1–10 containing particle characterization, secreted cytokine levels, and correlation coefficients. [file 12989_2024_577_MOESM3_ESM.docx]

**Supplementary Tables**

***In vitro* inflammation and toxicity assessment of pre- and post-incinerated organomodified nanoclays to macrophages using high-throughput screening approaches**

Todd A. Stueckle^1^*, Jake Jensen^1^, Jayme Coyle^1^, Raymond Derk^1^, Alixandra Wagner^2^, Cerasela Zoica Dinu^2^, Tiffany G. Kornberg^1^, Sherri A. Friend^1^, Alan Dozier^1^, Sushant Agarwal^2^, Rakesh K. Gupta^2^, Liying W. Rojanasakul^1^

*^1^ Health Effects Laboratory Division, National Institute for Occupational Safety and Health, Morgantown, WV*

*^2^ Biomedical and Chemical Engineering, West Virginia University, Morgantown, WV*

**Supplementary Table 1.** THP-1 cell culture medium physical parameters for dynamic light scattering measurements of nanoclay particles (n = 3).

| **Parameter** | **RPMI-1640 + 10% FBS** |
| --- | --- |
| Density (g/cm^3^) | 1.00662 |
| Dynamic Viscosity (cP) | 0.75379 |
| Refractive Index | 1.3359 |

**Supplementary Table 2.** Particle percent interference with LDH assay in RPMI cell culture media (n = 4).

| **µg/cm^2^** | **CloisNa** | **Clois30B** | **I-CloisNa** | **I-Clois30B** | **CS** |
| --- | --- | --- | --- | --- | --- |
| LDH only | 0 | 0 | 0 | 0 | 0 |
| 0.02 | -0.32 | 5.77 | -3.43 | -7.22 | -0.02 |
| 0.06 | -9.54 | -5.81 | -11.94 | -4.42 | -12.55 |
| 0.2 | -6.35 | 0.44 | -10.48 | -11.64 | -8.10 |
| 0.6 | -1.88 | -8.06 | -12.72 | -9.72 | -4.02 |
| 2 | 11.07 | -1.15 | -6.99 | -8.20 | -0.76 |
| 6 | 6.57 | -1.29 | -5.67 | 1.26 | -4.32 |
| 20 | 11.68 | 1.94 | -1.28 | -1.99 | -0.28 |

**Supplementary Table 3.** Length and width (µm) measurements from pre- and post-incinerated nanoclays suspended in THP-1 cell culture media (n = 3 independent experiments).

|  | **Cloisite Na** | |  | **Cloisite 30B** | |  | **I-CloisNa** | |  | **I-Clois30B** | |  | **Crystalline Silica** | |
| --- | --- | --- | --- | --- | --- | --- | --- | --- | --- | --- | --- | --- | --- | --- |
|  | **Length** | **Width** |  | **Length** | **Width** |  | **Length** | **Width** |  | **Length** | **Width** |  | **Length** | **Width** |
| Median (µm) | 0.294 | 0.174 |  | 0.304 | 0.183 |  | 1.955 | 1.258 |  | 1.476 | 0.903 |  | 0.724 | 0.468 |
| 1st Quartile (µm) | 0.217 | 0.128 |  | 0.133 | 0.085 |  | 0.889 | 0.538 |  | 0.752 | 0.525 |  | 0.502 | 0.337 |
| 3rd Quartile (µm) | 0.441 | 0.294 |  | 1.124 | 0.766 |  | 3.824 | 2.215 |  | 3.062 | 1.967 |  | 1.111 | 0.696 |
| Skewness | 4.563 | 3.56 |  | 4.418 | 4.199 |  | 2.546 | 2.241 |  | 2.545 | 2.716 |  | 3.010 | 3.282 |
| Kurtosis | 28.838 | 17.414 |  | 22.640 | 20.181 |  | 9.243 | 6.506 |  | 8.389 | 9.684 |  | 13.091 | 15.268 |
| Geometric Mean | 0.313 | 0.191 |  | 0.409 | 0.276 |  | 1.834 | 1.148 |  | 1.568 | 1.037 |  | 0.775 | 0.503 |
| Mean (µm) | 0.379 | 0.237 |  | 1.270 | 0.857 |  | 2.872 | 1.731 |  | 2.392 | 1.636 |  | 0.953 | 0.618 |
| Standard Dev | 0.329 | 0.201 |  | 2.676 | 1.791 |  | 2.951 | 1.716 |  | 2.560 | 1.885 |  | 0.761 | 0.503 |
| N | 182 | 182 |  | 193 | 193 |  | 253 | 253 |  | 371 | 371 |  | 437 | 437 |
| Standard Error | 0.024 | 0.015 |  | 0.193 | 0.129 |  | 0.186 | 0.108 |  | 0.133 | 0.098 |  | 0.036 | 0.024 |
| Median L:W Ratio | 1.686 | - |  | 1.665 | - |  | 1.554 | - |  | 1.634 | - |  | 1.548 | - |

**Supplementary Table 4.** Endotoxin levels (EU/ml) in prepared stock solutions of nanoclay particles suspended in sterile MilliQ water (n=3). Limit of detection (LOD) was 0.01 EU/ml.

| **mg/ml** | **CloisNa** | **Clois30B** | **I-CloisNa** | **I-Clois30B** | **CS** |
| --- | --- | --- | --- | --- | --- |
| 0.01 | <LOD | <LOD | <LOD | <LOD | <LOD |
| 0.1 | <LOD | <LOD | <LOD | <LOD | <LOD |

**Supplementary Table 5**. Cytokine secretion response following pre- and post-incinerated organomodified nanoclay exposure to differentiated THP-1 cells in the presence of LPS.

|  | | **Control** | | |  | | **Cloisite Na** | | | | | | | | | | | | |  | **Cloisite 30B** | | | | | | | | | | | |  |  |
| --- | --- | --- | --- | --- | --- | --- | --- | --- | --- | --- | --- | --- | --- | --- | --- | --- | --- | --- | --- | --- | --- | --- | --- | --- | --- | --- | --- | --- | --- | --- | --- | --- | --- | --- |
| **Cytokine** | | **0 µg/cm^2^** | | | |  | | | **0.06 µg/cm^2^** | | | **0.6 µg/cm^2^** | | | | | | **6 µg/cm^2^** | | | | **0.06 µg/cm^2^** | | | **0.6 µg/cm^2^** | | | | | **6 µg/cm^2^** | | | |  |
| IL-6 | | 9.71 ±0.58 | | |  | | 10.6 ±.5 | | | | **12.0 ±0.5** | | | | | | **7.12 ±.37** | | |  | **12.1 ±.5** | | | 11.2 ±0.5 | | | | | 9.30 ±.41 | | |  |  |  |
| IFN-y | | 35.4 ±1.1 | | |  | | 36.1 ±2.1 | | | | 38.5 ±1.1 | | | | | | 33.0 ±1.7 | | |  | 41.3 ±.9 | | | 40.4 ±1.4 | | | | | 30.2 ±1.8 | | |  |  |  |
| IL-1ra | | 8784 ±406 | | |  | | 8459 ±492 | | | | 9277 ±684 | | | | | | **5827 ±507** | | |  | **10890 ±799** | | | **11780 ±166** | | | | | **5674 ±125** | | |  |  |  |
| IL-5 | | 31.0 ±.8 | | |  | | 35.1 ±1.7 | | | | **37.3 ±.9** | | | | | | 34.4 ±.6 | | |  | **36.4 ±1.3** | | | 35.7 ±.5 | | | | | 34.5 ±.6 | | |  |  |  |
| GM-CSF | | 4.68 ±.13 | | |  | | 5.26 ±.33 | | | | **5.62 ±.11** | | | | | | 5.28 ±.08 | | |  | **5.47 ±.18** | | | 5.25 ±.13 | | | | | 5.23 ±.15 | | |  |  |  |
| TNFa | | 16440 ±2063 | | |  | | 17900 ±4233 | | | | 18960 ±2653 | | | | | | 4056 ±293 | | |  | 20530 ±1589 | | | 16670 ±774 | | | | | **9413 ±147** | | |  |  |  |
| RANTES | | 8172 ±1884 | | |  | | 7731 ±1101 | | | | 6208 ±389 | | | | | | 36.4 ±5.2 | | |  | 7794 ±1152 | | | 7291 ±66 | | | | | 4799 ±443 | | |  |  |  |
| IL-2 | | 69.2 ±2.7 | | |  | | 74.8 ±4.1 | | | | 76.2 ±4.8 | | | | | | **47.8±1.4** | | |  | 81.8 ±4.1 | | | 78.2 ±2.0 | | | | | 66.1 ±1.8 | | |  |  |  |
| IL-1b | | 774.5 ±86.5 | | |  | | 1095 ±139 | | | | **2894 ±64** | | | | | | **4492 ±153** | | |  | 898.3 ±47.3 | | | 989.2 ±24.8 | | | | | **2071 ±77** | | |  |  |  |
| Eotaxin | | 10.1 ±.3 | | |  | | 11.3 ±.3 | | | | **11.6 ±.2** | | | | | | 11.1 ±.2 | | |  | **11.2 ±.2** | | | 11.0 ±.1 | | | | | **11.4 ±.1** | | |  |  |  |
| FGFb | | 44.6 ±1.3 | | |  | | 49.9 ±1.9 | | | | **52.9 ±.7** | | | | | | 50.1 ±.4 | | |  | **50.3 ±1.1** | | | **49.8 ±.4** | | | | | **52.7 ±.7** | | |  |  |  |
| VEGF | | 251.1 ±15.9 | | |  | | 278.6 ±28.9 | | | | 281 ±12.1 | | | | | | **355.3 ±7.3** | | |  | 303.7 ±19.6 | | | 303.7 ±19.3 | | | | | 263.2 ±5.2 | | |  |  |  |
| PDGF-bb | | 353.6 ±9.9 | | |  | | 378.7 ±20.1 | | | | **415.6 ±5.8** | | | | | | **410.7 ±5.2** | | |  | **399.8 ±13.3** | | | **401.9 ±6.9** | | | | | 387.1 ±2.0 | | |  |  |  |
| IP-10 | | 853.7 ±88.6 | | |  | | 707.5 ±104 | | | | 535.2 ±92.4 | | | | | | **57.2 ±1.4** | | |  | **1283 ±88.7** | | | 928.7 ±71.3 | | | | | **252.7 ±23.0** | | |  |  |  |
| IL-13 | | 0.572 ±.039 | | |  | | 0.704±.076 | | | | **0.971 ±.010** | | | | | | 0.539±.020 | | |  | 0.683 ±.044 | | | 0.706 ±.019 | | | | | **1.04 ±.05** | | |  |  |  |
| IL-4 | | 4.87 ±.23 | | |  | | 5.49 ±.30 | | | | **5.92 ±.18** | | | | | | 5.65 ±.13 | | |  | **5.74 ±.19** | | | **5.57 ±.05** | | | | | **5.71 ±.09** | | |  |  |  |
| MCP-1 | | 97.8 ±3.5 | | |  | | 89.0 ±6.3 | | | | 104.6 ±8.1 | | | | | | **22.1 ±.8** | | |  | 121.2 ±6.7 | | | 93.5 ±7.2 | | | | | **29.7 ±1.5** | | |  |  |  |
| IL-8 | | 82575±26,007 | | |  | | >2.27E6 | | | | 1.56E6±0.7E6 | | | | | | 32167±6117 | | |  | 1.60E6±0.7E6 | | | 7.50E5±3.1E5 | | | | | >2.27E6 | | |  |  |  |
| MIP-1a | | 1801±173 | | |  | | 1590±167 | | | | 1691 ±103 | | | | | | 1760±44 | | |  | 1784±19 | | | 2234±100 | | | | | 1569±135 | | |  |  |  |
| IL-10 | | 4.11±.25 | | |  | | 4.44 ±.26 | | | | 4.67 ±.31 | | | | | | **2.26 ±.16** | | |  | 5.06 ±.13 | | | 4.47 ±.13 | | | | | 4.47 ±.35 | | |  |  |  |
| G-CSF | | 129.5 ±10.2 | | |  | | 129.1 ±9.9 | | | | 151.6 ±8.8 | | | | | | **72.3 ±3.4** | | |  | 155.6 ±9.3 | | | 145.9 ±9.2 | | | | | 98.1 ±4.2 | | |  |  |  |
| IL-15 | | 70.0 ±.9 | | |  | | 75.5 ±3.1 | | | | **79.5 ±.8** | | | | | | **81.9 ±2.4** | | |  | **77.5 ±1.5** | | | **75.8 ±.7** | | | | | 72.5 ±1.0 | | |  |  |  |
| IL-7 | | 6.81±1.48 | | |  | | **13.8 ± 1.8** | | | | **29.8 ±1.0** | | | | | | 7.93 ±1.1 | | |  | 9.02±.94 | | | 9.29 ±.98 | | | | | **20.7 ±.4** | | |  |  |  |
| IL-12p70 | | 2.14±.07 | | |  | | 2.32 ±.20 | | | | 2.67 ±.08 | | | | | | 2.38 ± 0 | | |  | 2.63 ±.12 | | | 2.55 ±.11 | | | | | 2.53 ±.13 | | |  |  |  |
| IL-17a | | 69.6 ±3.4 | | |  | | 78.9 ±5.3 | | | | **89.2 ±2.0** | | | | | | **87.1 ±1.0** | | |  | **82.3 ±3.6** | | | **82.7 ±2.0** | | | | | **83.8 ±1.2** | | |  |  |  |
| IL-9 | | 38.4 ±1.3 | | |  | | 41.2 ±.7 | | | | 41.3 ±1.7 | | | | | | **17.6 ±.8** | | |  | 41.5 ±1.6 | | | 41.2 ±.7 | | | | | 37.6 ±2.2 | | |  |  |  |
| Values represent pg/ml concentrations.  Bold values represent significant values compared to unexposed control (p ≤ 0.05; n = 3).  * Significant by Χ^2^ test and the Dunn Method post-hoc compared to Control. | | | | | | | | | | | | | | | | | | | | | | | | | | |  |  |  |  |  |  |  |  |
|  | **Control** | |  | **I-Cloisite Na** | | | | | | | | |  | | **I-Cloisite 30B** | | | | | | | | |  | | **Crystalline Silica** | | | | | | | | |
| **Cytokine** | **0 µg/cm^2^** | |  | **0.06 µg/cm^2^** | | | | **0.6 µg/cm^2^** | | **6 µg/cm^2^** | | | |  | | **0.06 µg/cm^2^** | | | **0.6 µg/cm^2^** | | | | **6 µg/cm^2^** |  | | **0.06 µg/cm^2^** | | **0.6 µg/cm^2^** | | | **6 µg/cm^2^** | | | |
| IL-6 | 9.71 ±0.58 | |  | 9.73 ±.83 | | | | 8.06 ±.98 | | 10.2 ±.05 | | | |  | | 8.90 ±.16 | | | 8.47 ±.34 | | | | 9.80 ±.41 |  | | 8.47 ±.31 | | 8.11 ±.12 | | | 8.33 ±.48 | | | |
| IFN-y | 35.4 ±1.1 | |  | 37.2 ±2.3 | | | | 32 ±3.7 | | 38.0 ±1.0 | | | |  | | 36.4 ±.60 | | | 35.7±.5 | | | | 37.8 ±1.1 |  | | 35.2 ±.6 | | 34.5 ±1.3 | | | 35.0 ±.3 | | | |
| IL-1ra | 8784 ±406 | |  | 9516 ±1657 | | | | 8187 ±635 | | 10040 ±622 | | | |  | | 10340 ±372 | | | 10630 ±878 | | | | 10690 ±189 |  | | 10630 ±492 | | 10650 ±684 | | | 10930 ±507 | | | |
| IL-5 | 31.0 ±.8 | |  | 30.9 ±1.6 | | | | 28.3 ±1.7 | | 32.9 ±.7 | | | |  | | 29.4 ±.6 | | | 29.5 ±.1 | | | | 31.9 ±1.3 |  | | 27.8 ±1.2 | | 27.3 ±.2 | | | 28.2 ±.3 | | | |
| GM-CSF | 4.68 ±.13 | |  | 4.81 ±.32 | | | | 4.34 ±.15 | | 4.89 ±.12 | | | |  | | 4.67 ±.12 | | | 4.52 ±.10 | | | | 4.93 ±.14 |  | | 4.36 ±.08 | | 4.29 ±.03 | | | 4.43 ±.06 | | | |
| TNFa | 16440 ±2063 | |  | 12810 ±3590 | | | | 9642 ±1576 | | 13230 ±509 | | | |  | | 10410 ±556 | | | 9880 ±681 | | | | 11290 ±639 |  | | 8325 ±571 | | 7650 ±398 | | | 8097 ±3 | | | |
| RANTES | 8172 ±1884 | |  | 5242 ±1165 | | | | 7205 ±639 | | 10590 ±1812 | | | |  | | 7401 ±611 | | | 8672 ±848 | | | | 9086 ±303 |  | | 10600 ±865 | | 7887 ±267 | | | 8769 ±126 | | | |
| IL-2 | 69.2 ±2.7 | |  | 65.3 ±3.0 | | | | 61.4 ±3.5 | | 70.5 ±1.1 | | | |  | | 60.6 ±2.3 | | | 59.4 ±2.1 | | | | 66.7 ±2.6 |  | | **54.1±1.8** | | **52.3 ±.6** | | | **56.7 ±.8** | | | |
| IL-1b | 774.5 ±86.5 | |  | 617.6 ±63.3 | | | | 584.3 ±80.2 | | 829.3 ±53.9 | | | |  | | 529.5 ±15.9 | | | 539.7 ±16.5 | | | | 740.5 ±48.3 |  | | 434.7 ±6.0 | | 433.7 ±18.0 | | | 582.3 ±19.2 | | | |
| Eotaxin | 10.1 ±.3 | |  | 10.0 ±.7 | | | | 9.37 ±.55 | | 10.4 ±.47 | | | |  | | 9.19 ±.15 | | | 9.31 ±.03 | | | | 10.06 ±.27 |  | | **8.83 ±.36** | | **8.81 ±.18** | | | 9.11 ±.15 | | | |
| FGFb | 44.6 ±1.3 | |  | 44.6 ±1.3 | | | | 42.7±1.7 | | 46.9 ±1.0 | | | |  | | 42.2 ±.1 | | | 42.0 ±.7 | | | | 46.1 ±.6 |  | | 40.3 ±.3 | | **39.2 ±.6*** | | | 41.6 ±.4 | | | |
| VEGF | 251.1 ±15.9 | |  | 245.1 ±22.7 | | | | 208.4 ±21.6 | | 268.9 ±7.3 | | | |  | | 249.1 ±5.1 | | | 242.6 ±12.9 | | | | 286.7 ±19.4 |  | | 249.6 ±9.1 | | 267.1 ±11.2 | | | 244.8 ±17.6 | | | |
| PDGF-bb | 353.6 ±9.9 | |  | 343 ±17.9 | | | | 330.7 ±12.0 | | 360.3 ±7.2 | | | |  | | 323.4 ±5.9 | | | 323.3 ±8.7 | | | | 358.3 ±7.4 |  | | **298 ±3.6** | | **306.2 ±4.7** | | | **321.8 ±5.3** | | | |
| IP-10 | 853.7 ±88.6 | |  | 892.6 ±53.1 | | | | 811.1 ±15.7 | | 849.4 ±84.3 | | | |  | | 773.9 ±62.1 | | | 774.7 ±53.6 | | | | 732.9 ±54.9 |  | | 818.6 ±55.2 | | 677.9 ±14.4 | | | **388.7 ±27.5** | | | |
| IL-13 | 0.572 ±.039 | |  | 0.571 ±.078 | | | | 0.746 ±.114 | | 0.594 ±.059 | | | |  | | 0.584 ±.030 | | | 0.550 ±.023 | | | | 0.639 ±.038 |  | | 0.573 ±.020 | | 0.640 ±0 | | | 0.503 ±.080 | | | |
| IL-4 | 4.87 ±.23 | |  | 4.69 ±.25 | | | | 4.35 ±.30 | | 5.11 ±.08 | | | |  | | 4.40 ±.10 | | | 4.28 ±.14 | | | | 4.93 ±.05 |  | | **4.11 ±.12** | | 4.14 ±.02 | | | 4.42 ±.06 | | | |
| MCP-1 | 97.8 ±3.5 | |  | 109.7 ±12.3 | | | | 90.1 ±15.7 | | 112.1 ±11.3 | | | |  | | 97.9 ±64 | | | 97.2 ±9.6 | | | | 104.2 ±5.4 |  | | 93.7 ±7.5 | | 91.6 ±4.8 | | | 84.4 ±7.0 | | | |
| IL-8 | 82575±26,007 | |  | 1.51E6±0.7E6 | | | | 1.52E6 ±0.7E6 | | 7.98E5±7.4E5 | | | |  | | 7.86E5 ±7.41E5 | | | 8.28E5 ±7.2E5 | | | | >2268000 |  | | 8.39E5±7.2E5 | | 8.46E5±7.11E5 | | | >2.27E6 | | | |
| MIP-1a | 1801±173 | |  | 1353 ±263 | | | | 1812 ±88 | | 1658 ±94 | | | |  | | 1792 ±331 | | | 1882 ±268 | | | | 1924 ±194 |  | | 1873 ±213 | | 1673 ±60 | | | 1800 ±230 | | | |
| IL-10 | 4.11 ±.25 | |  | 4.07 ±.63 | | | | 3.36 ±.16 | | 4.29 ±.08 | | | |  | | 3.99 ±.2 | | | 3.87 ±.14 | | | | 4.26 ±.16 |  | | 4.20 ±.22 | | 4.14 ±.21 | | | 4.11 ±.15 | | | |
| G-CSF | 129.5 ±10.2 | |  | 116.1 ±22.0 | | | | 104.7 ±7.4 | | 119.7 ±5.6 | | | |  | | 113 ±3.7 | | | 113.8 ±7.5 | | | | 120.1 ±8.9 |  | | 127.2 ±4.8 | | 103.1 ±7.6 | | | 119.8 ±9.2 | | | |
| IL-15 | 70.0 ±.9 | |  | 68.5 ±5.8 | | | | 66.9 ±1.6 | | 72.2 ±2.3 | | | |  | | **64.5 ±1.4** | | | **65.1 ±.4** | | | | 72.8 ±.7 |  | | **61.6 ±1.4** | | **62.2 ±.1** | | | 67.7 ±.9 | | | |
| IL-7 | 6.81±1.48 | |  | 6.83 ±1.10 | | | | 11.3 ±3.9 | | 8.49 ±.55 | | | |  | | 7.94 ±.55 | | | 6.84 ±.56 | | | | 7.94 ±.55 |  | | 5.72 ± 0 | | 7.36 ±1.67 | | | 5.71 ±.98 | | | |
| IL-12p70 | 2.14±.07 | |  | 2.22 ±.13 | | | | 2.05 ±.17 | | 2.09 ±.08 | | | |  | | 2.03 ±.02 | | | 1.93 ±.11 | | | | 2.18 ±.08 |  | | 1.97 ±.04 | | 1.89 ±.07 | | | 1.93 ±.09 | | | |
| IL-17a | 69.6 ±3.4 | |  | 67.9 ±2.0 | | | | 62.6 ±4.0 | | 73.7 ±1.9 | | | |  | | 62.5 ±1.8 | | | 63.0 ±.8 | | | | 70.6 ±.9 |  | | 56.3 ±.4 | | 56.9 ±.2 | | | 64.7 ±.7 | | | |
| IL-9 | 38.4 ±1.3 | |  | 37.1 ±.4 | | | | 39.5 ±.2 | | 40.4 ±.4 | | | |  | | 38.4 ±.5 | | | 39.2 ±.4 | | | | 41.3 ±.3 |  | | 37.0 ±1.2 | | 37.5 ±.7 | | | 37.6 ±.8 | | | |
| Values represent pg/ml concentrations.  Bold values represent significant values compared to unexposed control (p ≤ 0.05; n =3).  * Significant by Χ^2^ test and the Dunn Method post-hoc compared to Control. | | | | | | | | | | | | | | | | | | | | | | | | | | |  |  |  |  |  |  |  |  |

**Supplementary Table 6**. Cytokine secretion following pre- and post-incinerated ONC exposure to differentiated THP-1 macrophage cells.

|  | **Control** |  | **CloisNa** | | |  | **Clois30B** | | |  |
| --- | --- | --- | --- | --- | --- | --- | --- | --- | --- | --- |
| **Cytokine** | **0 µg/cm^2^** |  | **0.06 µg/cm^2^** | **0.6 µg/cm^2^** | **6 µg/cm^2^** |  | **0.06 µg/cm^2^** | **0.6 µg/cm^2^** | **6 µg/cm^2^** | |
| MIP-1β | 64.16 ±1.47 |  | 88.25 ±3.56 | **134.6 ±7.8** | **183.3 ±9.7** |  | 90.39 ±2.53 | 203.8 ±96.4 | 119.5 ±4.4 | |
| IL-6 | 0.85±.03 |  | 1.11 ±.08 | **1.74 ±.09** | **4.23 ±.07** |  | 1.18 ±.06 | 1.93 ±.58 | **1.95 ±.15*** | |
| IFN-γ | 8.98 ±.44 |  | 9.65 ±.54 | **11.87 ±.77** | **17.38 ±.29** |  | 10.44 ±.38 | 12.49 ±1.98 | 10.74 ±.38 | |
| IL-1ra | 4497 ±225 |  | 4769 ±149 | 4750 ±326 | 3566 ±212 |  | 4986 ±194 | 4785 ±526 | **2945 ±134** | |
| IL-5 | 6.47 ±.24 |  | 7.63 ±.28 | **11.71 ±.55** | **22.86 ±.39** |  | 8.10 ±.17 | 10.74 ±2.46 | **12.17 ±.67** | |
| GM-CSF | 1.38 ±.04 |  | 1.56 ±.03 | **2.38 ±.06** | **3.96 ±.10** |  | 1.61 ±.02 | 2.08 ±.3.2 | **2.36 ±.13*** | |
| TNFα | 59.81 ±1.61 |  | 80.08 ±3.21 | 105.7 ±10.2 | **425.2 ±53.2*** |  | 85.65 ±7.92 | 202.8 ±115.7 | 109.6 ±6.7 | |
| RANTES | 467.1 ±19.8 |  | **554.8 ±15.8** | **388.2 ±22.1** | **13.94 ±.58** |  | 557.8 ±8.0 | 849.1 ±225.5 | 364.2 ±16.8 | |
| IL-2 | 5.68 ±.26 |  | 6.76 ±.09 | 10.6 ±.55 | **22.6 ±.21*** |  | 7.30 ±.05 | 10.69 ±2.87 | **11.33 ±.31*** | |
| IL-1β | 132.7 ±2.8 |  | 163.8 ±8.2 | 326.4 ±20.8 | **2274 ±106.6*** |  | 169.8 ±4.3 | 277.6 ±84.0 | **403 ±31.2*** | |
| Eotaxin | 2.43 ±.06 |  | **2.98 ±.04** | **4.28 ±.10** | **8.63 ±.11** |  | 3.17 ±.11 | 4.35 ±.84 | **4.72 ±.26*** | |
| FGFβ | 16.58 ±.23 |  | 18.44 ±.31 | **25.34 ±.75** | **44.76 ±.48** |  | 20.17 ±.47 | 24.72 ±3.94 | **29.83 ±1.01*** | |
| VEGF | 178.7 ±4.0 |  | 209.4 ±12.9 | 180.4 ±8.4 | **224.7 ±10.1** |  | 203.3 ±13.5 | 200.7 ±11.9 | 148.9 ±8.6 | |
| PDGF-ββ | 114.2 ±1.4 |  | **127.2 ±3.0** | **181.9 ±2.6** | **336.6 ±3.7** |  | 125.6 ±5.2 | 180.3 ±28.4 | **190.9 ±7.3*** | |
| IP-10 | 447.2 ±38.4 |  | **304.8 ±30.6** | **116.9 ±18.3** | **46.27 ±.4** |  | 466.2 ±35.3 | 549.6 ±63.4 | 285.1 ±34.3 | |
| IL-13 | 0.185 ±.053 |  | 0.187 ±.027 | 0.264 ±.050 | 0.388 ±.024 |  | 0.172 ±.042 | 0.226 ±.034 | 0.339 ±.025 | |
| IL-4 | 1.27 ±.04 |  | 1.47 ±.02 | 2.23 ±.07 | **4.51 ±.11*** |  | 1.50 ±.06 | 2.10 ±.41 | **2.44 ±.12*** | |
| MCP-1 | 23.54 ±.67 |  | 25.06 ±1.28 | **33.22 ±1.20** | 19.12 ±.53 |  | 26.4 ±.47 | 37 ±8.13 | 15.94 ±.64 | |
| IL-8 | 3154 ±156 |  | 4308 ±80 | **6997 ±651*** | 3007 ±216 |  | 3959 ±311 | **6300 ±837*** | 5293 ±114 | |
| MIP-1α | 97.2 ±9.4 |  | 124.2 ±8.1 | 465.6 ±100.9 | **1577 ±75.6** |  | 162.1 ±8.9 | **528.5 ±204.6*** | **407 ±28.8*** | |
| IL-10 | 0.560 ±.066 |  | 0.884 ±.064 | **1.076 ±.064** | **1.139 ±.127** |  | 0.755 ±.065 | 1.071 ±.353 | 0.884 ±.064 | |
| G-CSF | 24.96 ±2.01 |  | 34.23 ±4.39 | 36.15 ±5.07 | 34.41 ±2.66 |  | 32.76 ±4.87 | 46.54 ±12.5 | 30.07 ±6.53 | |
| IL-15 | 24.97 ±.88 |  | 26.28 ±.87 | **39.77 ±1.58** | **68.79 ±.40** |  | 28.43 ±.84 | 36.54 ±4.38 | **39.17 ±2.03*** | |
| IL-7 | 0.92 ±.65 |  | 2.82 ±.60 | **5.72 ±0*** | 5.14 ±1.13 |  | 3.33 ±1.61 | 2.17 ±1.08 | 5.43 ±.75 | |
| IL-12p70 | 0.443 ±.044 |  | 0.618 ±.075 | **0.748 ±0** | **1.762 ±.072** |  | 0.574 ±.045 | 0.747 ±.129 | **0.919 ±.043*** | |
| IL-17α | 18.5 ±.2 |  | 22.77 ±.16 | **35.39 ±.42** | **71.98 ±2.01** |  | 22.65 ±.91 | 32.33 ±8.11 | **38.33 ±2.52** | |
| IL-9 | 11.67 ±.24 |  | **13.56 ±.31** | **14.03 ±.54** | **8.63 ±.37** |  | 13.38 ±.39 | **15.84 ±1.59*** | 12.61 ±.10 | |

Values represent pg/ml concentrations.

Bold values represent significant values compared to unexposed control (p ≤ 0.05; n = 3).

* Significant by Χ^2^ test and the Dunn Method post-hoc compared to Control

|  | **Control** | **I-CloisNa** | | |  | **I-Clois30B** | | |  | **Crystalline Silica** | | |
| --- | --- | --- | --- | --- | --- | --- | --- | --- | --- | --- | --- | --- |
| **Cytokine** | **0 µg/cm^2^** | **0.06 µg/cm^2^** | **0.6 µg/cm^2^** | **6 µg/cm^2^** |  | **0.06 µg/cm^2^** | **0.6 µg/cm^2^** | **6 µg/cm^2^** |  | **0.06 µg/cm^2^** | **0.6 µg/cm^2^** | **6 µg/cm^2^** |
| MIP-1β | 64.16 ±1.47 | 82.3 ±4.36 | 79.51 ±2.95 | **114.5*** **±24.0** |  | 69.85 ±1.67 | 74.49 ±3.47 | **90.45 ±3.29** |  | 115 ±44 | 72.31 ±2.90 | **86.85* ±8.90** |
| IL-6 | 0.85±.03 | **1.04 ±.05** | **1.03±.01** | **1.31 ±.04** |  | 0.923 ±.110 | 0.886 ±.019 | 1.146 ±.037 |  | 1.118 ±.200 | 0.885 ±.067 | 1.081 ±.057 |
| IFN-γ | 8.98 ±.44 | 9.71 ±.20 | 9.68 ±.42 | 9.85 ±.19 |  | 8.32 ±.46 | 8.58 ±.27 | 9.45 ±.44 |  | 9.81 ±1.04 | 9.48 ±.45 | 9.71 ±.64 |
| IL-1ra | 4497 ±225 | 4688 ±199 | 4294 ±234 | 4035 ±150 |  | 4391 ±427 | 4398 ±120 | 4378 ±254 |  | 4905 ±92 | 4808 ±194 | 4711 ±281 |
| IL-5 | 6.47 ±.24 | **7.55** ±.12 | 7.39 ±.22 | **8.61 ±.24** |  | 6.26 ±.61 | 6.67 ±.04 | 7.90 ±.24 |  | 7.61 ±1.09 | 6.54 ±.45 | 7.39±.35 |
| GM-CSF | 1.38 ±.04 | 1.56 ±.03 | 1.49 ±.08 | 1.69 ±.09 |  | 1.31 ±.06 | 1.46 ±.06 | **1.61 ±.02** |  | 1.60 ±.23 | 1.39 ±.03 | 1.53 ±.05 |
| TNFα | 59.8 ±1.6 | 71.6 ±2.5 | 69.8 ±2.3 | **94.2* ±16.5** |  | 61.5 ±2.4 | 64.2 ±1.1 | **82.2* ±.3** |  | 105 ±40.2 | 65.2 ±3.6 | 86.5 ±7.5 |
| RANTES | 467.1 ±19.8 | 518.2 ±34.7 | 529.1 ±8.0 | 493.9 ±75.6 |  | 505 ±12.5 | 523.5 ±2.9 | 425.8 ±13.5 |  | 625 ±138.3 | 507.7 ±16.5 | 570.5 ±90.0 |
| IL-2 | 5.68 ±.26 | 6.27 ±.30 | 6.22 ±.356 | **7.84 ±.41** |  | 5.68 ±.26 | 5.45 ±.13 | **7.11 ±.10** |  | 7.29 ±1.13 | 6.02 ±.37 | 6.67 ±.18 |
| IL-1β | 132.7 ±2.8 | 156.6 ±8.6 | 159.7 ±3.3 | **206.8 ±8.4** |  | 133.4 ±10.2 | 131.9 ±1.55 | 191.6 ±8.5 |  | 161.9 ±20.8 | 138.9 ±4.67 | 162.5 ±1.5 |
| Eotaxin | 2.43 ±.06 | 2.99 ±.10 | 2.87 ±.17 | **3.17 ±.21** |  | 2.54 ±.13 | 2.63 ±.09 | **3.10* ±.13** |  | 2.87 ±.30 | 2.56 ±.09 | 2.95 ±.07 |
| FGFβ | 16.6 ±.2 | 18.8 ±.7 | 18.3 ±.8 | **20.6 ±.8** |  | 16.5 ±.6 | 16.2 ±.3 | **19.0 ±.6** |  | 18.23 ±1.8 | 16.8 ±.3 | 18.4 ±.3 |
| VEGF | 178.7 ±4.0 | 180.7 ±2.4 | 160.3 ±2.0 | 183 ±7.7 |  | **119.9 ±16.0** | **114.9 ±3.5** | 140.9 ±13.2 |  | **128.7 ±13.8** | **127.4 ±10.1** | **101.9 ±1.3** |
| PDGF-ββ | 114.2 ±1.4 | 127.2 ±3.0 | 114.9 ±4.2 | **145.3 ±9.3** |  | 118 ±5.6 | 110.2 ±1.6 | 131.6 ±5.4 |  | 123.6 ±14.2 | 115.7 ±3.4 | 127.2 ±1.5 |
| IP-10 | 447.2 ±38.4 | 510.9 ±55.0 | 601.1 ±55.9 | 513.1 ±77.2 |  | 443.5 ±36.1 | 384.7 ±41.6 | 522.9 ±15.4 |  | 528.1 ±44.3 | 435.3 ±18.2 | 245.1 ±4.0 |
| IL-13 | 0.185 ±.053 | 0.179 ±.096 | 0.129 ±.050 | 0.187 ±.027 |  | 0.156 ±.057 | 0.129 ±.050 | 0.146 ±.014 |  | 0.214 ±0 | 0.129 ±e.053 | 0.133 ±.050 |
| IL-4 | 1.27 ±.04 | 1.49 ±.05 | 1.44 ±.03 | **1.67 ±.08** |  | 1.22 ±.08 | 1.20 ±.02 | **1.48 ±.03** |  | 1.48 ±.16 | 1.27 ±.05 | 1.40 ±.07 |
| MCP-1 | 23.5 ±.7 | 26.2 ±.8 | 27.3 ±2.1 | 27.9 ±3.3 |  | 26.7 ±2.1 | 25.9 ±.3 | 27.5 ±1.3 |  | 34.5 ±6.3 | 27.2 ±1.2 | 29.2 ±1.6 |
| IL-8 | 3154 ±156 | 3509 ±195 | 3268 ±239 | 5210 ±1649 |  | 3057 ±383 | 3409 ±258 | 3843 ±121 |  | 6194 ±3417 | 2809 ±267 | 3368 ±520 |
| MIP-1α | 97.2 ±9.4 | 117.9 ±16.1 | 134.9 ±9.2 | 436.7 ±340.1 |  | 93.16 ±9.4 | 120.5 ±6.7 | 101.7±13.5 |  | 440.3 ±341.4 | 112.4 ±11.5 | 265.7 ±52.0 |
| IL-10 | 0.560 ±.066 | 0.753 ±.172 | 0.689 ±.131 | 0.626 ±0 |  | 0.423 ±.198 | 0.428 ±0 | 0.689 ±.172 |  | 0.689 ±.172 | 0.360 ±.068 | 0.559 ±.131 |
| G-CSF | 25.0±2.0 | 32.3 ±3.6 | 33.7 ±2.7 | 28.0 ±3.1 |  | 26.8 ±4.7 | 32.4 ±.4 | 22.0 ±1.6 |  | 38.9 ±6.8 | 28.9 ±1.8 | 28.6 ±4.6 |
| IL-15 | 25.0±.9 | 28.2 ±.5 | 28.3 ±.9 | **29.5 ±1.4** |  | 23.3 ±1.0 | 25.1 ±1.2 | 28.0 ±1.5 |  | 27.9 ±2.5 | 25.7 ±.8 | 29.2 ±1.5 |
| IL-7 | 0.92 ±.65 | 2.17 ±1.08 | 3.12 ±.89 | 2.82 ±.60 |  | 1.26 ±.56 | 0.92 ±.65 | 0.92 ±.65 |  | 1.57 ±.65 | 0.92 ±.65 | 1.57 ±.65 |
| IL-12p70 | 0.443 ±.044 | 0.574 ±.044 | 0.574 ±.087 | 0.618 ±.075 |  | 0.487 ±.076 | 0.4874±0 | 0.661 ±.087 |  | 0.574 ±.115 | 0.355 ±0 | 0.509 ±.058 |
| IL-17α | 18.5 ±.2 | 21 ±1.0 | 21.6 ±.3 | **25.6 ±1.2** |  | 18.2 ±.9 | 18.6 ±.2 | 23.8 ±1.1 |  | 21.5 ±3.0 | 18.9 ±.7 | 21.6 ±.3 |
| IL-9 | 11.7 ±.24 | **13.5** ±.4 | 12.6 ±.2 | 12.4 ±5 |  | 11.5 ±.5 | **13.3 ±.4** | 12.8 ±.3 |  | 13.8 ±1.2 | 12.7 ±.7 | 13.7 ±.8 |

Values represent mean pg/ml concentrations ±SE.

Bold values represent significant values compared to unexposed control (p ≤ 0.05; n = 3).

* Significant by Χ^2^ test and the Dunn Method post-hoc compared to Control.

**Supplementary Table 7.** Non-LPS and LPS stimulated THP-1 macrophage cytotoxicity metrics at three different nanoclay particle dosing schemes correlated to BAL cell differentials following dose equivalent aspiration exposure in male C57Bl/6J mice. Data from all five tested particles were included in the analysis.

|  |  |  | **LDH** |  |  | **WST** |  |  | **Live Cell** |  |  |
| --- | --- | --- | --- | --- | --- | --- | --- | --- | --- | --- | --- |
| **LPS status** | **Day** | **Metric** | **.06, 0.6 µg/cm^2^** | **.2, 2 µg/cm^2^** | **.6, 6 µg/cm^2^** | **.06, 0.6 µg/cm^2^** | **.2, 2 µg/cm^2^** | **.6, 6 µg/cm^2^** | **.06, 0.6 µg/cm^2^** | **.2, 2 µg/cm^2^** | **.6, 6 µg/cm^2^** |
|  | *BALF Total Cell Count* | | |  |  |  |  |  |  |  |  |
| No LPS | Day 1 | r | **0.64** | 0.24 | 0.16 | -0.13 | -0.38 | -0.55 | -0.036 | -0.37 | -0.41 |
|  |  | P Value | **0.035*** | 0.48 | 0.63 | 0.71 | 0.25 | 0.083 | 0.92 | 0.26 | 0.21 |
|  | Day 7 | r | **0.63** | 0.046 | 0.36 | -0.34 | -0.48 | **-0.79** | -0.39 | -0.58 | **-0.8** |
|  |  | P Value | **0.039*** | 0.89 | 0.27 | 0.31 | 0.13 | **0.004*** | 0.23 | 0.06 | **0.003*** |
|  | *BALF Monocyte Counts* | | |  |  |  |  |  |  |  |  |
| No LPS | Day 1 | r | 0.5 | 0.45 | 0.28 | -0.17 | -0.48 | -0.55 | 0.28 | -0.25 | -0.21 |
|  |  | P Value | 0.12 | 0.16 | 0.40 | 0.61 | 0.13 | 0.083 | 0.40 | 0.45 | 0.54 |
|  | Day 7 | r | **0.64** | 0.08 | 0.34 | -0.4 | -0.54 | **-0.81** | -0.35 | -0.6 | **-0.76** |
|  |  | P Value | **0.035*** | 0.81 | 0.31 | 0.22 | 0.089 | **0.003*** | 0.30 | 0.051 | **0.006*** |
|  | *BALF Neutrophil Counts* | | |  |  |  |  |  |  |  |  |
| No LPS | Day 1 | r | **0.61** | -0.14 | 0.12 | -0.082 | -0.28 | **-0.64** | -0.73 | -0.55 | **-0.69** |
|  |  | P Value | **0.047*** | 0.69 | 0.73 | 0.81 | 0.40 | **0.035*** | 0.19 | 0.08 | **0.019*** |
|  | Day 7 | r | **0.69** | -0.13 | 0.28 | -0.082 | -0.39 | **-0.77** | **-0.61** | **-0.71** | **-0.9** |
|  |  | P Value | **0.019*** | 0.71 | 0.40 | 0.81 | 0.23 | **0.005*** | **0.047*** | **0.015*** | **<0.001*** |
|  | *BALF Lymphocyte Counts* | | |  |  |  |  |  |  |  |  |
| No LPS | Day 1 | r | 0.073 | 0.23 | -0.25 | 0.027 | 0.036 | -0.19 | -0.2 | -0.35 | -0.38 |
|  |  | P Value | 0.83 | 0.50 | 0.45 | 0.97 | 0.92 | 0.57 | 0.56 | 0.30 | 0.25 |
|  | Day 7 | r | **0.63** | 0.06 | 0.22 | -0.4 | -0.6 | **-0.79** | -0.25 | **-0.65** | **-0.65** |
|  |  | P Value | **0.039*** | 0.85 | 0.52 | 0.22 | 0.051 | **0.004*** | 0.47 | **0.032*** | **0.032*** |
|  | *BALF Total Cell Count* | | |  |  |  |  |  |  |  |  |
| LPS+ | Day 1 | r | n/a | n/a | n/a | -0.33 | **-0.71** | **-0.64** | -0.33 | **-0.65** | **-0.65** |
|  |  | P Value | n/a | n/a | n/a | 0.33 | **0.015*** | **0.035*** | 0.33 | **0.029*** | **0.029*** |
|  | Day 7 | r | n/a | n/a | n/a | **-0.64** | **-0.97** | **-0.85** | -0.36 | **-0.87** | **-0.84** |
|  |  | P Value | n/a | n/a | n/a | **0.035*** | **<0.001*** | **0.001*** | 0.27 | **0.001*** | **0.001*** |
|  | *BALF Monocyte Counts* | | |  |  |  |  |  |  |  |  |
| LPS+ | Day 1 | r | n/a | n/a | n/a | -0.25 | -0.51 | -0.58 | -0.07 | -0.45 | -0.54 |
|  |  | P Value | n/a | n/a | n/a | 0.45 | 0.11 | 0.06 | 0.83 | 0.16 | 0.089 |
|  | Day 7 | r | n/a | n/a | n/a | **-0.67** | **-0.96** | **-0.84** | -0.35 | **-0.86** | **-0.82** |
|  |  | P Value | n/a | n/a | n/a | **0.023*** | **<0.001*** | **0.001*** | 0.28 | **0.001*** | **0.002*** |
|  | *BALF Neutrophil Counts* | | |  |  |  |  |  |  |  |  |
| LPS+ | Day 1 | r | n/a | n/a | n/a | -0.37 | **-0.85** | **-0.73** | -0.47 | **-0.8** | **-0.74** |
|  |  | P Value | n/a | n/a | n/a | 0.26 | **0.001*** | **0.011*** | 0.14 | **0.003*** | **0.001*** |
|  | Day 7 | r | n/a | n/a | n/a | -0.42 | **-0.94** | **-0.85** | -0.42 | **-0.92** | **-0.86** |
|  |  | P Value | n/a | n/a | n/a | 0.20 | **<0.001*** | **0.001*** | 0.20 | **<0.001*** | **0.002*** |
|  | *BALF Lymphocyte Counts* | | |  |  |  |  |  |  |  |  |
| LPS+ | Day 1 | r | n/a | n/a | n/a | 0.1 | -0.29 | -0.3 | -0.46 | -0.42 | -0.51 |
|  |  | P Value | n/a | n/a | n/a | 0.77 | 0.39 | 0.37 | 0.15 | 0.20 | 0.11 |
|  | Day 7 | r | n/a | n/a | n/a | -0.59 | **-0.91** | **-0.8** | -0.35 | **-0.85** | **-0.75** |
|  |  | P Value | n/a | n/a | n/a | 0.056 | **<0.001*** | **0.003*** | 0.28 | **0.001*** | **0.007*** |
|  |  |  |  |  |  |  |  |  |  |  |  |

Bold font and * indicate significant Spearman correlation coefficients (p ≤0.05).

**Supplementary Table 8.** THP-1 macrophage inflammasome marker response correlation to BAL cell differentials in male C57Bl/6J mice following pre- and post-incinerated nanoclay particles.

|  |  | **Cathespin B release** | | | **Gasdermin D cleavage** | | **IL-1β / LPS +** | | **IL-1 β / LPS -** | |
| --- | --- | --- | --- | --- | --- | --- | --- | --- | --- | --- |
| Day | Metric | **0.06, 0.6 µg/cm^2^** | **0.2, 2 µg/cm^2^** | **0.6, 6 µg/cm^2^** | **0.6, 6 µg/cm^2^** | **2, 20 µg/cm^2^** | **0.06, 0.6 µg/cm^2^** | **0.6, 6 µg/cm^2^** | **0.06, 0.6 µg/cm^2^** | **0.6, 6 µg/cm^2^** |
| *BALF Total Cell Count* | | |  |  |  |  |  |  |  |  |
| Day 1 | r | 0.33 | -0.10 ^a^ | -0.17 ^a^ | 0.49 ^a^ | 0.58 ^a^ | **0.62** | **0.71** | 0.25 ^a^ | **0.66** ^a^ |
|  | P Value | 0.33 | 0.77 | 0.61 | 0.13 | 0.06 | **0.04*** | **0.014*** | 0.45 | **0.026*** |
| Day 7 | r | 0.45 | 0.35 ^a^ | 0.14 ^a^ | **0.66** ^a^ | **0.85** ^a^ | **0.7** | **0.8** | 0.58 ^a^ | **0.86** ^a^ |
|  | P Value | 0.16 | 0.30 | 0.69 | **0.029*** | **0.001*** | **0.017*** | **0.003*** | 0.06 | **<0.001*** |
| Day 28 | r | **0.76** | 0.57 ^a^ | 0.39 ^a^ | 0.42 ^a^ | **0.81** ^a^ | 0.11 | 0.23 | 0.49 ^a^ | **0.62** ^a^ |
|  | P Value | **0.007*** | 0.07 | 0.23 | 0.20 | **0.003*** | 0.75 | 0.49 | 0.13 | **0.043*** |
| *BALF Monocyte Counts* | | |  |  |  |  |  |  |  |  |
| Day 1 | r | 0.06 | -0.31 ^a^ | -0.15 ^a^ | 0.64 ^a^ | 0.58 ^a^ | **0.75** | **0.77** | 0.25 ^a^ | **0.66** ^a^ |
|  | P Value | 0.86 | 0.36 | 0.67 | **0.035*** | 0.06 | **0.0073*** | **0.0052*** | 0.47 | **0.029*** |
| Day 7 | r | 0.45 | 0.32 ^a^ | 0.15 ^a^ | **0.72** ^a^ | **0.84** ^a^ | **0.7** | **0.8** | **0.62** ^a^ | **0.89** ^a^ |
|  | P Value | 0.16 | 0.34 | 0.65 | **0.013*** | **0.001*** | **0.017*** | **0.0029*** | **0.043*** | **<0.001*** |
| Day 28 | r | **0.67** | 0.39 ^a^ | 0.12 ^a^ | 0.45 ^a^ | **0.79** ^a^ | 0.28 | 0.41 | 0.38 ^a^ | **0.70** ^a^ |
|  | P Value | **0.024*** | 0.23 | 0.73 | 0.17 | **0.004*** | 0.41 | 0.20 | 0.25 | **0.017*** |
| *BALF Neutrophil Counts* | | |  |  |  |  |  |  |  |  |
| Day 1 | r | 0.43 | 0.21 ^a^ | -0.01 ^a^ | 0.38 ^a^ | 0.58 ^a^ | 0.35 | 0.57 | 0.40 ^a^ | **0.67** ^a^ |
|  | P Value | 0.19 | 0.59 | 0.77 | 0.25 | 0.06 | 0.29 | 0.069 | 0.22 | **0.023*** |
| Day 7 | r | 0.54 | 0.32 ^a^ | -0.05 ^a^ | 0.51 ^a^ | **0.66** ^a^ | 0.51 | **0.65** | 0.50 ^a^ | **0.75** ^a^ |
|  | P Value | 0.085 | 0.34 | 0.89 | 0.11 | **0.026*** | 0.11 | **0.032*** | 0.12 | **0.009*** |
| Day 28 | r | 0.55 | 0.24 ^a^ | 0.03 ^a^ | 0.09 ^a^ | 0.54 ^a^ | 0.02 | 0.18 | 0.0 ^a^ | 0.33 ^a^ |
|  | P Value | 0.081 | 0.48 | 0.94 | 0.79 | 0.009 | 0.94 | 0.59 | 1.00 | 0.33 |
| *BALF Lymphocyte Counts* | | |  |  |  |  |  |  |  |  |
| Day 1 | r | -0.39 | -0.25 ^a^ | 0.01 ^a^ | 0.13 ^a^ | 0.05 ^a^ | 0.27 | 0.28 | 0.12 ^a^ | 0.13 ^a^ |
|  | P Value | 0.24 | 0.47 | 0.98 | 0.71 | 0.90 | 0.42 | 0.41 | 0.73 | 0.71 |
| Day 7 | r | 0.41 | 0.23 ^a^ | -0.01 ^a^ | **0.64** ^a^ | **0.76** ^a^ | **0.72** | **0.89** | 0.59 ^a^ | **0.85** ^a^ |
|  | P Value | 0.21 | 0.50 | 0.98 | **0.035*** | **0.006*** | **0.013*** | **0.0003*** | 0.06 | **0.001*** |
| Day 28 | r | **0.65** | 0.41 ^a^ | 0.23 ^a^ | 0.35 ^a^ | **0.76** ^a^ | 0.18 | 0.25 | 0.34 ^a^ | 0.58 ^a^ |
|  | P Value | **0.029*** | 0.21 | 0.50 | 0.77 | **0.006*** | 0.61 | 0.45 | 0.31 | 0.06 |

All data were log-transformed to meet normalization assumption for Pearson correlation analyses.

^a^ Non-parametric data; Spearman’s coefficient correlation analysis was performed.

Bold font and * indicate significant correlations (p ≤0.05).

**Supplementary Table 9.** Spearman correlation coefficients of equivalent dose LPS stimulated THP-1 cells (0.6 or 6 µg/cm^2^) vs. C57Bl/6J BALF (300 µg) at Day 1 and 7 post-exposure for 16 pro-inflammatory cytokines.

|  | **CloisNa** | **Clois30B** | **I-CloisNa** | **I-Clois30B** | **CS** | |
| --- | --- | --- | --- | --- | --- | --- |
| *24 hr 0.6 µg/cm^2^ vs. Day 1 300 µg/lung* | | | | | |  |
| r | -0.26 | n/a | n/a | n/a | n/a | |
| p-value | 0.34 | n/a | n/a | n/a | n/a | |
| *24 hr 6 µg/cm^2^ vs. Day 1 300 µg/lung* | | | | | |  |
| r | -0.25 | n/a | n/a | n/a | n/a | |
| p-value | 0.36 | n/a | n/a | n/a | n/a | |
| *24 hr 0.6 µg/cm^2^ vs. Day 7 300 µg/lung* | | | | | |  |
| r | -0.23 | n/a | n/a | n/a | n/a | |
| p-value | 0.39 | n/a | n/a | n/a | n/a | |
| *24 hr 6 µg/cm^2^ vs. Day 7 300 µg/lung* | | | | | | |
| r | -0.31 | n/a | n/a | n/a | n/a | |
| p-value | 0.25 | n/a | n/a | n/a | n/a | |

n/a indicates correlations not performed due to treatments producing minimal statistically significant THP1 cytokines.

**Supplementary Table 10.** Correlation coefficients for each cytokine expression level between non-LPS stimulated differentiated THP-1 macrophage vs. BAL from male C57Bl/6J lung.

|  | **0.6 µg/cm^2^ 24 h *vs.* 300 µg/lung Day 1** | | **6 µg/cm^2^ 24 h *vs.* 300 µg/lung Day 1** | | **0.6 µg/cm^2^ 24 h *vs.* 300 µg/lung Day 7** | |
| --- | --- | --- | --- | --- | --- | --- |
|  | **r** | **p value** | **r** | **p value** | **r** | **p value** |
| GM-CSF | 0.63 | 0.25 | 0.82 | 0.09 | 0.05 | 0.93 |
| IL-8 / KC | -0.20 | 0.75 | 0.28 | 0.65 | 0.11 | 0.86 |
| TNFα | -0.76 | 0.14 | -0.43 | 0.47 | 0.11 | 0.86 |
| IL-12p70 | 0.75 | 0.15 | **0.96** | **0.010*** | 0.84 | 0.07 |
| MCP1 | 0.30 | 0.62 | 0.39 | 0.52 | 0.81 | 0.10 |
| IL-1β | -0.25 | 0.68 | 0.05 | 0.94 | **0.92** | **0.028*** |
| IL-8 / MIP2 | -0.74 | 0.15 | 0.29 | 0.63 | 0.56 | 0.33 |
| VEGF | 0.36 | 0.56 | 0.78 | 0.12 | 0.52 | 0.37 |
| IL-6 | 0.50 | 0.39 | **0.97** | **0.007*** | 0.85 | 0.06 |
| IL-10 | 0.60 | 0.28 | 0.73 | 0.17 | **0.94** | **0.017*** |
| IL-13 | 0.19 | 0.75 | 0.35 | 0.56 | 0.81 | 0.10 |
| MIP-1α | -0.85 | 0.07 | 0.57 | 0.32 | 0.63 | 0.26 |
| MIP-1β | -0.74 | 0.15 | -0.67 | 0.21 | 0.46 | 0.44 |
| FGFβ | 0.65 | 0.24 | 0.80 | 0.10 | 0.50 | 0.39 |
| Eotaxin | 0.66 | 0.34 | **0.93** | **0.023*** | 0.87 | 0.055 |
| PDGF-ββ | 0.70 | 0.18 | **0.93** | **0.022*** | **0.94** | **0.017*** |

Bold font and * indicates those log-transformed correlations that were statistically significant (p ≤ 0.05).
